# Supplementary material for: Quality of informal care among informal caregivers of people with dementia: A latent profile and ROC analysis
Source: PLoS One. 2026 Apr 8;21(4):e0346557. doi: 10.1371/journal.pone.0346557 (PMC13061180; doi:10.1371/journal.pone.0346557)
Supplement: S1 Table — (PDF) [file pone.0346557.s001.pdf]

S1 Table. Univariate analysis of different latent profile (N=213).

Variable	Category	Profile			F/x^2	P
		Low QoC (n=66) M±SD or n (%)	Moderate QoC (n=95) M±SD or n (%)	High QoC (n=52) M±SD or n (%)		
PwD						
Age (years)		77.52±9.98	76.92±9.29	77.69±9.10	0.140	0.869
Gender	Male	38(57.6)	59(62.1)	20(38.5)	7.859	**0.020**
	Female	28 (42.4)	36 (37.9)	32 (61.5)		
Education	Primary school or below	48 (72.7)	41 (43.2)	23 (44.2)	17.708	**0.001**
	Secondary school	17 (25.8)	44 (46.3)	26 (50.0)		
	High school or above	1 (1.5)	10 (10.5)	3 (5.8)		
Type of dementia	Alzheimer	40 (60.6)	54 (56.8)	34 (65.4)	3.933	0.415
	Vascular dementia	24 (36.4)	34 (35.8)	13 (25.0)		
	Other	2 (3.0)	7 (7.4)	5 (9.6)		
Years since diagnosis	～1	12 (18.2)	30 (31.6)	16 (30.8)	8.669	0.070
	～5	30 (45.4)	41 (43.1)	28 (53.8)		
	≥5	24 (36.4)	24 (25.3)	8 (15.4)		
Number of children	0	1 (1.5)	0 (0)	0 (0)	2.726	0.924
	1	16 (24.2)	23 (24.2)	11 (21.1)		
	2	23 (34.9)	30 (31.6)	17 (32.7)		
	3	26 (39.4)	42 (44.2)	24 (46.2)		
ADL	No	1 (1.5)	4 (4.2)	12 (23.1)	36.483	**< 0.001**
	Mild	14 (21.2)	22 (23.2)	23 (44.2)		
	Moderate	13 (19.7)	17 (17.9)	6 (11.5)		
	Severe	38 (57.6)	52 (54.7)	11 (21.2)		
Residence	Rural	39 (59.1)	18 (18.9)	13 (25.0)	30.373	**< 0.001**
	Urban and town	27 (40.9)	77 (81.1)	39 (75.0)		
Informal caregivers						
Age (years)		60.06±15.20	58.48±14.00	58.85±14.01	0.242	0.786
Gender	Male	23 (34.8)	31 (32.6)	21 (40.4)	0.891	0.641
	Female	43 (65.2)	64 (67.4)	31 (59.6)		
Education	Primary school or below	25 (37.9)	13 (13.7)	7 (13.5)	20.888	**< 0.001**
	Secondary school	29 (43.9)	44 (46.3)	21 (40.4)		
	High school or above	12 (18.2)	38 (40.0)	24 (46.1)		
Relationship with PwD	Spouse	25 (37.9)	33 (34.7)	18 (34.6)	5.423	0.247
	Children	28 (42.4)	53 (55.8)	29 (55.8)		
	Other	13 (19.7)	9 (9.5)	5 (9.6)		
Living with PwD	Yes	53 (80.3)	66 (69.5)	42 (80.8)	3.476	0.176
	No	13 (19.7)	29 (30.5)	10 (19.2)		
Length of care (year)	～1	14 (21.2)	39 (41.1)	17 (32.7)	13.455	**0.009**
	～5	30 (45.5)	38 (40.0)	29 (55.8)		
	≥5	22 (33.3)	18 (18.9)	6 (11.5)		
Affordability of living	Difficult	41 (62.1)	15 (15.8)	6 (11.6)	54.090	**< 0.001**

expenses	Somewhat difficult	14 (21.2)	30 (31.6)	23 (44.2)		
	Not difficult	11 (16.7)	50 (52.6)	23 (44.2)		
Self-rated health	Poor	48 (72.7)	64 (67.4)	28 (53.8)	4.808	0.090
	Good	18 (27.3)	31 (32.6)	24 (46.2)		
Depression	Yes	41 (62.1)	26 (27.4)	2 (3.8)	47.078	**< 0.001**
	No	25 (37.9)	69 (72.6)	50 (96.2)		
Quality of pre-illness relationship		9.95±2.74	11.98±2.49	13.33±2.26	27.231	**< 0.001**
Perceived overload		11.95±2.29	9.01±2.51	7.81±2.38	48.616	**< 0.001**
Social support		34.38±4.16	40.20±6.46	43.69±7.38	44.947	**< 0.001**
