# Supplementary material for: Quality of informal care among informal caregivers of people with dementia: A latent profile and ROC analysis
Source: PLoS One. 2026 Apr 8;21(4):e0346557. doi: 10.1371/journal.pone.0346557 (PMC13061180; doi:10.1371/journal.pone.0346557)
Supplement: S2 Table — (PDF) [file pone.0346557.s002.pdf]

S2 Table. Criterion values and coordinates of ROC Curve (validation subsample).

Cut-off point	Sensitivity	Specificity	Youden's index
14	0.985	0.857	0.842
**15**	**0.985**	**0.943**	**0.928**
16	0.897	1.000	0.897
17	0.721	1.000	0.721

Note: Estimates in italics are the suggested optimal cut-off points; ROC: Receiver Operating Characteristic.
