# Supplementary figures and images for: Quality of informal care among informal caregivers of people with dementia: A latent profile and ROC analysis
Source: PLoS One. 2026 Apr 8;21(4):e0346557. doi: 10.1371/journal.pone.0346557 (PMC13061180; doi:10.1371/journal.pone.0346557)

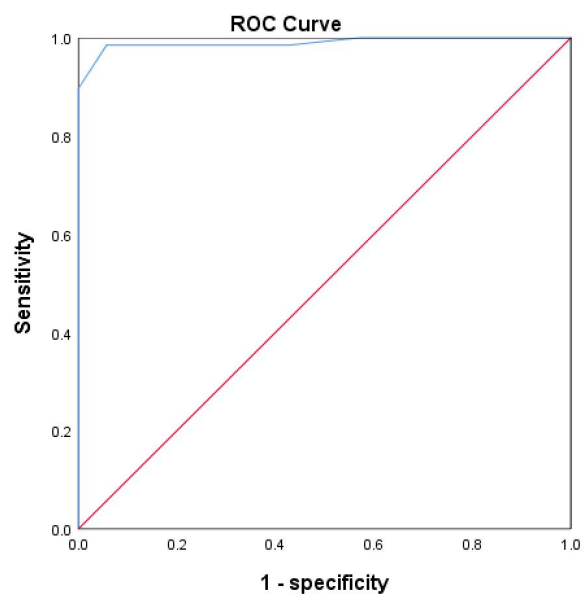

**S1 Fig. ROC curve of the Exemplary Care Scale for classifying low and high quality of care.**

Supplement: S1 Fig — (PDF) [file pone.0346557.s003.pdf]
