# Supplementary material for: Quality of informal care among informal caregivers of people with dementia: A latent profile and ROC analysis
Source: PLoS One. 2026 Apr 8;21(4):e0346557. doi: 10.1371/journal.pone.0346557 (PMC13061180; doi:10.1371/journal.pone.0346557)
Supplement: Attachment — Submitted filename: Suggestions for authors.pdf [file pone.0346557.s007.pdf]

Good morning.

First, I would like to congratulate the authors on their excellent work and on giving me the opportunity to review it. The topic addressed, the quality of care, is relevant, and the authors' approach provides information that may be useful to the scientific community. The effort made by the authors to convey their results is evident.

As a result of my review, I have the following suggestions regarding your manuscript. My suggestions are divided into general suggestions, which affect the manuscript, and specific suggestions, which are described by line number and section of the manuscript to facilitate their location.

I hope to have the opportunity to review your manuscript again with the suggested corrections.

General suggestions regarding the manuscript:

The authors include many phrases in a single paragraph, making the length of the paragraph difficult to read and to maintain the idea of what was being read. To make the manuscript easier to read, it would be advisable to divide the long paragraphs using new paragraphs.

Within the current paragraphs, the sentences often stand alone and are not adequately interconnected, making it difficult to establish a causal link between one sentence and the next. The authors should try to use words at the beginning of sentences that connect the previous sentence to the current one.

The same situation occurs between paragraphs. Authors should try to interconnect paragraphs with bridging words such as “on the other hand,” “likewise,” “along these lines,” and “however” to make them easier to read.

Finally, the language used is sometimes very colloquial, suggesting that some words be replaced with more technical or neutral ones. Likewise, statements are sometimes made that are not supported by the data provided or the bibliography, and it is recommended that these points be reviewed.

Specific suggestions regarding the manuscript:

1.- Introduction:

- Line 53, first paragraph of the Introduction section: the phrase “that results in” should perhaps be modified to use a term such as “is associated with.”

- Line 54: authors should clarify whether the data “152 million in 2050” refers to the global level or to China.

- Line 56: the phrase “In China, the number of PwD accounts for about a quarter of the total number, ranking first in the world [3,4]” could be improved. An alternative phrase would be: “Currently, in terms of global prevalence, one in four PwD reside in China, which ranks first in the world in terms of number of cases.”

- Line 59: The sentence “Informal caregivers are thought to be more familiar with their relative with dementia than formal caregivers, and often provide care that is more in line with the relative's preferences and values” could probably be improved by the authors, using, for example, a sentence such as “This care is based both on the cultural tradition of caring for our elders and on a greater knowledge of their preferences and values than formal caregivers may have or provide.”

- Line 64: Facilitate the connection between separate sentences, using, for example, words such as “In this sense, some studies...”.

- Line 65: The authors could improve the wording of the phrase “Indirectly shortening survival,” for example, by describing this situation in terms such as “situations that, directly or indirectly, end up affecting their life expectancy.”

- Line 73: The authors could improve the wording of the phrase “These dimensions ignored CRs' feelings and wishes, and failing to reflect the meaning of quality home care.” Among the various options for making this improvement, the following example is provided: “These dimensions are not sensitive to the feelings and wishes of CRs and, therefore, do not reflect all the aspects that quality care for PwD living in the community should consider.”

- Line 80: The phrases “This approach conveys to CRs the affirming message that they are ‘loved, respected, and worthy of special attention.’ The message helps maintain the dignity and integrity of CRs, reflecting the concept of ‘person-centered’ care” are redundant with respect to statements in previous phrases and could be simplified, for example, by expressing in the text: “This approach conveys to care recipients the message that they are loved, respected, and worthy of special attention, helping to maintain their dignity and integrity, and holistically reflecting the concept of person-centered care.”

- Line 82: The authors are advised to divide the paragraph at this point, as its current length makes it difficult to read and understand. The new paragraph should connect to the last sentence of the previous paragraph, using words such as “In this line, and based on the conceptual framework of exemplary care.”

- Line 95: The authors should improve the connection between phrases and interconnect the messages that these phrases convey, using phrases such as, for example, "Although much research focuses on the total ECS scale score to assess overall quality of life and the factors that influence it [16-20], this five-variable approach fails to capture individual differences and does not allow for the observation of heterogeneity among different subgroups.

- Line 104: Authors are suggested to insert a paragraph break at this point, as the current length hinders reading and overall comprehension. The new paragraph should connect to the last sentence of the previous paragraph, using words such as “Along the same lines, the latent profile analysis...”.

- Line 113: We recommend modifying the verb forms so as not to take the result of the study for granted. For example, using the following words: “The results of this study could provide important empirical evidence for the subsequent development of precise intervention strategies for different subgroups of caregivers.”

- Line 116: The authors should improve the connection between paragraphs. Using words such as “On the other hand, although the heterogeneity analysis...”

- Line 118: This phrase might better express what is intended if it is stated in the future tense: “To address this practical challenge, we propose to investigate in greater depth through this study the optimal cutoff value of ECS using ROC analysis, using the results of the LPA classification as a criterion.”

The authors could improve the wording and include part of the following paragraph. For example: "This approach would allow clinicians and community professionals to quickly identify high-risk groups and establish action strategies, using only the total score as a reference. The proposed simplification of the scoring process represents a methodological innovation that preserves the advantage of the LPA in identifying heterogeneity and improves its clinical applicability, while also providing empirical evidence for understanding the causes of this heterogeneity."

- Line 124: The hypotheses should come before the objectives and be placed on line 124. The current wording of the paragraph addressing the hypotheses could be improved. An attempt should also be made to interconnect the different paragraphs to make them easier to read. The following wording is proposed as an example to the authors, which they can modify according to their criteria:

"In this context, our hypotheses are: (1) the quality of informal care could be categorized into different latent profiles according to the ECS; (2) the quality of informal care provided by caregivers varies depending on individual characteristics, stressors, and social support. (3) On this basis, and through ROC analysis, it may be possible to establish an optimal cutoff value with high sensitivity and specificity that allows for the identification of different levels of care quality and, specifically, the identification of low-quality care."

The authors should also move and adapt some subsequent phrases included in the objectives paragraph and place them in a new paragraph after the hypothesis paragraph:

“These results would have two practical implications. First, they would provide a scientific basis for developing precise interventions tailored to different subgroups. Second, they could significantly improve the assessment of quality of care and, with it, efficiency.”

Thus, from line 124 onwards, the development of the hypotheses and objectives should follow a chronological order, for example:

"In this context, our hypotheses are: (1) the quality of informal care could be categorized into different latent profiles according to the ECS; (2) the quality of informal care provided by caregivers varies depending on individual characteristics, stressors, and social support. (3) On this basis, and through ROC analysis, it may be possible to establish an optimal cutoff value with high sensitivity and specificity that allows for the identification of different levels of care quality and, specifically, the identification of low-quality care."

“These results would have two practical implications. First, they would provide a scientific basis for developing targeted interventions tailored to different subgroups. Second, they could significantly improve the assessment of care quality and, thereby, efficiency.”

"To respond to these hypotheses, four objectives were set out in this study (perhaps the authors should set out four objectives instead of three, with objective 4 being: "and (4) to propose rapid screening criteria that enable health and social organizations to quickly identify high-risk individuals." I also suggest modifying the wording of objective 3 to include the areas of application where this objective would be relevant: "(3) identify categorization thresholds that allow for application in clinical and community practice through ROC analysis."

Interconnect paragraphs. For example: "To respond to these hypotheses...".

2. Materials and methods

2.1 Design and participants

- Line 143: The authors should expand on the description of the type of study conducted.

- Line 146: The authors should specify the city, province, and country where the study was conducted.

- Line 149: To make the manuscript easier to read, it would be advisable to divide the long paragraphs with full stops. We suggest dividing this paragraph into this line.

- Line 157: To make the manuscript easier to read, it would be advisable to divide the long paragraphs with full stops. We suggest dividing this paragraph into this line.

- Line 165: To make the manuscript easier to read, it would be advisable to divide the long paragraphs with full stops. We suggest dividing this paragraph into this line.

- Line 165: Regarding the new independent paragraph created, "For multinomial logistic regression, the sample size"... it is suggested that the authors improve the wording and interconnect paragraphs. Perhaps the authors should rewrite this paragraph to increase the clarity of what is being presented and maintain a logical sequence. Reposition the bibliography if necessary. As an example of wording, which can be modified by the authors at their discretion: "To meet the objectives of the study, it was taken into account that, within a potential categories model, a sample size of no less than 200 people was necessary [25]. Initially, 248 participants were recruited. However, 35 were excluded due to incomplete responses or other reasons, resulting in 213 valid participants included in the final analysis (response rate of 85.89%). On the other hand, for multinomial logistic regression, the sample size should be at least 5-10 times the number of independent variables [24]. With 20 variables included in this study and considering a potential rate of 20% invalid questionnaires, a minimum of 120 participants was initially required.

2.2 Variables and measures

2.2.1 Dependent variable

- Line 180: The wording of this sentence could be improved, for example: “The ECS is completed by informal caregivers and consists of 11 items that fall into two dimensions: Provide (items 1-5) and Respect (items 6-11).”

2.2.2 Independent variable

Authors should describe the criteria used for the variables listed in Table 1 that may be analyzed in this study, including the levels, scale, range, or method used for classification. Specifically, the levels of education, the criteria used to define cohabitation with the person with dementia, and the criteria used to establish whether caregivers of the person with dementia have the capacity to pay for living expenses.

On the other hand, within the variable of cohabitation with the person with dementia, the authors establish as an inclusion criterion “care for more than 4 hours each day,” without requiring cohabitation in the same home. This may generate a bias, as it includes people who live with the person with dementia 24 hours a day and those who only do so for one-sixth of the day, a bias that should be considered both in the results and in the limitations of study. The authors should justify how a person who does not live with the patient can be their caregiver.

Perhaps a new variable should be established:

Caregiver's relationship with the dementia patient: The type of relationship between the caregiver and the dementia patient was evaluated, establishing the following groups of caregivers: 1: children; 2: partner; and 3: other types of caregivers.

Regarding the categories currently included in the manuscript:

Quality of pre-illness relationship:

- Line 193: The first word is lowercase. It should read “Caregivers.” The authors should define the intermediate values (2, 3).

Activities of daily living (ADL):

- Line 198: I have a question about the last sentence in this section: "The scale was completed by informal caregivers based on the condition of PwD." The authors imply that the primary caregivers were the ones responsible for completing the Barthel index. This point should be clarified, explaining whether the interviewers asked family members about the situation of the person with dementia performing basic activities of daily living and whether the interviewers filled out the questionnaire. Or the questionnaire was self-completed by the caregivers, i.e., family members were given a booklet with the Barthel Index questions, and the caregivers completed the questionnaire independently, according to the situation of their family member with dementia.

On another note, I believe it is necessary to open a debate on this point. The Barthel Index was initially designed to assess functional capacity and has numerous classifications. The authors reference that they use the classification proposed by Shah in 1989, which is an adaptation of the Barthel Index for a specific setting, but the classification they propose is not one of the most widely used. Furthermore, since then, numerous studies have been conducted on the usefulness of the Barthel Index, proposing various cut-off points for levels of functional dependence. Multiple studies have also been conducted associating the different levels of functional dependence of the Barthel Index with mortality. Others associate each item of the Barthel Index with mortality. All of this has been done with the aim of analyzing the association between the ability to perform basic activities of daily living and life expectancy. As a result of all these studies, there is a certain consensus that below a Barthel index of 40, there is a significant increase in the risk of mortality, the risk of hospitalization, the risk of complications during hospitalization, and the risk of complications upon discharge.

Although no global consensus has been published on the levels used in population studies, it seems logical to suggest that the most up-to-date data with the strongest evidence should be used to facilitate comparisons between studies and the performance of meta-analyses. Therefore, although the classic 5-level classification remains in force (total dependence (Barthel <20); severe dependence (Barthel 20-35); moderate dependence (Barthel 40-60), mild dependence (Barthel 65-95), and independence (Barthel 100 (from 90 according to other authors)), it is advisable to collect data on the risk of mortality and complications associated with functional dependence and use a 4-level classification: severe dependence (Barthel 0-35); moderate dependence (Barthel 40-60), mild dependence (Barthel 65-95); and independence (Barthel 100). Therefore, I suggest that the authors consider reclassifying their patients according to this scale and

performing the analyses based on it. This modification can be justified in the manuscript by establishing that these levels are those that have been shown to be associated with the risk of mortality and the risk of complications. On the other hand, by de facto dichotomizing the ADL variable (the number of patients with moderate dependence in their sample is small), differences may be observed that were not detected with the previous classification.

Perceived Overload:

- Line 203: The wording could be improved. For example: “To assess the perceived overload of caregivers, the overload scale, consisting of 4 items, was used.” In addition, the authors should define in this section each value that an item can take and not just values 1 and 4.

Physical health:

- Line 209: Fair health is not the same as poor health. If both items are included to create a binary variable, the categories could be defined, for example, as “impaired” (1 = very poor, poor, fair) and “good” (2 = good, very good).

2.3 Statistics analysis

- Line 229: The authors should improve the wording of the first phrase.

3. Results

General considerations regarding the Results section:

The authors have a large amount of data, which they include in Table 1, but do not present in the text of the manuscript. I do not know if this is because they are reserving this data for another study on this cohort. If this is not the case, perhaps its presentation in this manuscript would provide relevant information on the results, which could be very useful for other researchers. Information that, when properly related in the text, could not only complement that provided by reading the table, but also allow the interrelationships between the variables in the table to be established. Among other possible aspects that could be addressed are:

- There is no mention that most people with dementia (54.9%) were male. Globally, dementia is more prevalent in women. This is a distinguishing feature of your study. Perhaps it should be mentioned in this section and in the Discussion section.

- There is also no mention of the fact that while people with dementia typically had a low level of education (52.6%), caregivers typically had a high school education (44.1%). This data shows that the social ladder that allows one generation to surpass the previous one is active.

- The couple lives with the person with dementia, but among the children and other non-children, it might be useful to know what percentage of each group lived with the person with dementia.

- It would also be useful to know, among the caregivers who did not live with the person with dementia, whether they had been caring for this person for less than one year, one to five years, or more than five years. Similarly, it would be useful to know whether the time since the diagnosis of dementia was less than one year, one to five years, or more than five years. Perhaps these part-time caregivers have been caring for the person with dementia for less than a year or are caring for people with a recent diagnosis of dementia, data that may influence the overall analysis of the results.

- The average age of caregivers is high. This is probably due to the age of the partners of people with dementia. This aspect should be clarified in this section by the authors, specifying the average age for each group proposed in the definition of variables and in the text of this section.

- It would provide information for the interpretation of the results if the authors analyzed whether there is an association between the level of education of caregivers of people with dementia and the level of difficulty in paying for the living expenses of the person with dementia. If it is observed that a lower level of education is associated with greater difficulty in meeting these expenses, a new line of action could be proposed by social services for this most vulnerable group.

- It would also be interesting to know whether women had greater difficulty in meeting the living expenses of the person with dementia than men. And whether this was associated with their level of education, physical health, or the presence of depression. If confirmed, it would also suggest a course of action by social services for this most vulnerable group.

- On the other hand, the environment, rural or urban, may have implied greater or lesser difficulty in meeting the costs of care.

- Finally, all these results, in addition to being described in the text, could be included in their numerical values in a new table. This could encompass other analyses that, in line with the proposed approach, could be incorporated by the authors.

- Line 276: Table 2: The table legend should include a description of the acronyms so that the table can be read independently, without referring to the text of the manuscript.

- Line 277: Table 3: The description of each profile should be included to avoid having to refer to the text of the manuscript.

- Line 278: Table 4: The legend of the table should include a description of the acronyms so that the table can be read independently, without referring to the text of the manuscript.

3.4 ROC analysis results

- Line 305: The first sentence of this paragraph should be placed within the Material and Methods section, subsection 2.2.1 Dependent variable.

When making this change, it would be advisable to restructure this paragraph. Perhaps with a sentence such as: "Using the standard described in the section on the dependent variable...".

- Line 312: Figure 2: To enable other authors to evaluate the results shown in Figure 2, it would be advisable to include the data table that SPSS, or the program used to produce it, provides when running the process. This table could be included as an appendix to the manuscript.

4. Discussion

General comment:

Sometimes the sentences within a single paragraph are disconnected from each other, giving the impression of loose sentences. The authors should review this aspect and try to interconnect the end of one sentence with the beginning of the next. This can be applied to the entire manuscript. In this section, the connection between one paragraph and the next should also be improved.

On the other hand, there is also a tendency to repeat results or to redescribing the methods used, rather than creating a Discussion about them. Likewise, in the first three paragraphs there are redundant sentences, repetitive with other words, or the same. The authors should synthesize these paragraphs.

In addition, the beginning of the second paragraph of the Discussion section is like the first. The wording of this paragraph should be changed. On the other hand, percentages are added, which might be better moved to the Results section, stating here, for example, that most caregivers of people with dementia have a moderate quality of life, and a high percentage have a poor quality of life. Finally, the first and second paragraphs present a discussion of the results that is partially intermingled. Both paragraphs should be rewritten, attempting to go step by step in the discussion of the results they have.

This new wording could also discuss the possible associations between each profile and having to care for a person with dementia who was severely dependent for basic activities of daily living, or having to care for a person with dementia for more than five years, or what it means for each profile to have low economic resources to ensure the maintenance of the dependent person. In addition to the relationship of each profile with the independent variables included in this study.

Finally, if the analyses suggested in the review of the Results section are carried out, associations may be observed between being a woman, having a lower level of education, having a lower economic status, having greater difficulty in coping with the costs of caring for the person with dementia, and having a poorer quality of life, as well as possibly having a poorer perception of health status and a higher probability of having depressive symptoms. If these hypotheses are confirmed, the authors should perhaps consider conducting an analysis by sex, assessing whether the cutoff point of 15 is valid for male and female caregivers, or whether there are differences in the cutoff point depending on sex.

Specific comments on the submitted text:

- Line 319: Regarding the sentence "Based on Extended Stress Model, this study examined how background variables, primary stressors, caregivers' quality of life, and caregiver resources predicted different profiles of quality of informal care," perhaps the authors, rather than stating what the study did, should interpret what the study found, for example, replacing the word "examined" with "allowed us to observe," if the authors believe that this expression is more appropriate for what they are trying to express. In addition, link the second sentence of this paragraph to the first, using, for example, "On the other hand, based on the Extended Stress Model..."

- Line 325: Regarding the sentence "The cut-off point of the ECS was determined at 15 using ROC analysis, providing an initial reference standard for future screening and intervention studies," this last sentence of the first paragraph of the Discussion section needs to be expanded upon. The authors should try to explain what this new cut-off point implies for the existing situation, both clinically and in terms of social services planning.

- Line 340: The first two sentences of this third paragraph of the manuscript are redundant. It is recommended that the authors summarize them into a single sentence to make the manuscript easier to read.

- Line 363: The wording of the fifth paragraph of the Discussion section could be improved. ADLs are defined by the ability to perform those activities. Therefore, the wording should be along the lines of: "At the same time, when patients with dementia had a greater ability to perform basic activities of daily living, this situation correlated positively with the quality of care, while caregiver burden showed a negative correlation, results that have also been observed in a large number of studies [7,40,41,42,44].". Caregivers experience high levels of stress and overload in the long-term care of older adults with dementia [45], while at the same time having to cope with family and occupational responsibilities. Full-time, long-term care impairs caregivers' mental health and their ability to provide care, further diminishing their quality of life [46,47]. Added to this is the progressive deterioration of the functional capacity of the person with dementia to perform basic activities of daily living, which also contributes significantly to increasing caregiver overload [48]. Or similar wording, depending on the authors' perspectives.

- Line 370: To make the manuscript easier to read, it would be advisable to divide long paragraphs using full stops. We suggest dividing this paragraph into this point.

- Line 370: When dividing the paragraph at this point, the authors should improve the connection between paragraphs by using bridging phrases, such as: "In addition to the factors mentioned, other factors also play a role in this evolutionary process. Research has shown that cognitive impairment...".

- Line 375: The authors should improve the connection between sentences. At this point, for example, among other options, they could use: "This situation is also contributed to by the fact that progressive disability is often accompanied by aphasia and comprehension deficits...".

- Line 377: The wording of this paragraph could be improved. The authors should try to present their discussion by linking the various interpretations of the results obtained and their relationship to other observations made by other authors. In relation to this paragraph, an alternative wording might be: "These challenges can undermine caregivers' patience and lead to inadequate care, so that as the ability of people with dementia to perform ADLs declines, the quality of care may also potentially decline. Therefore, assessing the ability of people with dementia to perform ADLs is an indispensable tool for providing individualized care and for proposing measures to increase these abilities, all with the goal of improving their quality of life and that of their caregivers". NOTE: Look for bibliography to support these aspects. This wording can be modified at the author's discretion.

5. Conclusion

- Line 434: The phrase "better pre-morbid relationships and superior ADL of PwD were predictors of high QoC" could be improved. An alternative wording would be: "better pre-morbid relationships and greater ability to perform activities of daily living (ADL) among people with dementia were predictors of a high quality of life."

- Line 434: Perhaps the appropriate word is "heterogeneous" rather than "homogeneous."

- Line 441: It may be necessary to include new conclusions if the analyses suggested in the previous sections are carried out.
