# Supplementary material for: Quality of informal care among informal caregivers of people with dementia: A latent profile and ROC analysis
Source: PLoS One. 2026 Apr 8;21(4):e0346557. doi: 10.1371/journal.pone.0346557 (PMC13061180; doi:10.1371/journal.pone.0346557)
Supplement: Attachment — Submitted filename: PONE-D-25-38583_R1.pdf [file pone.0346557.s008.pdf]

PLOS ONE

Quality of informal care among informal caregivers of patients with dementia: a latent profile and ROC analysis

--Manuscript Draft--

**Manuscript Number:**	PONE-D-25-38583R1
**Article Type:**	Research Article
**Full Title:**	Quality of informal care among informal caregivers of patients with dementia: a latent profile and ROC analysis
**Short Title:**	Quality of informal care among informal caregivers of patients with dementia
**Corresponding Author:**	Chongqing Shi<br>Wuhan University of Science and Technology<br>Wuhan, CHINA
**Keywords:**	informal caregivers; Patients with dementia; latent profile analysis; Quality of informal care; Extended stress-process model
**Abstract:**	Background: The quality of informal care has become a particularly important issue, as patients with dementia (PwD) often prefer to receive care in home-based, rather than in institutions. However, there is a lack of clinically differentiated intervention programs for populations with different quality of informal care characteristics. Additionally, the absence of clearly thresholds for identifying PwD with low quality of informal care poses a challenge for research and clinical practice. Thus, this study aimed to identify potential profiles of among informal caregivers of PwD, explore influencing factors of different profile, and determine the cut-off point of the Exemplary Care Scale (ECS).<br>Methods ：From 15 July 2023 to 14 July 2024, convenience sampling method was used to select 213 dyads of PwD and their informal caregivers from memory clinic, rehabilitation clinic, and neurological clinic of the tertiary level A hospitals and communities in China. Latent profile analysis (LPA) was used to derive potential profiles. Multinomial logistic regression analysis was conducted to explore influencing factors of quality of informal care in different profiles. Receiver operating characteristic (ROC) analysis was carried out to identify the cut-off value of the ECS.<br>Results: Three quality of care (QoC) profiles were identified: high QoC (24.41%), moderate QoC (44.60%) and low QoC (30.99%). Worse affordability of living expenses, insufficient social support, and higher perceived overload were associated with low QoC. Better quality of pre-illness relationship and superior activities of daily living (ADL) of PwD were associated with high QoC. ROC curve for the ECS identified a cut-off point of 15, demonstrating high sensitivity (0.993) and specificity (0.955).<br>Conclusion s: The quality of informal care has obvious heterogeneity and classification characteristics. Understanding the factors influencing of different quality of informal care and the cut-off point provides a basis for precise interventions.
**Order of Authors:**	Chan Cai<br>Bing Cheng<br>Chongqing Shi<br>Wenli Shi<br>Chenyang Li<br>Cui Liu<br>Jin Sun
**Response to Reviewers:**	Response to Editor:<br>1.I agree with one of the reviewers, that more details are necessary on how authors calculated the sample size. Kindly provide all assumptions for sample size estimation for future research.<br>Revised<br>Thanks very much for your review of our manuscript. In response, we have supplemented and refined the description of the sample size estimation accordingly. For multinomial logistic regression, the sample size should be at least 5-10 times the

number of independent variables [24]. With 20 variables included in this study and accounting for a potential 20% rate of invalid questionnaires, a minimum of 120 participants was initially required. To enhance the accuracy and validation efficacy of the potential category model, a sample size of at least 200 was deemed necessary [25]. Initially, 248 participants were recruited. However, 35 were excluded due to incomplete responses or other reasons, resulting in 213 valid participants included in the final analysis (85.89% response rate). (lines 165-173)

[24] Xu, Y., Chen, Z., Tang, X. et al. Latent profile analysis of nutrition knowledge, attitudes, and practices and their influencing factors in maintenance hemodialysis patients. Sci Rep 15, 17246 (2025). https://doi.org/10.1038/s41598-025-02142-4.

[25] Chen X, Wang Z, Wang S, et al. Latent profiles of ambivalence over emotional expression in young breast cancer patients: A cross-sectional study. Int J Nurs Sci. 2025;12(4):379-385. Published 2025 Jun 16. doi:10.1016/j.ijnss.2025.06.005.

2. The manuscript need careful proofreading. Kindly revise the manuscript carefully for sentence formatting, internal consistency, and punctuation.

Revised. We have carefully checked and revised the manuscript to ensure that the sentence format, internal consistency and punctuation are correct.

Response to Reviewer 1:

1. “However, few studies have examined heterogeneity within groups of caregivers of PwD or the characteristics of quality of care, resulting in a lack of empirical basis for early identification and precise support for high-risk caregivers. Notably, with the rapidly growing population of PwD in China, informal caregivers are experiencing an increasingly heavy burden of care.”

While this statement attempts to establish a research gap, it is vague and lacks specificity and supporting evidence. I recommend reframing the gap more precisely, ideally by identifying exactly what has been understudied and how the current study addresses that gap.

Indeedly, while this statement attempts to establish a research gap, it is vague and lacks specificity and supporting evidence. Thank you for your valuable comments. We have revised clearly specify the under-investigated areas within current research and to explicitly elaborate on how our study is designed to address these specific gaps.

Many researches mainly focus on the total score of ECS scale to assess overall QoC and its influencing factors [16-20]. However, these studies adopt a variable-oriented approach, which fails to capture individual differences and obscures heterogeneity among different subgroups. It is essential to identify distinct patterns of the QoC within the group and to implement targeted interventions based on their different characteristics. Although existing research has identified distinct caregiver profiles associated with different types of low QoC in the elderly [21],

few studies have examined characteristic within groups of caregivers of PwD, resulting in a lack of empirical basis for precise support for high-risk caregivers of PwD supplied low QoC. (lines 95-104)

[21] Yan E, Lai DWL, Sun R, et al. Typology of family caregivers of older persons: A latent profile analysis using elder mistreatment risk and protective factors. J Elder Abuse Negl. 2023;35(1):34-64. doi:10.1080/08946566.2023.2197269

2. The statistical analysis section (2.3) demonstrates an appropriate selection of methods, particularly in applying LPA and utilizing relevant model fit criteria. However, the writing suffers from unclear variable definitions, grammatical issues, and imprecise explanations of key statistical concepts. I suggest restructuring the paragraph for clarity, clearly defining all abbreviations, and correcting misleading interpretations (e.g., regarding entropy). These revisions would enhance the section’s clarity and improve its support for the study analytical rigor and reproducibility.

Revised.

Thank you for your careful checks. We have made a comprehensive revision to the presentation of the statistical analysis section to ensure clarity and accuracy.

Each item score on the ECS serves as explicit indicators to establish a LPA model using Mplus 8.3. The most suitable model was selected based on fit indices [35], including the Akaike Information Criterion (AIC), Bayesian Information Criterion (BIC), and adjusted Bayesian Information Criterion (aBIC), Lo-Mendell-Rubin likelihood ratio test (LMR), Bootstrapped likelihood ratio test (BLRT), and Entropy. Lower values of AIC, BIC, and aBIC indicated a superior model fit. Differences between latent profile models were compared using the LMR and BLRT. A significant P-value suggests that

Powered by Editorial Manager® and ProduXion Manager® from Aries Systems Corporation

the model with k classes fits the data better than the model with k-1 classes. Entropy was used to assess classification accuracy, with values closer to 1 indicating more precise classification; an entropy value ≥0.8 indicates a classification accuracy exceeding 90%. Cohen's d was calculated to further validate the accuracy of the classification (0.2-0.5: small; 0.5-0.8: medium; >0.8: large) [36,37]. (lines 230-242)

3. The discussion section largely restates that there are three QoC groups, but it does not provide a deep interpretation of what distinguishes these profiles, how meaningful the differences are, or how the findings relate to the theoretical framework.
Revised.
Thank you very much for this insightful and critical comment. We have thoroughly revised the Discussion section to address these points as follows, analyzing the differences and significance of the three groups of characteristics, as well as linking the results of this study to the theoretical framework.
We used LPA to categorize the caregivers of PwD into three distinct profiles, i.e., high QoC (24.41%), moderate QoC (44.60%), and low QoC (30.99%). Statistically significant differences were found between the high and moderate QoC profile in terms of pre-illness relationship quality and PwD' ADL. Logistic regression analysis revealed that the high QoC profile was linked to great quality of pre-illness relationship and superior PwD' ADL. Similarly, significant differences in economic status, perceived overload, and social support were observed between the low and moderate QoC profile. The low QoC profile was associated with low economic affordability, high perceived overload, and inadequate social support. These results highlight that tailored strategies based on the distinct characteristics of each caregiver subgroup. Priority should be given to caregivers facing financial strain, high perceived overload, and insufficient social support. (lines 328-339)
The Extended Stress Process Model hints that background variables, primary stressors, quality of life, and caregiver resources collectively influence the quality of informal care. Our study verified that background variables, primary stressors, and caregiver resources impacted the quality of informal care, except caregivers' quality of life. Specifically, affordability of living expenses, pre-illness relationship quality, the ADL of PwD, perceived overload, and social support decided three QoC profiles. These findings provide an innovative perspective on the importance of economic status, relationship quality, caregiving stress, social support in mitigating the deterioration of QoC among caregivers of PwD. (lines 340-348)

4. The discussion does not explain why specific predictors (e.g., ADL, pre-illness relationship) influence care quality profiles, nor does it contextualize the findings by comparing them to prior research. A more critical engagement with existing literature would strengthen the discussion and improve the paper contribution to the field.
Revised.
Thanks for your valuable feedback. We have revised to provide a more thorough explanation of how specific predictors (such as Activities of Daily Living and pre-illness relationship quality) influence care quality profiles, and have contextualized our findings through systematic comparisons with previous studies.
Align with previous studies [15,42], a positive pre-illness relationship serves as a protective factor for QoC. As Steadman et al. demonstrated [43], caregivers reporting higher premorbid relationship satisfaction showed significantly less stress and less reactivity to memory and behavioral problems, and better problem solving skills and more effective communication. Conversely, caregivers with poor pre-illness relationships may perceive caregiving responsibilities as an additional burden, leading to negative attitudes and behaviors [41,43], ultimately compromising the quality of informal care. The finding emphasizes the critical role of caregiving context during care, suggesting that interventions aimed at improving QoC should consider pre-illness relationship quality. (lines 352-362)
The decline in ADL among PwD significantly increases caregiver overload [48]. Research has shown that cognitive impairment or psychiatric symptoms are prevalent in most patients at the time of diagnosis [3], substantially compromising their daily functioning and necessitating caregiver assistance. However, many caregivers of PwD struggle to provide consistent and attentive care due to insufficient care capacity and a lack of care resources [49], ultimately resulting in diminished QoC. Furthermore, disability is often accompanied by aphasia and comprehension deficits, making it difficult for patients to express their needs accurately. These challenges may undermine caregivers' patience and lead to inadequate care. (lines 369-378)

	[43] Steadman PL, Tremont G, Davis JD. Premorbid relationship satisfaction and caregiver burden in dementia caregivers. J Geriatr Psychiatry Neurol. 2007;20(2):115-119. doi:10.1177/0891988706298624<br>[48] Kim, B., Noh, G.O. & Kim, K. Behavioural and psychological symptoms of dementia in patients with Alzheimer's disease and family caregiver burden: a path analysis. BMC Geriatr 21, 160 (2021). https://doi.org/10.1186/s12877-021-02109-w<br>[49] Report on the family survival status of Alzheimer's disease patients in China. Alzheimer's Disease Chinese, 2020-1. 2019.<br>URL: http://weixin.moreedge.cn/bps_2019/index.php<br><br>Response to Reviewer 2:<br>1.It is unclear which approach ultimately led to the final target of >210 participants, even though the sample size calculation section specifies requirements for both LPA (≥150 participants) and multinomial logistic regression (>200 participants). Could you please clarify if the final target was selected based on the higher of the two estimates or if there was another way that you arrived at the number 213?<br>In accordance with the Editor's suggestion, the revisions have been incorporated. Please see Response to Editor Answer No.1 (lines 165-173 in the revised manuscript).<br><br>2.The sample size calculation assumes 3 latent profiles, but this assumption is not supported by previous research or pilot results. Giving a reference will assure readers of its validity, since the assumed number of profiles directly affects the sample size needed for LPA.<br>Revised.<br>Thanks the reviewer for this important comment. The assumption regarding the number of potential profiles in the initial submission was not sufficiently grounded. In response, we have removed the unsupported statement and revised.<br>To enhance the accuracy and validation efficacy of the potential category model, a sample size of at least 200 was deemed necessary [25]. (lines 169-170)<br>[25] Chen X, Wang Z, Wang S, et al. Latent profiles of ambivalence over emotional expression in young breast cancer patients: A cross-sectional study. Int J Nurs Sci. 2025;12(4):379-385. Published 2025 Jun 16. doi:10.1016/j.ijnss.2025.06.005.<br><br>3.It's unclear what is meant by the statement "the mean scores of 11 items of the ECS were used as observed variables." Although it sounds like you averaged the items before performing the analysis, the individual item scores are typically used as the input in latent profile analysis. It appears from Figure 1 that you used the 11-item scores for each individual in the analysis, and then you computed the average scores for each profile to describe them. If this was not what you thought, kindly reword it.<br>Revised.<br>We have corrected the incorrect statement in the manuscript.<br>Each item score on the ECS serves as explicit indicators to establish a LPA model using Mplus 8.3. (lines 230-231)<br><br>4. The ECS cut-off of 15 is determined by the ROC analysis, and this shall be validated in a different sample, which would improve its clinical applicability across different populations, even though the reported sensitivity and specificity are high.<br>Revised.<br>The original expression of the third limitation has been revised to improve its clarity and precision.<br>Third, although this study reported high sensitivity and specificity, the ECS cut-off value of 15 was determined through ROC analysis. Future validation in different samples are needed to enhance the clinical applicability of this threshold. (lines 419-421)
**Additional Information:**	
**Question**	**Response**
**Financial Disclosure**<br><br>Enter a financial disclosure statement that describes the sources of funding for the work included in this submission. Review	The author(s) received no specific funding for this work.

the submission guidelines for detailed requirements. View published research articles from *PLOS ONE* for specific examples.<br><br>This statement is required for submission and **will appear in the published article** if the submission is accepted. Please make sure it is accurate.<br><br>**Funded studies**<br>Enter a statement with the following details:<br>• Initials of the authors who received each award<br>• Grant numbers awarded to each author<br>• The full name of each funder<br>• URL of each funder website<br>• Did the sponsors or funders play any role in the study design, data collection and analysis, decision to publish, or preparation of the manuscript?<br><br>Did you receive funding for this work?	
**Competing Interests**<br><br>Use the instructions below to enter a competing interest statement for this submission. On behalf of all authors, disclose any competing interests that could be perceived to bias this work—acknowledging all financial support and any other relevant financial or non-financial competing interests.<br><br>This statement is required for submission and **will appear in the published article** if the submission is accepted. Please make sure it is accurate and that any funding sources listed in your Funding Information later in the submission form are also declared in your Financial Disclosure statement.<br><br>View published research articles from *PLOS ONE* for specific examples.	The authors have declared that no competing interests exist.

**NO authors have competing interests**<br><br>Enter: *The authors have declared that no competing interests exist.*<br><br>**Authors with competing interests**<br><br>Enter competing interest details beginning with this statement:<br><br>*I have read the journal's policy and the authors of this manuscript have the following competing interests: [insert competing interests here]*<br><br>* typeset	
**Ethics Statement**<br><br>Enter an ethics statement for this submission. This statement is required if the study involved:<br><br>• Human participants<br>• Human specimens or tissue<br>• Vertebrate animals or cephalopods<br>• Vertebrate embryos or tissues<br>• Field research<br><br>Write "N/A" if the submission does not require an ethics statement.<br><br>General guidance is provided below. Consult the submission guidelines for detailed instructions. **Make sure that all information entered here is included in the Methods section of the manuscript.**	The study was approved by the Ethics Committee of TianYou Hospital Affiliated toWuhan University of Science &Technology (LL-2023-07-14-002). After being thoroughly informed of the purpose of the study, all participants provided written informed consent and completed independent questionnaires anonymously

Powered by Editorial Manager® and ProduXion Manager® from Aries Systems Corporation

**Format for specific study types**<br><br>**Human Subject Research (involving human participants and/or tissue)**<br>• Give the name of the institutional review board or ethics committee that approved the study<br>• Include the approval number and/or a statement indicating approval of this research<br>• Indicate the form of consent obtained (written/oral) or the reason that consent was not obtained (e.g. the data were analyzed anonymously)<br><br>**Animal Research (involving vertebrate animals, embryos or tissues)**<br>• Provide the name of the Institutional Animal Care and Use Committee (IACUC) or other relevant ethics board that reviewed the study protocol, and indicate whether they approved this research or granted a formal waiver of ethical approval<br>• Include an approval number if one was obtained<br>• If the study involved *non-human primates*, add *additional details* about animal welfare and steps taken to ameliorate suffering<br>• If anesthesia, euthanasia, or any kind of animal sacrifice is part of the study, include briefly which substances and/or methods were applied<br><br>**Field Research**<br><br>Include the following details if this study involves the collection of plant, animal, or other materials from a natural setting:<br>• Field permit number<br>• Name of the institution or relevant body that granted permission	
**Data Availability**<br><br>Authors are required to make all data underlying the findings described fully available, without restriction, and from the time of publication. PLOS allows rare exceptions to address legal and ethical concerns. See the PLOS Data Policy and FAQ for detailed information.	Yes - all data are fully available without restriction

A Data Availability Statement describing where the data can be found is required at submission. Your answers to this question constitute the Data Availability Statement and **will be published in the article**, if accepted.<br><br>**Important:** Stating 'data available on request from the author' is not sufficient. If your data are only available upon request, select 'No' for the first question and explain your exceptional situation in the text box.<br><br>Do the authors confirm that all data underlying the findings described in their manuscript are fully available without restriction?	
**Describe where the data may be found in full sentences. If you are copying our sample text, replace any instances of XXX with the appropriate details.**<br><br>• If the data are **held or will be held in a public repository**, include URLs, accession numbers or DOIs. If this information will only be available after acceptance, indicate this by ticking the box below. For example: *All XXX files are available from the XXX database (accession number(s) XXX, XXX.*).<br>• If the data are all contained **within the manuscript and/or Supporting Information files**, enter the following: *All relevant data are within the manuscript and its Supporting Information files.*<br>• If neither of these applies but you are able to provide **details of access elsewhere**, with or without limitations, please do so. For example:<br><br>*Data cannot be shared publicly because of [XXX]. Data are available from the XXX Institutional Data Access / Ethics Committee (contact via XXX) for researchers who meet the criteria for access to confidential data.*<br><br>*The data underlying the results presented in the study are available from (include the name of the third party*	The data used or analyzed in this study may be obtained from the corresponding author upon reasonable request.

and contact information or URL).<br>• This text is appropriate if the data are owned by a third party and authors do not have permission to share the data.<br><br>* typeset	
Additional data availability information:	

Powered by Editorial Manager® and ProduXion Manager® from Aries Systems Corporation

Manuscript Click here to access/download;Manuscript;Manuscript.docx

Quality of informal care among informal caregivers of patients with dementia: a latent profile and ROC analysis

Chan Cai[a,b,1], Bing Cheng[a,1], Chongqing Shi[a,*], Wenli Shi[a], Chenyang Li[a], Cui Liu[a], Jin Sun[c]

a. Institute of Nursing Research, Hubei Province Key Laboratory of Occupational Hazard Identification and Control, School of Medicine, Wuhan University of Science and Technology, Wuhan 430065, Hubei Province, China.
b. Department of Nursing, Xiangyang No.1 People's Hospital, Hubei University of Medicine, Xiangyang, 441000
c. Yiling Hospital of Yichang City, Yichang, Hubei, China

* Correspondence should be addressed to Chongqing Shi, E-mail: shichongqing@wust.edu.cn; Fax numbers: 0086-27-68893428.

[1]Chan Cai, Bing Cheng have contributed equally to this work.

Email address: caichan718@163.com(C. Cai); 1040178900@qq.com (B. Cheng); shichongqing@wust.edu.cn (C. Shi); wenli_0824@163.com (W. Shi); 1063467525@qq.com (C. Li); 499263863@qq.com (C, Liu); 79817540@qq.com (J Sun).

Abstract

Background: The quality of informal care has become a particularly important issue, as patients with dementia (PwD) often prefer to receive care in home-based, rather than in institutions. However, there is a lack of clinically differentiated intervention programs for populations with different quality of informal care characteristics. Additionally, the absence of clearly thresholds for identifying PwD with low quality of informal care poses a challenge for research and clinical practice. Thus, this study aimed to identify potential profiles of among informal caregivers of PwD, explore influencing factors of different profile, and determine the cut-off point of the Exemplary Care Scale (ECS).

Methods : From 15 July 2023 to 14 July 2024, convenience sampling method was used to select 213 dyads of PwD and their informal caregivers from memory clinic, rehabilitation clinic, and neurological clinic of the tertiary level A hospitals and communities in China. Latent profile analysis (LPA) was used to derive potential profiles. Multinomial logistic regression analysis was conducted to explore influencing factors of quality of informal care in different profiles. Receiver operating characteristic (ROC) analysis was carried out to identify the cut-off value of the ECS.

Results: Three quality of care (QoC) profiles were identified: high QoC (24.41%), moderate QoC (44.60%) and low QoC (30.99%). Worse affordability of living expenses, insufficient social support, and higher perceived overload were associated with low QoC. Better quality of pre-illness relationship and superior activities of daily living (ADL) of PwD were associated with high QoC. ROC curve for the ECS identified a cut-off point of 15, demonstrating high sensitivity (0.993) and specificity (0.955).

Conclusions: The quality of informal care has obvious heterogeneity and classification characteristics. Understanding the factors influencing of different quality of informal care and the cut-off point provides a basis for precise interventions.

Keywords: Informal caregivers, Patients with dementia, Latent profile analysis, Quality of informal care, Extended stress-process model

1. Introduction

Dementia is a syndrome centered on acquired cognitive impairment that results in a marked decline in the patient's ability to perform daily living, learning, working and social interactions [1]. The increasing number of patients with dementia (PwD) is expected to reach 152 million in 2050 [2]. In China , the number of PwD accounts for about a quarter of the total number, ranking first in the world [3,4]. Most of PwD live at home and are taken care of by informal caregivers (CGs) such as their spouses and children [3]. Informal caregivers are thought to be more familiar with their relative with dementia than formal caregivers, and often provide care that is more in line with the relative's preferences and values. However, the sustained provision of care can lead to notable stress, substantial burdens, and a range of physical and mental health issues for caregivers [5], ultimately impacting the quality of care (QoC) provided [6,7]. Some studies have found low QoC impacts negatively on the health of PwD, increasing the risk of recurrent hospitalizations and institutionalization, indirectly shortening survival [8,9]. Consequently, it is essential to identify individuals with suboptimal quality of informal care early and implement targeted interventions.

QoC is recognized as a multidimensional construct [10], encompassing caregivers' behaviors that may negatively impact care recipients (CRs) well-being, the adequacy of caregiving behaviors, and the sensitivity to psychological needs for respect and pleasant activities. Traditionally, QoC focuses on caregivers' behaviors that negatively impact the well-being of CRs and assessing the extent to which CRs' daily basic and instrumental daily needs are met [10]. These dimensions ignored CRs' feelings and wishes, and failing to reflect the meaning of quality home care. Based on

the extensive review and summary of relevant studies, Dooley et al. proposed the concept of exemplary care [11]. It refers to care that caregivers not only meet the basic needs of CRs, but also respect CRs' feelings, preference, opinions, and values, and avoiding defamation or criticism of CR. Exemplary care is fundamentally characterized by sensitivity and respect demonstrated throughout the caregiving process. This approach conveys to CRs the affirming message that they are "loved, respected, and worthy of special attention". The message helps maintain the dignity and integrity of CRs, reflecting the concept of "person-centered" care. Built upon the conceptual framework of "exemplary care", Dooley and colleagues developed the Exemplary Care Scale (ECS) to evaluate the performance of informal caregivers in home-based dementia care [11], containing two core domains: care provision and respect demonstration. In comparison to other commonly used instruments for informal care quality assessment, such as the comprehensive yet lengthy QUALCARE Scale [12], the functionally-oriented Family Caregiving Consequences Inventory (FCCI) [13], and the negatively-focused Potential Harmful Behaviors scale (PHB) [14,15], the ECS stands out with its unique positive perspective on caregiving. Its cross-cultural validity has been confirmed through rigorous validation studies conducted in diverse populations, including the United States [16], Argentina [17], and China [18], making it a particularly reliable and practical tool for assessing QoC for dementia patients in Chinese household settings.

Many researches mainly focus on the total score of ECS scale to assess overall QoC and its influencing factors [16-20]. However, these studies adopt a

variable-oriented approach, which fails to capture individual differences and obscures heterogeneity among different subgroups. It is essential to identify distinct patterns of the QoC within the group and to implement targeted interventions based on their different characteristics. Although existing research has identified distinct caregiver profiles associated with different types of low QoC in the elderly [21], few studies have examined characteristic within groups of caregivers of PwD, resulting in a lack of empirical basis for precise support for high-risk caregivers of PwD supplied low QoC. Latent profile analysis (LPA) is an individual-centered statistical analysis method, identifying group heterogeneity through the response patterns of continuous variables and dividing individuals into potentially homogeneous subgroups [22]. Building upon the subgroup classification derived from LPA, this study subsequently employs logistic regression to examine influencing factors for each subgroup, guided by McClendon's Extended Stress Process Model [16]. The model takes "QoC" as the core outcome variable, shifting the research perspective from "caregiver-centered" to the "caregiver-patient dyadic integration." Including multidimensional influences provides a systematic theoretical framework for understanding QoC in dementia. The results of this study will provide important empirical evidence for developing precise intervention strategies for different caregiver subgroups.

Although heterogeneity analysis can reveal differences among different groups, there are limitations in its application for rapid clinical screening of high-risk groups. To address this practical challenge, this study further investigates the optimal cutoff

value of ECS employing ROC analysis, with LPA classification results serving as the criteria. This approach allows clinical and community workers to rapidly identify high-risk groups and intervene promptly, based solely on the total score. This methodological innovation preserves LPA's advantage to identify heterogeneity and enhances its clinical applicability by simplifying the scoring process.

Therefore, the objectives of this study were threefold: (1) to identify potential heterogeneous subgroups using LPA, thereby overcoming the limitations of traditional total score analysis, which assumes that "the same score can represent different qualities"; (2) to clarify the influencing factors of these subgroups through logistic regression analysis, providing empirical evidence to understand the causes of heterogeneity; and (3) to identify simplified categorization thresholds using ROC analysis, facilitating the transition from theoretical findings to clinical applications. These findings hold dual practical significance. Firstly, they provide a scientific basis for developing precise interventions tailored to different subgroups. Secondly, by establishing rapid screening criteria, they enable healthcare organizations to promptly identify high-risk individuals, significantly enhancing the efficiency and relevance of quality of care assessment. Our hypotheses are: (1) quality of informal care can be categorized into distinct latent profiles based on ECS; (2) the quality of informal care provided by caregivers varies due to individual characteristics, stressors, and social support. (3) ROC analysis will be employed to determine an optimal cut-off value with high sensitivity and specificity for identifying low-quality care.

2. Materials and Methods

2.1 Design and participants

This study used a cross-sectional design. From July 15, 2023, to 14 July, 2024, a non-probability convenience sampling method was used to select PwD and their informal caregivers in memory clinic, rehabilitation clinic, and neurological clinic in the hospital and communities. PwD were eligible to be included in this study if they≥60 years, met the WHO International Classification of Diseases, 10th Revision (ICD-10) diagnostic criteria for dementia and lived at home with their family before admission (≥3 months) rather than nursing homes [23]. All caregivers were the primary informal caregivers of PwD who did not get paid for caregiving and met the following conditions: (1))≥18 years, (2) spending at least four hours per day on caregiving for no less than three months; (3) often accompanying patients to see a doctor, the best understanding of the patient's condition, and basic living conditions; and (4) clear consciousness, normal intelligence, good communication skills and voluntary participation in this study. Informal caregivers were excluded if they had: other major stressful events such as bereavement and divorce within the past three months. Paper questionnaires were collected during face-to-face interviews with caregivers of PwD. After being thoroughly informed of the purpose of the study, all participants provided written informed consent and completed independent questionnaires anonymously. If the respondents had reading difficulties, questions and answers completed with the assistance of skilled and trained team members of this research. The questionnaires were distributed and collected immediately. After

collecting questionnaires, the investigators immediately checked the questionnaires for missing or obvious logical errors. All problems were resolved immediately. Each questionnaire was completed within 15-25 minutes. For multinomial logistic regression, the sample size should be at least 5-10 times the number of independent variables [24]. With 20 variables included in this study and accounting for a potential 20% rate of invalid questionnaires, a minimum of 120 participants was initially required. To enhance the accuracy and validation efficacy of the potential category model, a sample size of at least 200 was deemed necessary [25]. Initially, 248 participants were recruited. However, 35 were excluded due to incomplete responses or other reasons, resulting in 213 valid participants included in the final analysis (85.89% response rate). The study was approved by the Ethics Committee of TianYou Hospital Affiliated to Wuhan University of Science &Technology (LL-2023-07-14-002).

2.2 Variables and measures

2.2.1 Dependent variable

Quality of care (QoC): The QoC was measured using the Exemplary Care Scale (ECS) [18], which was translated into Chinese by Lau, et al. ECS is completed by informal caregivers and contains two dimensions, Provide (items 1–5) and Respect (items 6–11), for a total of 11 items. Each item is rated on a 4-point scale (0= never, 1= sometimes, 2= often, and 3= always). The total score ranges from 0 to 33. A higher total score indicates a better QoC provided by informal caregivers. Cronbach's alpha

for the entire scale was 0.816.

2.2.2 Independent variable

Basic information of PwD included age, gender, education, type of dementia, years of illness, numbers of children, and residence. Informal caregivers included age, gender, education, relationship with PwD, whether they lived together, length of care, and affordability of living expenses.

Quality of pre-illness relationship: Relationship Rewards Scale (RRS) is used to assess the quality of the pre-illness relationship between caregiver and CR [15]. caregivers assessed the frequency (1= never, 4= always) at which they perceived pre-illness interpersonal relationships with CRs as rewarding, with a higher score indicating a greater quality of pre-illness relationship. The RRS was translated into Chinese using Brislin's guidelines [27]. In this sample of caregivers, Cronbach's alpha was 0.840.

Activities of daily living (ADL): The Barthel Index (BI), a 10-item scale ranging from 0 to 100 points that measures a person's ability to complete basic ADL, was translated into Chinese by Leung et al. in 2007 [28]. In this study, PwD's ADL was classified as no (91–100), mild (61–90), moderate (21–60), and severe disability (0–20) [29]. The scale was completed by informal caregivers based on the condition of PwD.

Perceived Overload: The 4-item Overload scale was used to evaluate caregivers' perceived overload. Items were rated on a 4-point scale ranging from 1 (*Not at all*) to 4 (*Completely*). Scores range from 4 to 16, with higher scores implying higher levels of perceived overload [26]. The Chinese version of the Overload scale was produced

following Brislin's guidelines [27]. The Cronbach's alpha of the Overload scale was 0.791 in this study.

Physical health: The caregiver's physical health using self-rated health with one general question: "How do you rate your general health?" Responses included "very poor", "poor", "fair", "good", and "very good". For data analysis, we treated the original responses as a binary variable of poor (1= very poor, poor, fair) and good (2= good, very good).

Depression: Caregivers' depression was assessed using the Center for Epidemiologic Studies Depression Scale (CES-D) developed by Radloff [30]. The 20-item CES-D scale asks respondents how often they had experienced depressive symptoms in the past week. Scores of ≥16 indicated the presence of clinically significant depression and <16 was classified as no depression [31]. The Cronbach's alpha for the CES-D was 0.877 in this study.

Social support: Social support was evaluated using the 10-item the Social Support Rating Scale (SSRS), which was designed for the Chinese population by Xiao, based on the Social Support Questionnaire (SSQ) and Interview Schedule for Social Interaction (ISSI) [32,33]. The SSRS includes subjective support, objective support, and support utilization. Total score ranges from 12 to 66 [34]. Social support can be divided into three levels: low-level support (≤22), medium-level support (23-44), and high-level support (≥45). In this study, the Cronbach's alpha for the SSRS was 0.804.

2.3 Statistics analysis

SPSS 26.0 was used for the descriptive statistics of all variables to understand the basic characteristics of PwD and their informal caregivers. Each item score on the ECS serves as explicit indicators to establish a LPA model using Mplus 8.3. The most suitable model was selected based on fit indices [35], including the Akaike Information Criterion (AIC), Bayesian Information Criterion (BIC), and adjusted Bayesian Information Criterion (aBIC), Lo-Mendell-Rubin likelihood ratio test (LMR), Bootstrapped likelihood ratio test (BLRT), and Entropy. Lower values of AIC, BIC, and aBIC indicated a superior model fit. Differences between latent profile models were compared using the LMR and BLRT. A significant *P*-value suggests that the model with *k* classes fits the data better than the model with *k*-1 classes. Entropy was used to assess classification accuracy, with values closer to 1 indicating more precise classification; an entropy value ≥0.8 indicates a classification accuracy exceeding 90%. Cohen's d was calculated to further validate the accuracy of the classification (0.2-0.5: small; 0.5-0.8: medium; >0.8: large) [36,37]. To examine the effects of predictor variables on latent profile of QoC, we employed the R3STEP command in Mplus, which effectively controlling for potential effects arises from classification errors [38]. Receiver operating characteristic (ROC) analysis was conducted to determine the optimal cut-off value for the ECS using SAS 9.4. The optimal cut-off point was defined as the value that maximized Youden's index, which maximizes the sum of sensitivity and specificity. Youden's index ranges from 0 to 1, with higher values indicating better classification performance [39]. The area under the curve (AUC) was computed to evaluate the accuracy of identifying low QoC.

AUC values were interpreted as follows: ⩾0.90 (excellent), 0.80-0.89 (good), 0.70-0.79 (fair), and <0.70 (poor). The *P*-value <0.05 indicated statistical significance.

3. Results

3.1 Sample characteristics

A total of 213 dyads of PwD and their informal caregivers were recruited for the study. The average age of PwD was 77.29±9.42, mainly cared for by their spouse or children. Caregivers was predominantly were female (64.8%). 75.6% caregivers lived with PwD. Other information is shown in Table 1.

Table 1 Characteristics of PwD and their informal caregivers (N=213)

PwD	M±SD or n (%)	Informal caregivers	M±SD or n (%)
Age(years)	77.29±9.42	Age(years)	59.06±14.33
Gender		Gender	
Male	117 (54.9)	Male	75 (35.2)
Female	96 (45.1)	Female	138 (64.8)
Education		Education	
Primary school or below	112 (52.6)	Primary school or below	45 (21.1)
Secondary school	87 (40.8)	Secondary school	94 (44.1)
High school or above	14 (6.6)	High school or above	74 (34.8)
Type of dementia		Relationship with PwD	
Alzheimer	128 (60.1)	Spouse	76 (35.7)
Vascular dementia	71 (33.3)	Children	110 (51.6)
Other	14 (6.6)	Other	27 (12.7)
Years of illness		Living with PwD	
～1	58 (27.2)	Yes	161 (75.6)
～5	99 (46.5)	No	52 (24.4)
≥5	56 (26.3)	Length of care (year)	
Numbers of children		～1	70 (32.9)
0	1 (0.5)	～5	97 (45.5)
1	50 (23.5)	≥5	46 (21.6)
2	70 (32.8)	Affordability of living expenses	
≥3	92 (43.2)	Difficult	62 (29.1)

ADL		Somewhat difficult	67 (31.5)
Severe	94 (44.1)	Not difficult	84 (39.4)
Moderate	43 (20.2)	Physical health	
Mild	56 (26.3)	Poor	140 (65.7)
No	20 (9.4)	Good	73 (34.3)
Residence		Depression	
Rural	70 (32.9)	No	144 (67.6)
Urban and town	143 (67.1)	Yes	69 (32.4)
		Quality of pre-illness relationship	11.68±2.82
		Perceived overload	9.63±2.90
		Social support	39.25±7.04

3.2 Latent profile analysis

The fit indices from the 1- to 5-profile QoC among informal caregivers of PwD were shown in Table 2. The AIC, BIC, and aBIC values decreased continuously as the number of profiles gradually increased. Since the *P* values of LMR were >0.05 in the 4-profile model, this model was excluded. The *P* values of LMR and BLRT were significant for the 2-, 3-, and 5-profile models, indicating that the posterior models (3- and 5-profile) were better than the previous model. Although the entropy of the 5-profile model was higher than that of the 3-profile model, and the AIC, BIC, and aBIC values were lower than that of the 3-profile, more profiles indicated that the effective information was more dispersed. Therefore, we chose the 3-profile model as the final fitting model. The average probability of QoC belonging to the profile ranged from 92.0% to 95.2% (see Table 3), and the Cohen's d values of the three-profile model were both higher than 0.80, indicating that the results of the 3-profile models were reasonable. The details are shown in Table 4.

Table 2 Indicators for each latent profile of QoC

profile	Likelihood	AIC	BIC	aBIC	LMR(*P*)	BLRT(*P*)	Entropy	Proportion

1	-2596.885	5237.769	5311.718	5242.006	-	-	-	-
2	-2370.772	4809.544	4923.828	4816.092	0.000	0.000	0.892	0.338/0.662
**3**	**-2289.612**	**4671.224**	**4825.844**	**4680.083**	**0.016**	**0.000**	**0.855**	**0.244/0.446/0.310**
4	-2250.044	4616.088	4811.043	4627.258	0.517	0.000	0.851	0.300/0.174/0.235/0.291
5	-2017.855	4283.771	4519.061	4297.251	0.017	0.000	0.956	0.146/0.150/0.113/0.221/0.371

Table 3 Average probability of attribution for each potential profile

Class	Profile 1	Profile 2	Profile 3
Profile 1	0.952	0.000	0.048
Profile 2	0.000	0.920	0.080
Profile 3	0.028	0.046	0.927

Table 4 Descriptive information of the ECS total score for each profile from LPA

	M(SD)	N(%)	Score Ranges	Cohen's d
LPA[a]				
Low QoC	11.35 (2.10)	66 (30.99%)	[5,16]	d_{2-1}=3.24
Moderate QoC	18.13 (2.09)	95 (44.60%)	[12,22]	d_{3-2}=2.06
High QoC	22.73 (2.51)	52 (24.41%)	[19,29]	d_{3-1}=4.97

Cohen's $d_{2–1}$: the standardized mean difference between moderate-level QoC and low-level QoC; Cohen's $d_{3–1}$: the standardized mean difference between high-level QoC and low-level QoC; Cohen's $d_{3–2}$: the standardized mean difference between high-level QoC and moderate-level QoC.

The three potential profiles of QoC among informal caregivers of PwD scored differently on the eleven items and exhibited distinct characteristics. According to the scoring characteristics, Profile 1 had the highest average score in each item, so named high QoC, accounting for 24.4% of the total. Profile 2 had scores for all items in a middle range, hence named moderate QoC, accounting for 44.6%. Profile 3 scores for each item were much lower than those of Profiles 1 and 2, named low QoC, accounting for 31.0%. The scores of the three profiles on the eleven item of QoC are depicted in Fig.1.

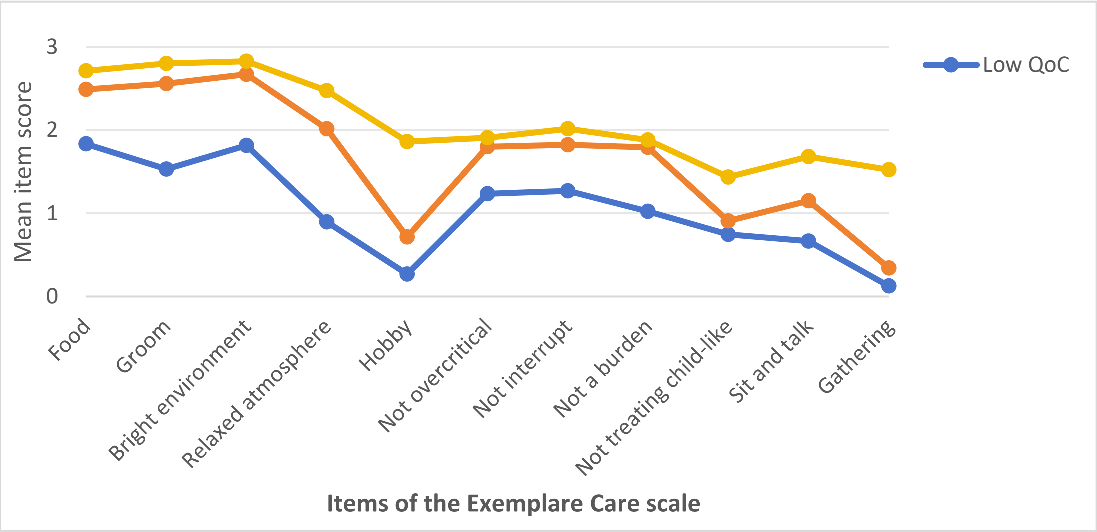

Fig. 1. Latent profile model of QoC among informal caregivers of PwD

3.3 Influencing factors analysis

Utilizing the final potential profile, this study employed a multinomial logistic regression based on the R3STEP procedure to examine the impact on three potential profile. Table 4 displays the outcomes of the logistic regression model with "moderate QoC" serving as the reference category.

The results in Table 5 showed that worse affordability of living expenses (β= -1.420, P<0.05), insufficient social support (β= -0.201, P<0.01), and higher perceived overload (β=0.344, P<0.01) was associated with low QoC group; better quality of pre-illness relationship (β=0.315, P<0.01) and PwD's superior ADL performance (β=1.082, P<0.001) were associated with high QoC group.

Table 5 Test of categorical latent variable multinomial logistic regressions using the 3-step procedure

Variable	low VS moderate (ref)		high VS moderate(ref)	
	β	SE	β	SE
**background variables**				
Affordability of living expenses	-1.420*	0.673	-0.200	0.485
caregiver Quality of pre-illness relationship	-0.251	0.130	0.315**	0.118

**stressors**				
PwD ADL	-0.063	0.380	1.082***	0.291
caregiver Perceived overload	0.344**	0.132	0.064	0.137
**caregiver resource**				
caregiver Social support	-0.201**	0.064	0.068	0.057

Note: $^{*}P < 0.05$; $^{**}P < 0.01$; $^{***}P < 0.001$

3.4 ROC analysis results

Participants in the "high QoC" and "moderate QoC" profile were coded as "cases" while those in the "low QoC" profiles were coded as "non-cases". Using this reference standard, the optimal cut-off point was 15, resulting in the highest Youden's index of 0.948 (see Table 6). This cut-off point had high sensitivity (0.993) and specificity (0.955). Additionally, Fig. 2 showed an AUC of 0.995 (95 % CI [0.988, 0.999]; $P < 0.001$), further supporting this cut-off point. The mean differences between the two groups were statistically significant, with a large effect size (see Table 7).

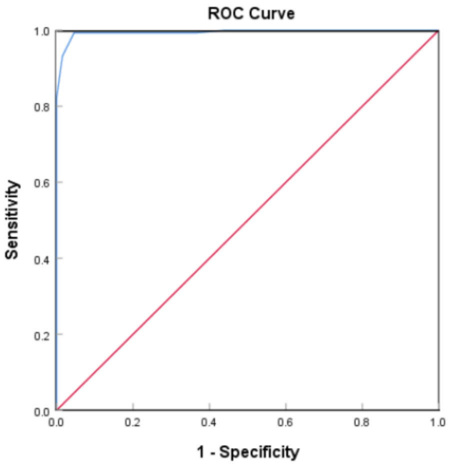

Fig. 2. ROC curve for the classification of moderate and high QoC.

Table 6 Cut-off point based on the "moderate QoC" and "high QoC" group derived through LPA

Cut-off point	Sensitivity	Specificity	Youden's index
13	0.993	0.636	0.629
14	0.993	0.848	0.841
15	0.993	0.955	0.948
16	0.932	0.985	0.917

Note: Estimates in italic typeface are the suggested optimal cut-off point.

Table 7 Descriptive information of the positive/negative group from ROC analysis.

	M(SD)	N(%)	Score Ranges	Cohen's d
ROC				
Negative group	11.35 (2.10)	66 (30.99%)	[5,16]	d=2.93
Positive group	19.76 (3.14)	147 (69.01%)	[12,29]	

4. Discussion

Using LPA to classify subgroups of QoC among caregivers of PwD, the results showed QoC has explicit classification characteristics. Based on Extended Stress Model, this study examined how background variables, primary stressors, caregivers' quality of life, and caregiver resources predicted different profiles of quality of informal care. Overall, lower cost of living affordability, higher perceived overload and inadequate social support were related to the low QoC group; better quality of the pre-illness relationship and superior ADL performance of PwD were linked to the high QoC group. The cut-off point of the ECS was determined at 15 using ROC analysis, providing an initial reference standard for future screening and intervention studies.

We used LPA to categorize the caregivers of PwD into three distinct profiles, i.e., high QoC (24.41%), moderate QoC (44.60%), and low QoC (30.99%). Statistically significant differences were found between the high and moderate QoC profile in terms of pre-illness relationship quality and PwD' ADL. Logistic regression analysis revealed that the high QoC profile was linked to great quality of pre-illness relationship and superior PwD' ADL. Similarly, significant differences in economic status, perceived overload, and social support were observed between the low and moderate QoC profile. The low QoC profile was associated with low economic

affordability, high perceived overload, and inadequate social support. These results highlight that tailored strategies based on the distinct characteristics of each caregiver subgroup. Priority should be given to caregivers of PwD facing financial strain, high perceived overload, and insufficient social support.

The Extended Stress Process Model hints that background variables, primary stressors, quality of life, and caregiver resources collectively influence the quality of informal care. Our study verified that background variables, primary stressors, and caregiver resources impacted the quality of informal care, except caregivers' quality of life. Specifically, affordability of living expenses, pre-illness relationship quality, the ADL of PwD, perceived overload, and social support decided three QoC profiles. These findings provide an innovative perspective on the importance of economic status, relationship quality, caregiving stress, social support in mitigating the deterioration of QoC among caregivers of PwD.

Family affordability of living expense was a crucial factor for QoC, resonating with other researches [40,41]. Caregivers with higher family economic levels are more likely to provide more resources during the treatment and rehabilitation process, which helps improve the QoC and patient prognosis. Align with previous studies [15,42], a positive pre-illness relationship serves as a protective factor for QoC. As Steadman et al. demonstrated [43], caregivers reporting higher premorbid relationship satisfaction showed significantly less stress and less reactivity to memory and behavioral problems, and better problem solving skills and more effective communication. Conversely, caregivers with poor pre-illness relationships may

perceive caregiving responsibilities as an additional burden, leading to negative attitudes and behaviors [41,43], ultimately compromising the quality of informal care. The finding emphasizes the critical role of caregiving context during care, suggesting that interventions aimed at improving QoC should consider pre-illness relationship quality.

PwD's ADL is positively correlated with the QoC, whereas caregivers overload shows a negative correlation, a conclusion supported by extensive research [7,40,41,42,44]. Caregivers experience tremendous stress and high levels of overload in the long-term care of the elderly PwD [45], while juggling familial, occupational, and caregiving responsibilities. Long-term high-intensity caregiving deteriorates caregivers' mental health and caregivers' ability to provide care, further diminishing the QoC [46,47]. The decline in ADL among PwD significantly increases caregiver overload [48]. Research has shown that cognitive impairment or psychiatric symptoms are prevalent in most patients at the time of diagnosis [3], substantially compromising their daily functioning and necessitating caregiver assistance. However, many caregivers of PwD struggle to provide consistent and attentive care due to insufficient care capacity and a lack of care resources, ultimately resulting in diminished quality of care [49]. Furthermore, disability is often accompanied by aphasia and comprehension deficits, making it difficult for patients to express their needs accurately. These challenges may undermine caregivers' patience and lead to inadequate care. As the PwD's ADL gradually decrease, the level of QoC may be dropped from high to moderate or even low. Thus, timely assessment of ADL are

critical to improve the QoC.

Adequate social support helps preventing low QOC, consisting other studies [44,50]. The buffering model of social support theory suggests that social support acts as a protective factor, reducing the adverse impact of stressful events on individuals [51]. However, the current caregiving system in China remains underdeveloped [52], with caregivers predominantly relying on informal family support networks, while formal support resources at both community and national levels are insufficient [53]. This unidimensional and incomplete support system highlights the establishment of a diversified social support network as a crucial intervention target for improving care quality. Moving forward, it is imperative to strengthen community- and national-level support to provide caregivers with more resources and assistance, collectively building a more comprehensive caregiving system.

Notably, unlike other studies [7,47], this research did not find that caregiver depression affects the QoC provided by informal caregivers, which may be related to the issue of mental health stigma. In China, caregivers may be reluctant to acknowledge their depressive feelings, or societal expectations may pressure them to conceal these emotions [54]. This phenomenon may lead to an underestimation of depression cases in research, resulting in an incomplete reflection of its true impact. Therefore, future studies should further investigate the relationship between caregiver depression and the quality of informal care provided by informal caregivers in the context of Chinese cultural scene, ultimately developing a culturally adapted QoC assessment model.

Combining LPA and ROC analysis, the study revealed that a cut-off point of 15 had the highest sensitivity and specificity for identifying individuals with low quality of informal care, which may provide guidance for future research on the quality of informal care for PwD. The determination of the cut-off value is of great significance for identifying those with low QoC for PwD. Public health professionals are suggested to collect information on the quality of informal care for PwD, implement corresponding interventions for family caregivers of PwD whose scores are less than 15, and provide necessary support. This study deepened our understanding of the quality of informal care for PwD and provided feasible suggestions for public health professionals to improve quality of informal care for PwD in the future.

4.4 Limitations

First, due to the cross-sectional design, causal relationships cannot be established. Future longitudinal studies are needed to strengthen the conclusions. Second, the study only included informal caregivers of PwD in one city in China, which may restrict the generalizability of the findings. Future research should expand the sample to caregivers from diverse regions and urbanization levels to enhance external validity. Third, although this study reported high sensitivity and specificity, the ECS cut-off value of 15 was determined through ROC analysis. Future validation in different samples are needed to enhance the clinical applicability of this threshold. Finally, the self-reported ECS may be influenced by social desirability bias and subjective reporting errors. Future studies could incorporate objective measures (e.g., clinical

assessments, or behavioral observations) to improve data reliability.

5. Conclusion

In this study, we used the concept of "exemplary care" to assess the QoC provided by informal caregivers for PwD in terms of the Provide dimension and the Respect dimension. Using LPA techniques, low, moderate, and high QoC profiles were identified, indicating the heterogeneity of the quality of informal care provided by caregivers for PwD. Based on McClendon's Extended Stress Process Model, we found that worse cost-of-living affordability, inadequate social support and high perceived overload significantly predicted low QoC; better pre-morbid relationships and superior ADL of PwD were predictors of high QoC. Public health professionals should comprehensively consider these factors. Targeted interventions should be applied simultaneously to homogeneous informal caregivers to improve the quality of informal care and prevent a decline in quality of informal care. Furthermore, this study identified the precise critical value of ECS, providing a valuable tool for screening low QoC in PwD and aiding in the identification of PwD and caregivers who may benefit from tailored intervention measures.

Abbreviations

QoC: quality of care; PwD: patients with dementia; ECS: Exemplary Care Scale; RRS: Relationship Rewards Scale; BI: The Barthel Index; CES-D: Epidemiologic Studies Depression Scale; SSRS: Social Support Rating Scale; LPA: Latent profile analysis;

ADL: activity of daily living; CRs: care recipients; CGs: caregivers; ref: reference; CI: confidence interval.

Ethical Approval

The studies involving humans were approved by Ethics Committee of TianYou Hospital Affiliated to Wuhan University of Science &Technology, Wuhan 430064, China. The studies were conducted in accordance with the local legislation and institutional requirements (LL-2023-07-14-002). The participants provided their written informed consent to participate in this study.

Data Availability statement

The data used or analyzed in this study may be obtained from the corresponding author upon reasonable request.

Funding

This research received no specific grant from any funding agency in the public, commercial, or not-for-profit sectors.

Declaration of interest statement

The authors declare that they have no conflict of interest.

Acknowledgments

We are grateful to all the people who agreed to participate in this study for their support, time, and patience. We also thank all staff in the hospital and community centers for their patience and cooperation.

References

[1] 2018 Chinese Guidelines for the Diagnosis and Treatment of Dementia and Cognitive Impairment (Part 1): Dementia and Its Classification Diagnostic Criteria [J] Chinese Medical Journal. 2018,98 (13): 965-970. DOI: 10.3760/cma.j.issn.0376-2491.2018.13.003

[2] Livingston G, Huntley J, Sommerlad A, et al. Dementia prevention, intervention, and care: 2020 report of the Lancet Commission. Lancet. 2020;396(10248):413-446. doi:10.1016/S0140-6736(20)30367-6.

[3] Jia L, Quan M, Fu Y, et al. Dementia in China: epidemiology, clinical management, and research advances. Lancet Neurol. 2020;19(1):81-92. doi:10.1016/S1474-4422(19)30290-X.

[4] Jia L, Du Y, Chu L, et al. Prevalence, risk factors, and management of dementia and mild cognitive impairment in adults aged 60 years or older in China: a cross-sectional study. Lancet Public Health. 2020;5(12):e661-e671. doi:10.1016/S2468-2667(20)30185-7.

[5] Parker LJ, Fabius C, Rivers E, Taylor JL. Is Dementia-Specific Caregiving Compared With Non-Dementia Caregiving Associated With Physical Difficulty Among Caregivers for Community-Dwelling Adults?. J Appl Gerontol. 2022;41(4):1074-1080. doi:10.1177/07334648211014352

[6] Wang M, Sun H, Zhang J, Ruan J. Prevalence and associated factors of elder abuse in family caregivers of older people with dementia in central China cross-sectional study. Int J Geriatr Psychiatry. 2019;34(2):299-307. doi:10.1002/gps.5020

[7] Beach, S. R., Schulz, R., Williamson, G. M., et al. (2005). Risk factors for potentially harmful informal caregiver behavior. Journal of the American Geriatrics Society, 53(2), 255–261. https://doi.org/10.1111/j.1532-5415.2005.53111.x

[8] McClendon, M. J., & Smyth, K. A. (2015). Quality of in-home care, long-term care placement, and the survival of persons with dementia. Aging & mental health, 19(12), 1093–1102. https://doi.org/10.1080/13607863.2014.1003284.

[9] Josephson S. A. (2016). Focusing on transitions of care: A change is here. Neurology. Clinical practice, 6(2), 183–189. https://doi.org/10.1212/CPJ.0000000000000207.

[10] Christie, J., Smith, G. R., Williamson, G. M., et al. (2009). Quality of informal care is multidimensional. Rehabilitation psychology, 54(2), 173–181. https://doi.org/10.1037/a0015705.

[11] Dooley, W. K., Shaffer, D. R., Lance, C. E., & Williamson, G. M. (2007). Informal care can be better than adequate: Development and evaluation of the Exemplary Care Scale. Rehabilitation Psychology, 52(4), 359–369. https://doi.org/10.1037/0090-5550.52.4.359.

[12] Phillips LR, Morrison EF, Chae YM. The QUALCARE Scale: developing an instrument to measure quality of home care. Int J Nurs Stud. 1990;27(1):61-75. doi:10.1016/0020-7489(90)90024-d

[13] Shyu YI, Lee HC, Chen ML. Development and testing of the Family Caregiving

Consequences Inventory for home nursing assessment in Taiwan. J Adv Nurs. 1999;30(3):646-654. doi:10.1046/j.1365-2648.1999.01136.x

[14] Sasaki M, Arai Y, Kumamoto K, Abe K, Arai A, Mizuno Y. Factors related to potentially harmful behaviors towards disabled older people by family caregivers in Japan. Int J Geriatr Psychiatry. 2007;22(3):250-257. doi:10.1002/gps.1670.

[15] Williamson GM, Shaffer DR. Relationship quality and potentially harmful behaviors by spousal caregivers: how we were then, how we are now. The Family Relationships in Late Life Project. Psychol Aging. 2001;16(2):217-226.

[16] McClendon, M. J., & Smyth, K. A. (2013). Quality of informal care for persons with dementia: dimensions and correlates. Aging & mental health, 17(8), 1003 – 1015. https://doi.org/10.1080/13607863.2013.805400.

[17] Morlett Paredes A, Perrin PB, Peralta SV, et al. Structural equation model linking dementia cognitive functioning, caregiver mental health, burden, and quality of informal care in Argentina. Dementia (London). 2017;16(6):766-779. doi:10.1177/1471301215617080

[18] Lau, B. H. P., Lou, V. W., & Cheung, K. S. L. (2018). Exemplary Care among Chinese Dementia Familial Caregivers. Healthcare (Basel, Switzerland), 6(4), 141. https://doi.org/10.3390/healthcare6040141

[19] Panyavin I, Trujillo MA, Peralta SV, et al. Examining the influence of family dynamics on quality of care by informal caregivers of patients with Alzheimer's dementia in Argentina. Am J Alzheimers Dis Other Demen. 2015;30(6):613-621. doi:10.1177/1533317515577129

[20] Harris GM, Durkin DW, Allen RS, DeCoster J, Burgio LD. Exemplary care as a mediator of the effects of caregiver subjective appraisal and emotional outcomes. Gerontologist. 2011;51(3):332-342. doi:10.1093/geront/gnr003

[21] Yan E, Lai DWL, Sun R, et al. Typology of family caregivers of older persons: A latent profile analysis using elder mistreatment risk and protective factors. J Elder Abuse Negl. 2023;35(1):34-64. doi:10.1080/08946566.2023.2197269

[22] Collins LM, Lanza ST. Latent Class and Latent Transition Analysis: With Applications in the Social, Behavioral, and Health Sciences. USA: John Wiley & Sons; 2009.

[23] Organization WH. The ICD-10 Classification of Mental and Behavioral Disorders. Diagnostic Criteria for Research. Geneva: Organization WH; 1993.

[24] Xu, Y., Chen, Z., Tang, X. et al. Latent profile analysis of nutrition knowledge, attitudes, and practices and their influencing factors in maintenance hemodialysis patients. Sci Rep 15, 17246 (2025). https://doi.org/10.1038/s41598-025-02142-4.

[25] Chen X, Wang Z, Wang S, et al. Latent profiles of ambivalence over emotional expression in young breast cancer patients: A cross-sectional study. Int J Nurs Sci. 2025;12(4):379-385. Published 2025 Jun 16. doi:10.1016/j.ijnss.2025.06.005.

[26] Pearlin, L. I., Mullan, J. T., Semple, S. J., & Skaff, M. M. (1990). Caregiving and the stress process: an overview of concepts and their measures. The Gerontologist, 30(5), 583 – 594. https://doi.org/10.1093/geront/30.5.583.

[27] Brislin, R.W. (1980) Translation and content analysis of oral and written material. In: Triandis, H.C. and Berry, J. W., Eds., Handbook of cross-cultural psychology: Methodology, Allyn and Bacon, Boston, 389-444.

[28] Leung, S. O., Chan, C. C., & Shah, S. (2007). Development of a Chinese version of the Modified Barthel Index-- validity and reliability. Clinical rehabilitation, 21(10), 912 – 922.

https://doi.org/10.1177/0269215507077286.
[29] Shah, S., Vanclay, F., & Cooper, B. (1989). Improving the sensitivity of the Barthel Index for stroke rehabilitation. Journal of clinical epidemiology, 42(8), 703 – 709. https://doi.org/10.1016/0895-4356(89)90065-6.
[30] Radloff, L. S. (1977). The CES-D Scale: A Self-Report Depression Scale for Research in the General Population. Applied Psychological Measurement, 1(3), 385-401. https://doi.org/10.1177/014662167700100306.
[31] Thompson T, Rodebaugh TL, Haun P, et al. Longitudinal Dyadic Analysis of Psychosocial and Health Outcomes in Black Breast Cancer Patients and Their Informal Caregivers. Cancer Control. 2025;32:10732748251362950. doi:10.1177/10732748251362950
[32] Sarason, I. G., Johnson, J. H. (1981). A technical report. Washington D. C. : University of Washington.
[33] Henderson, S., Byrne, D.G., Duncan-Jones, P. (1982). Neurosis and the Social Environment.
[34] Xiao, S. Y. The theoretical basis and research application of Social Support Rating Scale. Journal of Clinical Psychological Medicine. 1994,2, 98 – 100. (in Chinese)
[35] Kim, S. Y. (2014). Determining the Number of Latent Classes in Single- and Multiphase Growth Mixture Models. Structural Equation Modeling: A Multidisciplinary Journal, 21(2), 263 – 279. https://doi.org/10.1080/10705511.2014.882690.
[36] Cohen, J. (1988). Statistical power analysis for the behavioral sciences (2nd ed.). New Jersey: Lawrence Erlbaum.
[37] Luo, J., Bei, D., Gong, J., & Wang, M. (2024). Classification of nomophobia among Chinese college students: Evidence from latent profile and ROC analysis. Journal of Behavioral Addictions, 13(2), 482-494. https://doi.org/10.1556/2006.2024.00013
[38] Gabriel AS, Daniels MA, Diefendorff JM, Greguras GJ. Emotional labor actors: a latent profile analysis of emotional labor strategies. J Appl Psychol. 2015;100(3):863-879. doi:10.1037/a0037408.
[39] Nahm FS. Receiver operating characteristic curve: overview and practical use for clinicians. Korean J Anesthesiology. 2022;75(1):25 – 36. https://doi.org/10.4097/kja.21209.
[40] Burnes D, Pillemer K, Caccamise PL, et al. Prevalence of and Risk Factors for Elder Abuse and Neglect in the Community: A Population-Based Study. J Am Geriatr Soc. 2015;63(9):1906-1912. doi:10.1111/jgs.13601.
[41] Fang B, Yan E, Lai DWL. Risk and protective factors associated with domestic abuse among older Chinese in the People's Republic of China. Arch Gerontol Geriatr. 2019;82:120-127. doi:10.1016/j.archger.2019.02.001.
[42] Orfila F, Coma-Solé M, Cabanas M, et al. Family caregiver mistreatment of the elderly: prevalence of risk and associated factors. BMC Public Health. 2018;18(1):167. Published 2018 Jan 22. doi:10.1186/s12889-018-5067-8
[43] Steadman PL, Tremont G, Davis JD. Premorbid relationship satisfaction and caregiver burden in dementia caregivers. J Geriatr Psychiatry Neurol. 2007;20(2):115-119. doi:10.1177/0891988706298624
[44] Huang, W., He, B., Wang, Y. H., Ma, W. J., & Zhou, J. (2020). Disability Severity and Home-Based Care Quality in Older Adults: The Mediating Effects of Social Support and Caregiver Competence. The journal of nursing research : JNR, 28(6), e122. https://doi.org/10.1097/jnr.0000000000000405.

[45] Sheehan, O. C., Haley, W. E., Howard, V. J., et al. (2021). Stress, Burden, and Well-Being in Dementia and Nondementia Caregivers: Insights From the Caregiving Transitions Study. The Gerontologist, 61(5), 670 – 679. https://doi.org/10.1093/geront/gnaa108

[46] Liu xiaohui, Yang yuyan, Xue xijuan, et al. (2019). The correlation between the quality of family care for disabled elderly and caregiver burden. Chinese Journal of Gerontology. 39(16), 4081-4084. DOI:10.3969/j.issn.1005-9202.2019.16.067

[47] Smith, G. R., Williamson, G. M., Miller, L. S., & Schulz, R. (2011). Depression and quality of informal care: a longitudinal investigation of caregiving stressors. Psychology and aging, 26(3), 584–591. https://doi.org/10.1037/a0022263.

[48] Kim, B., Noh, G.O. & Kim, K. Behavioural and psychological symptoms of dementia in patients with Alzheimer’s disease and family caregiver burden: a path analysis. BMC Geriatr 21, 160 (2021). https://doi.org/10.1186/s12877-021-02109-w

[49] Report on the family survival status of Alzheimer's disease patients in China. Alzheimer’s Disease Chinese, 2020-1. 2019. URL: http://weixin.moreedge.cn/bps_2019/index.php

[50] Serra L, Contador I, Fernández-Calvo B, et al. Resilience and social support as protective factors against abuse of patients with dementia: A study on family caregivers. Int J Geriatr Psychiatry. 2018;33(8):1132-1138. doi:10.1002/gps.4905

[51] Cohen S, Wills TA. Stress, social support, and the buffering hypothesis. Psychol Bull. 1985;98(2):310-357

[52] Li J, Gao G, Zeng Y, et al. Perceived social support in Chinese family caregivers of patients with dementia. Int J Nurs Pract. 2022;28(3):e12945. doi:10.1111/ijn.12945

[53] Wu F, Sheng Y. Social support network, social support, self-efficacy, health-promoting behavior and healthy aging among older adults: A pathway analysis. Arch Gerontol Geriatr. 2019;85:103934. doi:10.1016/j.archger.2019.103934

[54] Xu, X., Li, X. M., Zhang, J., & Wang, W. (2017). Mental Health-Related Stigma in China. Issues in Mental Health Nursing, 39(2), 126 – 134. https://doi.org/10.1080/01612840.2017.1368749

Quality of informal care among informal caregivers of patients with dementia: a latent profile and ROC analysis

Chan Cai[a,b,1], Bing Cheng[a,1], Chongqing Shi[a,*], Wenli Shi[a], Chenyang Li[a], Cui Liu[a], Jin Sun[c]

a. Institute of Nursing Research, Hubei Province Key Laboratory of Occupational Hazard Identification and Control, School of Medicine, Wuhan University of Science and Technology, Wuhan 430065, Hubei Province, China.
b. Department of Nursing, Xiangyang No.1 People's Hospital, Hubei University of Medicine, Xiangyang, 441000
c. Yiling Hospital of Yichang City, Yichang, Hubei, China

* Correspondence should be addressed to Chongqing Shi, E-mail: shichongqing@wust.edu.cn; Fax numbers: 0086-27-68893428.

[1]Chan Cai, Bing Cheng have contributed equally to this work.

Email address: caichan718@163.com(C. Cai); 1040178900@qq.com (B. Cheng); shichongqing@wust.edu.cn (C. Shi); wenli_0824@163.com (W. Shi); 1063467525@qq.com (C. Li); 499263863@qq.com (C, Liu); 79817540@qq.com (J Sun).

Abstract

Background: The quality of informal care has become a particularly important issue, as patients with dementia (PwD) often prefer to receive care in home-based, rather than in institutions. However, there is a lack of clinically differentiated intervention programs for populations with different quality of informal care characteristics. Additionally, the absence of clearly thresholds for identifying PwD with low quality of informal care poses a challenge for research and clinical practice. Thus, this study aimed to identify potential profiles of among informal caregivers of PwD, explore influencing factors of different profile, and determine the cut-off point of the Exemplary Care Scale (ECS).

Methods: From 15 July 2023 to 14 July 2024, convenience sampling method was used to select 213 dyads of PwD and their informal caregivers from memory clinic, rehabilitation clinic, and neurological clinic of the tertiary level A hospitals and communities in China. Latent profile analysis (LPA) was used to derive potential profiles. Multinomial logistic regression analysis was conducted to explore influencing factors of quality of informal care in different profiles. Receiver operating characteristic (ROC) analysis was carried out to identify the cut-off value of the ECS.

Results: Three quality of care (QoC) profiles were identified: high QoC (24.41%), moderate QoC (44.60%) and low QoC (30.99%). Worse affordability of living expenses, insufficient social support, and higher perceived overload were associated with low QoC. Better quality of pre-illness relationship and superior activities of daily living (ADL) of PwD were associated with high QoC. ROC curve for the ECS identified a cut-off point of 15, demonstrating high sensitivity (0.993) and specificity (0.955).

Conclusions: The quality of informal care has obvious heterogeneity and classification characteristics. Understanding the factors influencing of different quality of informal care and the cut-off point provides a basis for precise interventions.

Keywords: Informal caregivers, Patients with dementia, Latent profile analysis, Quality of informal care, Extended stress-process model

1. Introduction

Dementia is a syndrome centered on acquired cognitive impairment that results in a marked decline in the patient's ability to perform daily living, learning, working and social interactions [1]. The increasing number of patients with dementia (PwD) is expected to reach 152 million in 2050 [2]. In China , the number of PwD accounts for about a quarter of the total number, ranking first in the world [3,4]. Most of PwD live at home and are taken care of by informal caregivers (CGs) such as their spouses and children [3]. Informal caregivers are thought to be more familiar with their relative with dementia than formal caregivers, and often provide care that is more in line with the relative's preferences and values. However, the sustained provision of care can lead to notable stress, substantial burdens, and a range of physical and mental health issues for caregivers [5], ultimately impacting the quality of care (QoC) provided [6,7]. Some studies have found low QoC impacts negatively on the health of PwD, increasing the risk of recurrent hospitalizations and institutionalization, indirectly shortening survival [8,9]. Consequently, it is essential to identify individuals with suboptimal quality of informal care early and implement targeted interventions.

QoC is recognized as a multidimensional construct [10], encompassing caregivers' behaviors that may negatively impact care recipients (CRs) well-being, the adequacy of caregiving behaviors, and the sensitivity to psychological needs for respect and pleasant activities. Traditionally, QoC focuses on caregivers' behaviors that negatively impact the well-being of CRs and assessing the extent to which CRs' daily basic and instrumental daily needs are met [10]. These dimensions ignored CRs' feelings and wishes, and failing to reflect the meaning of quality home care. Based on

the extensive review and summary of relevant studies, Dooley et al. proposed the concept of exemplary care [11]. It refers to care that caregivers not only meet the basic needs of CRs, but also respect CRs' feelings, preference, opinions, and values, and avoiding defamation or criticism of CR. Exemplary care is fundamentally characterized by sensitivity and respect demonstrated throughout the caregiving process. This approach conveys to CRs the affirming message that they are "loved, respected, and worthy of special attention". The message helps maintain the dignity and integrity of CRs, reflecting the concept of "person-centered" care. Built upon the conceptual framework of "exemplary care", Dooley and colleagues developed the Exemplary Care Scale (ECS) to evaluate the performance of informal caregivers in home-based dementia care [11], containing two core domains: care provision and respect demonstration. In comparison to other commonly used instruments for informal care quality assessment, such as the comprehensive yet lengthy QUALCARE Scale [12], the functionally-oriented Family Caregiving Consequences Inventory (FCCI) [13], and the negatively-focused Potential Harmful Behaviors scale (PHB) [14,15], the ECS stands out with its unique positive perspective on caregiving. Its cross-cultural validity has been confirmed through rigorous validation studies conducted in diverse populations, including the United States [16], Argentina [17], and China [18], making it a particularly reliable and practical tool for assessing QoC for dementia patients in Chinese household settings.

Many researches mainly focus on the total score of ECS scale to assess overall QoC and its influencing factors [16-20]. However, these studies adopt a

variable-oriented approach, which fails to capture individual differences and obscures heterogeneity among different subgroups. It is essential to identify distinct patterns of the QoC within the group and to implement targeted interventions based on their different characteristics. Although existing research has identified distinct caregiver profiles associated with different types of low QoC in the elderly [21], few studies have examined characteristic within groups of caregivers of PwD, resulting in a lack of empirical basis for precise support for high-risk caregivers of PwD supplied low QoC. Latent profile analysis (LPA) is an individual-centered statistical analysis method, identifying group heterogeneity through the response patterns of continuous variables and dividing individuals into potentially homogeneous subgroups [22]. Building upon the subgroup classification derived from LPA, this study subsequently employs logistic regression to examine influencing factors for each subgroup, guided by McClendon's Extended Stress Process Model [16]. The model takes "QoC" as the core outcome variable, shifting the research perspective from "caregiver-centered" to the "caregiver-patient dyadic integration." Including multidimensional influences provides a systematic theoretical framework for understanding QoC in dementia. The results of this study will provide important empirical evidence for developing precise intervention strategies for different caregiver subgroups.

Although heterogeneity analysis can reveal differences among different groups, there are limitations in its application for rapid clinical screening of high-risk groups. To address this practical challenge, this study further investigates the optimal cutoff

value of ECS employing ROC analysis, with LPA classification results serving as the criteria. This approach allows clinical and community workers to rapidly identify high-risk groups and intervene promptly, based solely on the total score. This methodological innovation preserves LPA's advantage to identify heterogeneity and enhances its clinical applicability by simplifying the scoring process.

Therefore, the objectives of this study were threefold: (1) to identify potential heterogeneous subgroups using LPA, thereby overcoming the limitations of traditional total score analysis, which assumes that "the same score can represent different qualities"; (2) to clarify the influencing factors of these subgroups through logistic regression analysis, providing empirical evidence to understand the causes of heterogeneity; and (3) to identify simplified categorization thresholds using ROC analysis, facilitating the transition from theoretical findings to clinical applications. These findings hold dual practical significance. Firstly, they provide a scientific basis for developing precise interventions tailored to different subgroups. Secondly, by establishing rapid screening criteria, they enable healthcare organizations to promptly identify high-risk individuals, significantly enhancing the efficiency and relevance of quality of care assessment. Our hypotheses are: (1) quality of informal care can be categorized into distinct latent profiles based on ECS; (2) the quality of informal care provided by caregivers varies due to individual characteristics, stressors, and social support. (3) ROC analysis will be employed to determine an optimal cut-off value with high sensitivity and specificity for identifying low-quality care.

2. Materials and Methods

2.1 Design and participants

This study used a cross-sectional design. From July 15, 2023, to 14 July, 2024, a non-probability convenience sampling method was used to select PwD and their informal caregivers in memory clinic, rehabilitation clinic, and neurological clinic in the hospital and communities. PwD were eligible to be included in this study if they≥60 years, met the WHO International Classification of Diseases, 10th Revision (ICD-10) diagnostic criteria for dementia and lived at home with their family before admission (≥3 months) rather than nursing homes [23]. All caregivers were the primary informal caregivers of PwD who did not get paid for caregiving and met the following conditions: (1))≥18 years, (2) spending at least four hours per day on caregiving for no less than three months; (3) often accompanying patients to see a doctor, the best understanding of the patient's condition, and basic living conditions; and (4) clear consciousness, normal intelligence, good communication skills and voluntary participation in this study. Informal caregivers were excluded if they had: other major stressful events such as bereavement and divorce within the past three months. Paper questionnaires were collected during face-to-face interviews with caregivers of PwD. After being thoroughly informed of the purpose of the study, all participants provided written informed consent and completed independent questionnaires anonymously. If the respondents had reading difficulties, questions and answers completed with the assistance of skilled and trained team members of this research. The questionnaires were distributed and collected immediately. After

collecting questionnaires, the investigators immediately checked the questionnaires for missing or obvious logical errors. All problems were resolved immediately. Each questionnaire was completed within 15-25 minutes. For multinomial logistic regression, the sample size should be at least 5-10 times the number of independent variables [24]. With 20 variables included in this study and accounting for a potential 20% rate of invalid questionnaires, a minimum of 120 participants was initially required. To enhance the accuracy and validation efficacy of the potential category model, a sample size of at least 200 was deemed necessary [25]. Initially, 248 participants were recruited. However, 35 were excluded due to incomplete responses or other reasons, resulting in 213 valid participants included in the final analysis (85.89% response rate). The study was approved by the Ethics Committee of TianYou Hospital Affiliated to Wuhan University of Science &Technology (LL-2023-07-14-002).

2.2 Variables and measures

2.2.1 Dependent variable

Quality of care (QoC): The QoC was measured using the Exemplary Care Scale (ECS) [18], which was translated into Chinese by Lau, et al. ECS is completed by informal caregivers and contains two dimensions, Provide (items 1–5) and Respect (items 6–11), for a total of 11 items. Each item is rated on a 4-point scale (0= never, 1= sometimes, 2= often, and 3= always). The total score ranges from 0 to 33. A higher total score indicates a better QoC provided by informal caregivers. Cronbach's alpha

for the entire scale was 0.816.

2.2.2 Independent variable

Basic information of PwD included age, gender, education, type of dementia, years of illness, numbers of children, and residence. Informal caregivers included age, gender, education, relationship with PwD, whether they lived together, length of care, and affordability of living expenses.

Quality of pre-illness relationship: Relationship Rewards Scale (RRS) is used to assess the quality of the pre-illness relationship between caregiver and CR [15]. caregivers assessed the frequency (1= never, 4= always) at which they perceived pre-illness interpersonal relationships with CRs as rewarding, with a higher score indicating a greater quality of pre-illness relationship. The RRS was translated into Chinese using Brislin's guidelines [27]. In this sample of caregivers, Cronbach's alpha was 0.840.

Activities of daily living (ADL): The Barthel Index (BI), a 10-item scale ranging from 0 to 100 points that measures a person's ability to complete basic ADL, was translated into Chinese by Leung et al. in 2007 [28]. In this study, PwD's ADL was classified as no (91–100), mild (61–90), moderate (21–60), and severe disability (0–20) [29]. The scale was completed by informal caregivers based on the condition of PwD.

Perceived Overload: The 4-item Overload scale was used to evaluate caregivers' perceived overload. Items were rated on a 4-point scale ranging from 1 (*Not at all*) to 4 (*Completely*). Scores range from 4 to 16, with higher scores implying higher levels of perceived overload [26]. The Chinese version of the Overload scale was produced

following Brislin's guidelines [27]. The Cronbach's alpha of the Overload scale was 0.791 in this study.

Physical health: The caregiver's physical health using self-rated health with one general question: "How do you rate your general health?" Responses included "very poor", "poor", "fair", "good", and "very good". For data analysis, we treated the original responses as a binary variable of poor (1= very poor, poor, fair) and good (2= good, very good).

Depression: Caregivers' depression was assessed using the Center for Epidemiologic Studies Depression Scale (CES-D) developed by Radloff [30]. The 20-item CES-D scale asks respondents how often they had experienced depressive symptoms in the past week. Scores of ≥16 indicated the presence of clinically significant depression and <16 was classified as no depression [31]. The Cronbach's alpha for the CES-D was 0.877 in this study.

Social support: Social support was evaluated using the 10-item the Social Support Rating Scale (SSRS), which was designed for the Chinese population by Xiao, based on the Social Support Questionnaire (SSQ) and Interview Schedule for Social Interaction (ISSI) [32,33]. The SSRS includes subjective support, objective support, and support utilization. Total score ranges from 12 to 66 [34]. Social support can be divided into three levels: low-level support (≤22), medium-level support (23-44), and high-level support (≥45). In this study, the Cronbach's alpha for the SSRS was 0.804.

2.3 Statistics analysis

SPSS 26.0 was used for the descriptive statistics of all variables to understand the basic characteristics of PwD and their informal caregivers. Each item score on the ECS serves as explicit indicators to establish a LPA model using Mplus 8.3. The most suitable model was selected based on fit indices [35], including the Akaike Information Criterion (AIC), Bayesian Information Criterion (BIC), and adjusted Bayesian Information Criterion (aBIC), Lo-Mendell-Rubin likelihood ratio test (LMR), Bootstrapped likelihood ratio test (BLRT), and Entropy. Lower values of AIC, BIC, and aBIC indicated a superior model fit. Differences between latent profile models were compared using the LMR and BLRT. A significant *P*-value suggests that the model with *k* classes fits the data better than the model with *k*-1 classes. Entropy was used to assess classification accuracy, with values closer to 1 indicating more precise classification; an entropy value ≥0.8 indicates a classification accuracy exceeding 90%. Cohen's d was calculated to further validate the accuracy of the classification (0.2-0.5: small; 0.5-0.8: medium; >0.8: large) [36,37]. To examine the effects of predictor variables on latent profile of QoC, we employed the R3STEP command in Mplus, which effectively controlling for potential effects arises from classification errors [38]. Receiver operating characteristic (ROC) analysis was conducted to determine the optimal cut-off value for the ECS using SAS 9.4. The optimal cut-off point was defined as the value that maximized Youden's index, which maximizes the sum of sensitivity and specificity. Youden's index ranges from 0 to 1, with higher values indicating better classification performance [39]. The area under the curve (AUC) was computed to evaluate the accuracy of identifying low QoC.

AUC values were interpreted as follows: ⩾0.90 (excellent), 0.80-0.89 (good), 0.70-0.79 (fair), and <0.70 (poor). The *P*-value <0.05 indicated statistical significance.

3. Results

3.1 Sample characteristics

A total of 213 dyads of PwD and their informal caregivers were recruited for the study. The average age of PwD was 77.29±9.42, mainly cared for by their spouse or children. Caregivers was predominantly were female (64.8%). 75.6% caregivers lived with PwD. Other information is shown in Table 1.

Table 1 Characteristics of PwD and their informal caregivers (N=213)

PwD	M±SD or n (%)	Informal caregivers	M±SD or n (%)
Age(years)	77.29±9.42	Age(years)	59.06±14.33
Gender		Gender	
Male	117 (54.9)	Male	75 (35.2)
Female	96 (45.1)	Female	138 (64.8)
Education		Education	
Primary school or below	112 (52.6)	Primary school or below	45 (21.1)
Secondary school	87 (40.8)	Secondary school	94 (44.1)
High school or above	14 (6.6)	High school or above	74 (34.8)
Type of dementia		Relationship with PwD	
Alzheimer	128 (60.1)	Spouse	76 (35.7)
Vascular dementia	71 (33.3)	Children	110 (51.6)
Other	14 (6.6)	Other	27 (12.7)
Years of illness		Living with PwD	
～1	58 (27.2)	Yes	161 (75.6)
～5	99 (46.5)	No	52 (24.4)
≥5	56 (26.3)	Length of care (year)	
Numbers of children		～1	70 (32.9)
0	1 (0.5)	～5	97 (45.5)
1	50 (23.5)	≥5	46 (21.6)
2	70 (32.8)	Affordability of living expenses	
≥3	92 (43.2)	Difficult	62 (29.1)

ADL		Somewhat difficult	67 (31.5)
Severe	94 (44.1)	Not difficult	84 (39.4)
Moderate	43 (20.2)	Physical health	
Mild	56 (26.3)	Poor	140 (65.7)
No	20 (9.4)	Good	73 (34.3)
Residence		Depression	
Rural	70 (32.9)	No	144 (67.6)
Urban and town	143 (67.1)	Yes	69 (32.4)
		Quality of pre-illness relationship	11.68±2.82
		Perceived overload	9.63±2.90
		Social support	39.25±7.04

3.2 Latent profile analysis

The fit indices from the 1- to 5-profile QoC among informal caregivers of PwD were shown in Table 2. The AIC, BIC, and aBIC values decreased continuously as the number of profiles gradually increased. Since the *P* values of LMR were >0.05 in the 4-profile model, this model was excluded. The *P* values of LMR and BLRT were significant for the 2-, 3-, and 5-profile models, indicating that the posterior models (3- and 5-profile) were better than the previous model. Although the entropy of the 5-profile model was higher than that of the 3-profile model, and the AIC, BIC, and aBIC values were lower than that of the 3-profile, more profiles indicated that the effective information was more dispersed. Therefore, we chose the 3-profile model as the final fitting model. The average probability of QoC belonging to the profile ranged from 92.0% to 95.2% (see Table 3), and the Cohen's d values of the three-profile model were both higher than 0.80, indicating that the results of the 3-profile models were reasonable. The details are shown in Table 4.

Table 2 Indicators for each latent profile of QoC

profile	Likelihood	AIC	BIC	aBIC	LMR(*P*)	BLRT(*P*)	Entropy	Proportion

1	-2596.885	5237.769	5311.718	5242.006	-	-	-	-
2	-2370.772	4809.544	4923.828	4816.092	0.000	0.000	0.892	0.338/0.662
**3**	**-2289.612**	**4671.224**	**4825.844**	**4680.083**	**0.016**	**0.000**	**0.855**	**0.244/0.446/0.310**
4	-2250.044	4616.088	4811.043	4627.258	0.517	0.000	0.851	0.300/0.174/0.235/0.291
5	-2017.855	4283.771	4519.061	4297.251	0.017	0.000	0.956	0.146/0.150/0.113/0.221/0.371

Table 3 Average probability of attribution for each potential profile

Class	Profile 1	Profile 2	Profile 3
Profile 1	0.952	0.000	0.048
Profile 2	0.000	0.920	0.080
Profile 3	0.028	0.046	0.927

Table 4 Descriptive information of the ECS total score for each profile from LPA

	M(SD)	N(%)	Score Ranges	Cohen's d
LPA[a]				
Low QoC	11.35 (2.10)	66 (30.99%)	[5,16]	d_{2-1}=3.24
Moderate QoC	18.13 (2.09)	95 (44.60%)	[12,22]	d_{3-2}=2.06
High QoC	22.73 (2.51)	52 (24.41%)	[19,29]	d_{3-1}=4.97

Cohen's $d_{2–1}$: the standardized mean difference between moderate-level QoC and low-level QoC; Cohen's $d_{3–1}$: the standardized mean difference between high-level QoC and low-level QoC; Cohen's $d_{3–2}$: the standardized mean difference between high-level QoC and moderate-level QoC.

The three potential profiles of QoC among informal caregivers of PwD scored differently on the eleven items and exhibited distinct characteristics. According to the scoring characteristics, Profile 1 had the highest average score in each item, so named high QoC, accounting for 24.4% of the total. Profile 2 had scores for all items in a middle range, hence named moderate QoC, accounting for 44.6%. Profile 3 scores for each item were much lower than those of Profiles 1 and 2, named low QoC, accounting for 31.0%. The scores of the three profiles on the eleven item of QoC are depicted in Fig.1.

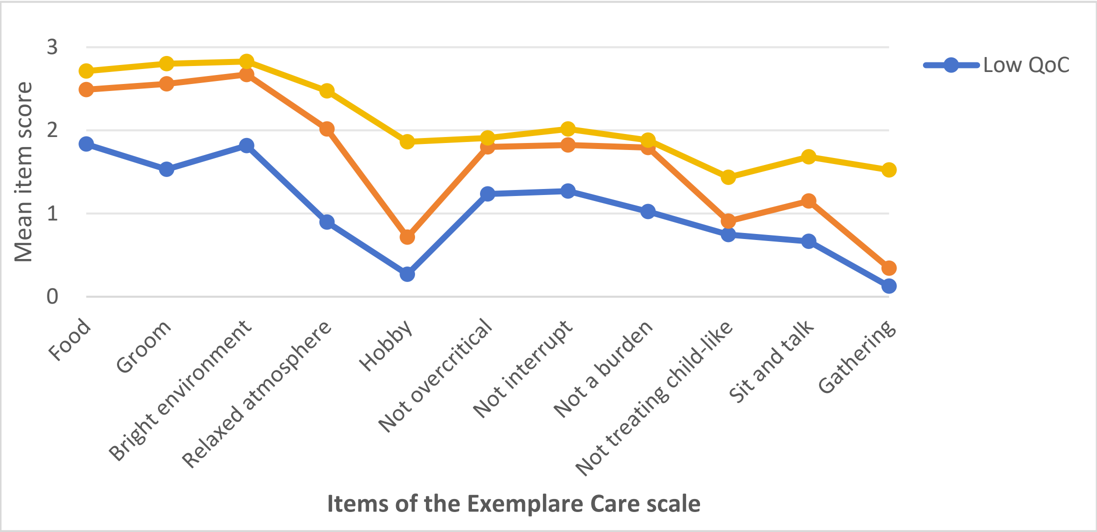

Fig. 1. Latent profile model of QoC among informal caregivers of PwD

3.3 Influencing factors analysis

Utilizing the final potential profile, this study employed a multinomial logistic regression based on the R3STEP procedure to examine the impact on three potential profile. Table 4 displays the outcomes of the logistic regression model with "moderate QoC" serving as the reference category.

The results in Table 5 showed that worse affordability of living expenses (β= -1.420, P<0.05), insufficient social support (β= -0.201, P<0.01), and higher perceived overload (β=0.344, P<0.01) was associated with low QoC group; better quality of pre-illness relationship (β=0.315, P<0.01) and PwD's superior ADL performance (β=1.082, P<0.001) were associated with high QoC group.

Table 5 Test of categorical latent variable multinomial logistic regressions using the 3-step procedure

Variable	low VS moderate (ref)		high VS moderate(ref)	
	β	SE	β	SE
**background variables**				
Affordability of living expenses	-1.420*	0.673	-0.200	0.485
caregiver Quality of pre-illness relationship	-0.251	0.130	0.315**	0.118

**stressors**				
PwD ADL	-0.063	0.380	1.082***	0.291
caregiver Perceived overload	0.344**	0.132	0.064	0.137
**caregiver resource**				
caregiver Social support	-0.201**	0.064	0.068	0.057

Note: *$P < 0.05$; **$P < 0.01$; ***$P < 0.001$

3.4 ROC analysis results

Participants in the “high QoC” and “moderate QoC” profile were coded as “cases” while those in the “low QoC” profiles were coded as “non-cases”. Using this reference standard, the optimal cut-off point was 15, resulting in the highest Youden's index of 0.948 (see Table 6). This cut-off point had high sensitivity (0.993) and specificity (0.955). Additionally, Fig. 2 showed an AUC of 0.995 (95 % CI [0.988, 0.999]; $P < 0.001$), further supporting this cut-off point. The mean differences between the two groups were statistically significant, with a large effect size (see Table 7).

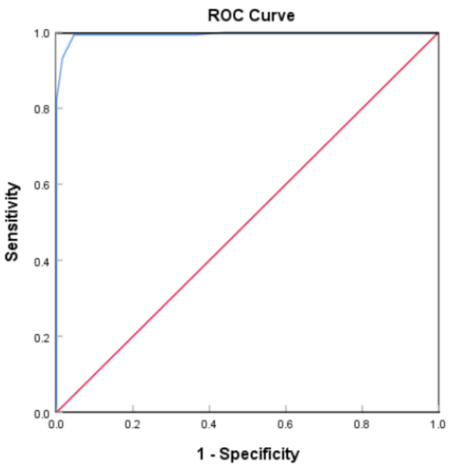

Fig. 2. ROC curve for the classification of moderate and high QoC.

Table 6 Cut-off point based on the “moderate QoC” and “high QoC” group derived through LPA

Cut-off point	Sensitivity	Specificity	Youden’s index
13	0.993	0.636	0.629
14	0.993	0.848	0.841
15	0.993	0.955	0.948
16	0.932	0.985	0.917

Note: Estimates in italic typeface are the suggested optimal cut-off point.

Table 7 Descriptive information of the positive/negative group from ROC analysis.

	M(SD)	N(%)	Score Ranges	Cohen's d
ROC				
Negative group	11.35 (2.10)	66 (30.99%)	[5,16]	d=2.93
Positive group	19.76 (3.14)	147 (69.01%)	[12,29]	

4. Discussion

Using LPA to classify subgroups of QoC among caregivers of PwD, the results showed QoC has explicit classification characteristics. Based on Extended Stress Model, this study examined how background variables, primary stressors, caregivers' quality of life, and caregiver resources predicted different profiles of quality of informal care. Overall, lower cost of living affordability, higher perceived overload and inadequate social support were related to the low QoC group; better quality of the pre-illness relationship and superior ADL performance of PwD were linked to the high QoC group. The cut-off point of the ECS was determined at 15 using ROC analysis, providing an initial reference standard for future screening and intervention studies.

We used LPA to categorize the caregivers of PwD into three distinct profiles, i.e., high QoC (24.41%), moderate QoC (44.60%), and low QoC (30.99%). Statistically significant differences were found between the high and moderate QoC profile in terms of pre-illness relationship quality and PwD' ADL. Logistic regression analysis revealed that the high QoC profile was linked to great quality of pre-illness relationship and superior PwD' ADL. Similarly, significant differences in economic status, perceived overload, and social support were observed between the low and moderate QoC profile. The low QoC profile was associated with low economic

affordability, high perceived overload, and inadequate social support. These results highlight that tailored strategies based on the distinct characteristics of each caregiver subgroup. Priority should be given to caregivers of PwD facing financial strain, high perceived overload, and insufficient social support.

The Extended Stress Process Model hints that background variables, primary stressors, quality of life, and caregiver resources collectively influence the quality of informal care. Our study verified that background variables, primary stressors, and caregiver resources impacted the quality of informal care, except caregivers' quality of life. Specifically, affordability of living expenses, pre-illness relationship quality, the ADL of PwD, perceived overload, and social support decided three QoC profiles. These findings provide an innovative perspective on the importance of economic status, relationship quality, caregiving stress, social support in mitigating the deterioration of QoC among caregivers of PwD.

Family affordability of living expense was a crucial factor for QoC, resonating with other researches [40,41]. Caregivers with higher family economic levels are more likely to provide more resources during the treatment and rehabilitation process, which helps improve the QoC and patient prognosis. Align with previous studies [15,42], a positive pre-illness relationship serves as a protective factor for QoC. As Steadman et al. demonstrated [43], caregivers reporting higher premorbid relationship satisfaction showed significantly less stress and less reactivity to memory and behavioral problems, and better problem solving skills and more effective communication. Conversely, caregivers with poor pre-illness relationships may

perceive caregiving responsibilities as an additional burden, leading to negative attitudes and behaviors [41,43], ultimately compromising the quality of informal care. The finding emphasizes the critical role of caregiving context during care, suggesting that interventions aimed at improving QoC should consider pre-illness relationship quality.

PwD's ADL is positively correlated with the QoC, whereas caregivers overload shows a negative correlation, a conclusion supported by extensive research [7,40,41,42,44]. Caregivers experience tremendous stress and high levels of overload in the long-term care of the elderly PwD [45], while juggling familial, occupational, and caregiving responsibilities. Long-term high-intensity caregiving deteriorates caregivers' mental health and caregivers' ability to provide care, further diminishing the QoC [46,47]. The decline in ADL among PwD significantly increases caregiver overload [48]. Research has shown that cognitive impairment or psychiatric symptoms are prevalent in most patients at the time of diagnosis [3], substantially compromising their daily functioning and necessitating caregiver assistance. However, many caregivers of PwD struggle to provide consistent and attentive care due to insufficient care capacity and a lack of care resources, ultimately resulting in diminished quality of care [49]. Furthermore, disability is often accompanied by aphasia and comprehension deficits, making it difficult for patients to express their needs accurately. These challenges may undermine caregivers' patience and lead to inadequate care. As the PwD's ADL gradually decrease, the level of QoC may be dropped from high to moderate or even low. Thus, timely assessment of ADL are

critical to improve the QoC.

Adequate social support helps preventing low QOC, consisting other studies [44,50]. The buffering model of social support theory suggests that social support acts as a protective factor, reducing the adverse impact of stressful events on individuals [51]. However, the current caregiving system in China remains underdeveloped [52], with caregivers predominantly relying on informal family support networks, while formal support resources at both community and national levels are insufficient [53]. This unidimensional and incomplete support system highlights the establishment of a diversified social support network as a crucial intervention target for improving care quality. Moving forward, it is imperative to strengthen community- and national-level support to provide caregivers with more resources and assistance, collectively building a more comprehensive caregiving system.

Notably, unlike other studies [7,47], this research did not find that caregiver depression affects the QoC provided by informal caregivers, which may be related to the issue of mental health stigma. In China, caregivers may be reluctant to acknowledge their depressive feelings, or societal expectations may pressure them to conceal these emotions [54]. This phenomenon may lead to an underestimation of depression cases in research, resulting in an incomplete reflection of its true impact. Therefore, future studies should further investigate the relationship between caregiver depression and the quality of informal care provided by informal caregivers in the context of Chinese cultural scene, ultimately developing a culturally adapted QoC assessment model.

Combining LPA and ROC analysis, the study revealed that a cut-off point of 15 had the highest sensitivity and specificity for identifying individuals with low quality of informal care, which may provide guidance for future research on the quality of informal care for PwD. The determination of the cut-off value is of great significance for identifying those with low QoC for PwD. Public health professionals are suggested to collect information on the quality of informal care for PwD, implement corresponding interventions for family caregivers of PwD whose scores are less than 15, and provide necessary support. This study deepened our understanding of the quality of informal care for PwD and provided feasible suggestions for public health professionals to improve quality of informal care for PwD in the future.

4.4 Limitations

First, due to the cross-sectional design, causal relationships cannot be established. Future longitudinal studies are needed to strengthen the conclusions. Second, the study only included informal caregivers of PwD in one city in China, which may restrict the generalizability of the findings. Future research should expand the sample to caregivers from diverse regions and urbanization levels to enhance external validity. Third, although this study reported high sensitivity and specificity, the ECS cut-off value of 15 was determined through ROC analysis. Future validation in different samples are needed to enhance the clinical applicability of this threshold. Finally, the self-reported ECS may be influenced by social desirability bias and subjective reporting errors. Future studies could incorporate objective measures (e.g., clinical

assessments, or behavioral observations) to improve data reliability.

5. Conclusion

In this study, we used the concept of "exemplary care" to assess the QoC provided by informal caregivers for PwD in terms of the Provide dimension and the Respect dimension. Using LPA techniques, low, moderate, and high QoC profiles were identified, indicating the heterogeneity of the quality of informal care provided by caregivers for PwD. Based on McClendon's Extended Stress Process Model, we found that worse cost-of-living affordability, inadequate social support and high perceived overload significantly predicted low QoC; better pre-morbid relationships and superior ADL of PwD were predictors of high QoC. Public health professionals should comprehensively consider these factors. Targeted interventions should be applied simultaneously to homogeneous informal caregivers to improve the quality of informal care and prevent a decline in quality of informal care. Furthermore, this study identified the precise critical value of ECS, providing a valuable tool for screening low QoC in PwD and aiding in the identification of PwD and caregivers who may benefit from tailored intervention measures.

Abbreviations

QoC: quality of care; PwD: patients with dementia; ECS: Exemplary Care Scale; RRS: Relationship Rewards Scale; BI: The Barthel Index; CES-D: Epidemiologic Studies Depression Scale; SSRS: Social Support Rating Scale; LPA: Latent profile analysis;

ADL: activity of daily living; CRs: care recipients; CGs: caregivers; ref: reference; CI: confidence interval.

Ethical Approval

The studies involving humans were approved by Ethics Committee of TianYou Hospital Affiliated to Wuhan University of Science &Technology, Wuhan 430064, China. The studies were conducted in accordance with the local legislation and institutional requirements (LL-2023-07-14-002). The participants provided their written informed consent to participate in this study.

Data Availability statement

The data used or analyzed in this study may be obtained from the corresponding author upon reasonable request.

Funding

This research received no specific grant from any funding agency in the public, commercial, or not-for-profit sectors.

Declaration of interest statement

The authors declare that they have no conflict of interest.

Acknowledgments

We are grateful to all the people who agreed to participate in this study for their support, time, and patience. We also thank all staff in the hospital and community centers for their patience and cooperation.

References

[1] 2018 Chinese Guidelines for the Diagnosis and Treatment of Dementia and Cognitive Impairment (Part 1): Dementia and Its Classification Diagnostic Criteria [J] Chinese Medical Journal. 2018,98 (13): 965-970. DOI: 10.3760/cma.j.issn.0376-2491.2018.13.003

[2] Livingston G, Huntley J, Sommerlad A, et al. Dementia prevention, intervention, and care: 2020 report of the Lancet Commission. Lancet. 2020;396(10248):413-446. doi:10.1016/S0140-6736(20)30367-6.

[3] Jia L, Quan M, Fu Y, et al. Dementia in China: epidemiology, clinical management, and research advances. Lancet Neurol. 2020;19(1):81-92. doi:10.1016/S1474-4422(19)30290-X.

[4] Jia L, Du Y, Chu L, et al. Prevalence, risk factors, and management of dementia and mild cognitive impairment in adults aged 60 years or older in China: a cross-sectional study. Lancet Public Health. 2020;5(12):e661-e671. doi:10.1016/S2468-2667(20)30185-7.

[5] Parker LJ, Fabius C, Rivers E, Taylor JL. Is Dementia-Specific Caregiving Compared With Non-Dementia Caregiving Associated With Physical Difficulty Among Caregivers for Community-Dwelling Adults?. J Appl Gerontol. 2022;41(4):1074-1080. doi:10.1177/07334648211014352

[6] Wang M, Sun H, Zhang J, Ruan J. Prevalence and associated factors of elder abuse in family caregivers of older people with dementia in central China cross-sectional study. Int J Geriatr Psychiatry. 2019;34(2):299-307. doi:10.1002/gps.5020

[7] Beach, S. R., Schulz, R., Williamson, G. M., et al. (2005). Risk factors for potentially harmful informal caregiver behavior. Journal of the American Geriatrics Society, 53(2), 255–261. https://doi.org/10.1111/j.1532-5415.2005.53111.x

[8] McClendon, M. J., & Smyth, K. A. (2015). Quality of in-home care, long-term care placement, and the survival of persons with dementia. Aging & mental health, 19(12), 1093–1102. https://doi.org/10.1080/13607863.2014.1003284.

[9] Josephson S. A. (2016). Focusing on transitions of care: A change is here. Neurology. Clinical practice, 6(2), 183–189. https://doi.org/10.1212/CPJ.0000000000000207.

[10] Christie, J., Smith, G. R., Williamson, G. M., et al. (2009). Quality of informal care is multidimensional. Rehabilitation psychology, 54(2), 173–181. https://doi.org/10.1037/a0015705.

[11] Dooley, W. K., Shaffer, D. R., Lance, C. E., & Williamson, G. M. (2007). Informal care can be better than adequate: Development and evaluation of the Exemplary Care Scale. Rehabilitation Psychology, 52(4), 359–369. https://doi.org/10.1037/0090-5550.52.4.359.

[12] Phillips LR, Morrison EF, Chae YM. The QUALCARE Scale: developing an instrument to measure quality of home care. Int J Nurs Stud. 1990;27(1):61-75. doi:10.1016/0020-7489(90)90024-d

[13] Shyu YI, Lee HC, Chen ML. Development and testing of the Family Caregiving

Consequences Inventory for home nursing assessment in Taiwan. J Adv Nurs. 1999;30(3):646-654. doi:10.1046/j.1365-2648.1999.01136.x
[14] Sasaki M, Arai Y, Kumamoto K, Abe K, Arai A, Mizuno Y. Factors related to potentially harmful behaviors towards disabled older people by family caregivers in Japan. Int J Geriatr Psychiatry. 2007;22(3):250-257. doi:10.1002/gps.1670.
[15] Williamson GM, Shaffer DR. Relationship quality and potentially harmful behaviors by spousal caregivers: how we were then, how we are now. The Family Relationships in Late Life Project. Psychol Aging. 2001;16(2):217-226.
[16] McClendon, M. J., & Smyth, K. A. (2013). Quality of informal care for persons with dementia: dimensions and correlates. Aging & mental health, 17(8), 1003–1015. https://doi.org/10.1080/13607863.2013.805400.
[17] Morlett Paredes A, Perrin PB, Peralta SV, et al. Structural equation model linking dementia cognitive functioning, caregiver mental health, burden, and quality of informal care in Argentina. Dementia (London). 2017;16(6):766-779. doi:10.1177/1471301215617080
[18] Lau, B. H. P., Lou, V. W., & Cheung, K. S. L. (2018). Exemplary Care among Chinese Dementia Familial Caregivers. Healthcare (Basel, Switzerland), 6(4), 141. https://doi.org/10.3390/healthcare6040141
[19] Panyavin I, Trujillo MA, Peralta SV, et al. Examining the influence of family dynamics on quality of care by informal caregivers of patients with Alzheimer's dementia in Argentina. Am J Alzheimers Dis Other Demen. 2015;30(6):613-621. doi:10.1177/1533317515577129
[20] Harris GM, Durkin DW, Allen RS, DeCoster J, Burgio LD. Exemplary care as a mediator of the effects of caregiver subjective appraisal and emotional outcomes. Gerontologist. 2011;51(3):332-342. doi:10.1093/geront/gnr003
[21] Yan E, Lai DWL, Sun R, et al. Typology of family caregivers of older persons: A latent profile analysis using elder mistreatment risk and protective factors. J Elder Abuse Negl. 2023;35(1):34-64. doi:10.1080/08946566.2023.2197269
[22] Collins LM, Lanza ST. Latent Class and Latent Transition Analysis: With Applications in the Social, Behavioral, and Health Sciences. USA: John Wiley & Sons; 2009.
[23] Organization WH. The ICD-10 Classification of Mental and Behavioral Disorders. Diagnostic Criteria for Research. Geneva: Organization WH; 1993.
[24] Xu, Y., Chen, Z., Tang, X. et al. Latent profile analysis of nutrition knowledge, attitudes, and practices and their influencing factors in maintenance hemodialysis patients. Sci Rep 15, 17246 (2025). https://doi.org/10.1038/s41598-025-02142-4.
[25] Chen X, Wang Z, Wang S, et al. Latent profiles of ambivalence over emotional expression in young breast cancer patients: A cross-sectional study. Int J Nurs Sci. 2025;12(4):379-385. Published 2025 Jun 16. doi:10.1016/j.ijnss.2025.06.005.
[26] Pearlin, L. I., Mullan, J. T., Semple, S. J., & Skaff, M. M. (1990). Caregiving and the stress process: an overview of concepts and their measures. The Gerontologist, 30(5), 583–594. https://doi.org/10.1093/geront/30.5.583.
[27] Brislin, R.W. (1980) Translation and content analysis of oral and written material. In: Triandis, H.C. and Berry, J. W., Eds., Handbook of cross-cultural psychology: Methodology, Allyn and Bacon, Boston, 389-444.
[28] Leung, S. O., Chan, C. C., & Shah, S. (2007). Development of a Chinese version of the Modified Barthel Index-- validity and reliability. Clinical rehabilitation, 21(10), 912–922.

https://doi.org/10.1177/0269215507077286.

[29] Shah, S., Vanclay, F., & Cooper, B. (1989). Improving the sensitivity of the Barthel Index for stroke rehabilitation. Journal of clinical epidemiology, 42(8), 703 – 709. https://doi.org/10.1016/0895-4356(89)90065-6.

[30] Radloff, L. S. (1977). The CES-D Scale: A Self-Report Depression Scale for Research in the General Population. Applied Psychological Measurement, 1(3), 385-401. https://doi.org/10.1177/014662167700100306.

[31] Thompson T, Rodebaugh TL, Haun P, et al. Longitudinal Dyadic Analysis of Psychosocial and Health Outcomes in Black Breast Cancer Patients and Their Informal Caregivers. Cancer Control. 2025;32:10732748251362950. doi:10.1177/10732748251362950

[32] Sarason, I. G., Johnson, J. H. (1981). A technical report. Washington D. C. : University of Washington.

[33] Henderson, S., Byrne, D.G., Duncan-Jones, P. (1982). Neurosis and the Social Environment.

[34] Xiao, S. Y. The theoretical basis and research application of Social Support Rating Scale. Journal of Clinical Psychological Medicine. 1994,2, 98 – 100. (in Chinese)

[35] Kim, S. Y. (2014). Determining the Number of Latent Classes in Single- and Multiphase Growth Mixture Models. Structural Equation Modeling: A Multidisciplinary Journal, 21(2), 263 – 279. https://doi.org/10.1080/10705511.2014.882690.

[36] Cohen, J. (1988). Statistical power analysis for the behavioral sciences (2nd ed.). New Jersey: Lawrence Erlbaum.

[37] Luo, J., Bei, D., Gong, J., & Wang, M. (2024). Classification of nomophobia among Chinese college students: Evidence from latent profile and ROC analysis. Journal of Behavioral Addictions, 13(2), 482-494. https://doi.org/10.1556/2006.2024.00013

[38] Gabriel AS, Daniels MA, Diefendorff JM, Greguras GJ. Emotional labor actors: a latent profile analysis of emotional labor strategies. J Appl Psychol. 2015;100(3):863-879. doi:10.1037/a0037408.

[39] Nahm FS. Receiver operating characteristic curve: overview and practical use for clinicians. Korean J Anesthesiology. 2022;75(1):25 – 36. https://doi.org/10.4097/kja.21209.

[40] Burnes D, Pillemer K, Caccamise PL, et al. Prevalence of and Risk Factors for Elder Abuse and Neglect in the Community: A Population-Based Study. J Am Geriatr Soc. 2015;63(9):1906-1912. doi:10.1111/jgs.13601.

[41] Fang B, Yan E, Lai DWL. Risk and protective factors associated with domestic abuse among older Chinese in the People's Republic of China. Arch Gerontol Geriatr. 2019;82:120-127. doi:10.1016/j.archger.2019.02.001.

[42] Orfila F, Coma-Solé M, Cabanas M, et al. Family caregiver mistreatment of the elderly: prevalence of risk and associated factors. BMC Public Health. 2018;18(1):167. Published 2018 Jan 22. doi:10.1186/s12889-018-5067-8

[43] Steadman PL, Tremont G, Davis JD. Premorbid relationship satisfaction and caregiver burden in dementia caregivers. J Geriatr Psychiatry Neurol. 2007;20(2):115-119. doi:10.1177/0891988706298624

[44] Huang, W., He, B., Wang, Y. H., Ma, W. J., & Zhou, J. (2020). Disability Severity and Home-Based Care Quality in Older Adults: The Mediating Effects of Social Support and Caregiver Competence. The journal of nursing research : JNR, 28(6), e122. https://doi.org/10.1097/jnr.0000000000000405.

[45] Sheehan, O. C., Haley, W. E., Howard, V. J., et al. (2021). Stress, Burden, and Well-Being in Dementia and Nondementia Caregivers: Insights From the Caregiving Transitions Study. The Gerontologist, 61(5), 670 – 679. https://doi.org/10.1093/geront/gnaa108

[46] Liu xiaohui, Yang yuyan, Xue xijuan, et al. (2019). The correlation between the quality of family care for disabled elderly and caregiver burden. Chinese Journal of Gerontology. 39(16), 4081-4084. DOI:10.3969/j.issn.1005-9202.2019.16.067

[47] Smith, G. R., Williamson, G. M., Miller, L. S., & Schulz, R. (2011). Depression and quality of informal care: a longitudinal investigation of caregiving stressors. Psychology and aging, 26(3), 584–591. https://doi.org/10.1037/a0022263.

[48] Kim, B., Noh, G.O. & Kim, K. Behavioural and psychological symptoms of dementia in patients with Alzheimer’s disease and family caregiver burden: a path analysis. BMC Geriatr 21, 160 (2021). https://doi.org/10.1186/s12877-021-02109-w

[49] Report on the family survival status of Alzheimer's disease patients in China. Alzheimer’s Disease Chinese, 2020-1. 2019. URL: http://weixin.moreedge.cn/bps_2019/index.php

[50] Serra L, Contador I, Fernández-Calvo B, et al. Resilience and social support as protective factors against abuse of patients with dementia: A study on family caregivers. Int J Geriatr Psychiatry. 2018;33(8):1132-1138. doi:10.1002/gps.4905

[51] Cohen S, Wills TA. Stress, social support, and the buffering hypothesis. Psychol Bull. 1985;98(2):310-357

[52] Li J, Gao G, Zeng Y, et al. Perceived social support in Chinese family caregivers of patients with dementia. Int J Nurs Pract. 2022;28(3):e12945. doi:10.1111/ijn.12945

[53] Wu F, Sheng Y. Social support network, social support, self-efficacy, health-promoting behavior and healthy aging among older adults: A pathway analysis. Arch Gerontol Geriatr. 2019;85:103934. doi:10.1016/j.archger.2019.103934

[54] Xu, X., Li, X. M., Zhang, J., & Wang, W. (2017). Mental Health-Related Stigma in China. Issues in Mental Health Nursing, 39(2), 126 – 134. https://doi.org/10.1080/01612840.2017.1368749

Response to Reviewers

Dear Editor and Reviewers,

Thank you for your detailed and constructive comments on my manuscript. According to the editor and reviewers' comments, we have made extensive corrections to our previous manuscript. Two versions of the revised manuscript are submitted: one with no markings and another with the changes highlighted in yellow for clarity. All authors have reviewed and approved the response letter and the revised manuscript. Below is the point-by-point responses to the editor and reviewers, the red font is the revised original text in the manuscript:

Response to Editor:

1. I agree with one of the reviewers, that more details are necessary on how authors calculated the sample size. Kindly provide all assumptions for sample size estimation for future research.

Revised

Thanks very much for your review of our manuscript. In response, we have supplemented and refined the description of the sample size estimation accordingly.

For multinomial logistic regression, the sample size should be at least 5-10 times the number of independent variables [24]. With 20 variables included in this study and accounting for a potential 20% rate of invalid questionnaires, a minimum of 120 participants was initially required. To enhance the accuracy and validation efficacy of the potential category model, a sample size of at least 200 was deemed necessary [25]. Initially, 248 participants were recruited. However, 35 were excluded due to incomplete responses or other reasons, resulting in 213 valid participants included in the final analysis (85.89% response rate). (lines 165-173)

[24] Xu, Y., Chen, Z., Tang, X. et al. Latent profile analysis of nutrition knowledge, attitudes, and practices and their influencing factors in maintenance hemodialysis patients. Sci Rep 15, 17246 (2025). https://doi.org/10.1038/s41598-025-02142-4.

[25] Chen X, Wang Z, Wang S, et al. Latent profiles of ambivalence over emotional expression in young breast cancer patients: A cross-sectional study. Int J Nurs Sci. 2025;12(4):379-385. Published 2025 Jun 16. doi:10.1016/j.ijnss.2025.06.005.

2. The manuscript need careful proofreading. Kindly revise the manuscript carefully for sentence formatting, internal consistency, and punctuation.

Revised. We have carefully checked and revised the manuscript to ensure that the sentence format, internal consistency and punctuation are correct.

Response to Reviewer 1:

1. "However, few studies have examined heterogeneity within groups of caregivers of PwD or the characteristics of quality of care, resulting in a lack of empirical basis for early identification and precise support for high-risk caregivers. Notably, with the rapidly growing population of PwD in China, informal caregivers are experiencing an increasingly heavy burden of care." While this statement attempts to establish a research gap, it is vague and lacks specificity and supporting evidence. I recommend reframing the gap more precisely, ideally by identifying exactly what has been understudied and how the current study addresses that gap.

Indeedly, while this statement attempts to establish a research gap, it is vague and lacks specificity and supporting evidence. Thank you for your valuable comments. We have revised clearly specify the under-investigated areas within current research and to explicitly elaborate on how our study is designed to address these specific gaps.

Many researches mainly focus on the total score of ECS scale to assess overall QoC and its influencing factors [16-20]. However, these studies adopt a variable-oriented approach, which fails to capture individual differences and obscures heterogeneity among different subgroups. It is essential to identify distinct patterns of the QoC within the group and to implement targeted interventions based on their different characteristics. Although existing research has identified distinct caregiver profiles associated with different types of low QoC in the elderly [21], few studies have examined characteristic within groups of caregivers of PwD, resulting in a lack of empirical basis for precise support for high-risk caregivers of PwD supplied low QoC. (lines 95-104)

[21] Yan E, Lai DWL, Sun R, et al. Typology of family caregivers of older persons: A latent profile analysis using elder mistreatment risk and protective factors. J Elder Abuse Negl. 2023;35(1):34-64. doi:10.1080/08946566.2023.2197269

2. The statistical analysis section (2.3) demonstrates an appropriate selection of methods, particularly in applying LPA and utilizing relevant model fit criteria.

However, the writing suffers from unclear variable definitions, grammatical issues, and imprecise explanations of key statistical concepts. I suggest restructuring the paragraph for clarity, clearly defining all abbreviations, and correcting misleading interpretations (e.g., regarding entropy). These revisions would enhance the section's clarity and improve its support for the study analytical rigor and reproducibility.

Revised.

Thank you for your careful checks. We have made a comprehensive revision to the presentation of the statistical analysis section to ensure clarity and accuracy.

Each item score on the ECS serves as explicit indicators to establish a LPA model using Mplus 8.3. The most suitable model was selected based on fit indices [35], including the Akaike Information Criterion (AIC), Bayesian Information Criterion (BIC), and adjusted Bayesian Information Criterion (aBIC), Lo-Mendell-Rubin likelihood ratio test (LMR), Bootstrapped likelihood ratio test (BLRT), and Entropy. Lower values of AIC, BIC, and aBIC indicated a superior model fit. Differences between latent profile models were compared using the LMR and BLRT. A significant P-value suggests that the model with k classes fits the data better than the model with k-1 classes. Entropy was used to assess classification accuracy, with values closer to 1 indicating more precise classification; an entropy value ≥ 0.8 indicates a classification accuracy exceeding 90%. Cohen's d was calculated to further validate the accuracy of the classification (0.2-0.5: small; 0.5-0.8: medium; >0.8: large) [36,37]. (lines 230-242)

3. The discussion section largely restates that there are three QoC groups, but it does not provide a deep interpretation of what distinguishes these profiles, how meaningful the differences are, or how the findings relate to the theoretical framework.

Revised.

Thank you very much for this insightful and critical comment. We have thoroughly revised the Discussion section to address these points as follows, analyzing the differences and significance

of the three groups of characteristics, as well as linking the results of this study to the theoretical framework.

We used LPA to categorize the caregivers of PwD into three distinct profiles, i.e., high QoC (24.41%), moderate QoC (44.60%), and low QoC (30.99%). Statistically significant differences were found between the high and moderate QoC profile in terms of pre-illness relationship quality and PwD' ADL. Logistic regression analysis revealed that the high QoC profile was linked to great quality of pre-illness relationship and superior PwD' ADL. Similarly, significant differences in economic status, perceived overload, and social support were observed between the low and moderate QoC profile. The low QoC profile was associated with low economic affordability, high perceived overload, and inadequate social support. These results highlight that tailored strategies based on the distinct characteristics of each caregiver subgroup. Priority should be given to caregivers facing financial strain, high perceived overload, and insufficient social support. (lines 328-339)

The Extended Stress Process Model hints that background variables, primary stressors, quality of life, and caregiver resources collectively influence the quality of informal care. Our study verified that background variables, primary stressors, and caregiver resources impacted the quality of informal care, except caregivers' quality of life. Specifically, affordability of living expenses, pre-illness relationship quality, the ADL of PwD, perceived overload, and social support decided three QoC profiles. These findings provide an innovative perspective on the importance of economic status, relationship quality, caregiving stress, social support in mitigating the deterioration of QoC among caregivers of PwD. (lines 340-348)

4. The discussion does not explain why specific predictors (e.g., ADL, pre-illness relationship) influence care quality profiles, nor does it contextualize the findings by comparing them to prior research. A more critical engagement with existing literature would strengthen the discussion and improve the paper contribution to the field.

Revised.

Thanks for your valuable feedback. We have revised to provide a more thorough explanation of how specific predictors (such as Activities of Daily Living and pre-illness relationship quality) influence care quality profiles, and have contextualized our findings through systematic comparisons with previous studies.

Align with previous studies [15,42], a positive pre-illness relationship serves as a protective factor for QoC. As Steadman et al. demonstrated [43], caregivers reporting higher premorbid relationship satisfaction showed significantly less stress and less reactivity to memory and behavioral problems, and better problem solving skills and more effective communication. Conversely, caregivers with poor pre-illness relationships may perceive caregiving responsibilities as an additional burden, leading to negative attitudes and behaviors [41,43], ultimately compromising the quality of informal care. The finding emphasizes the critical role of caregiving context during care, suggesting that interventions aimed at improving QoC should consider pre-illness relationship quality. (lines 352-362)

The decline in ADL among PwD significantly increases caregiver overload [48]. Research has shown that cognitive impairment or psychiatric symptoms are prevalent in most patients at the time of diagnosis [3], substantially compromising their daily functioning and necessitating caregiver assistance. However, many caregivers of PwD struggle to provide consistent and attentive care due to insufficient care capacity and a lack of care resources [49], ultimately resulting in diminished QoC. Furthermore, disability is often accompanied by aphasia and comprehension deficits, making it difficult for patients to express their needs accurately. These challenges may undermine caregivers' patience and lead to inadequate care. (lines 369-378)

[43] Steadman PL, Tremont G, Davis JD. Premorbid relationship satisfaction and caregiver burden in dementia caregivers. J Geriatr Psychiatry Neurol. 2007;20(2):115-119. doi:10.1177/0891988706298624

[48] Kim, B., Noh, G.O. & Kim, K. Behavioural and psychological symptoms of dementia in patients with Alzheimer's disease and family caregiver burden: a path analysis. BMC Geriatr 21, 160 (2021). https://doi.org/10.1186/s12877-021-02109-w

[49] Report on the family survival status of Alzheimer's disease patients in China. Alzheimer's Disease Chinese, 2020-1. 2019. URL: http://weixin.moreedge.cn/bps_2019/index.php

Response to Reviewer 2:

1. It is unclear which approach ultimately led to the final target of >210 participants, even though the sample size calculation section specifies

requirements for both LPA (≥150 participants) and multinomial logistic regression (>200 participants). Could you please clarify if the final target was selected based on the higher of the two estimates or if there was another way that you arrived at the number 213?

In accordance with the Editor's suggestion, the revisions have been incorporated. Please see Response to Editor Answer No.1 (lines 165-173 in the revised manuscript).

2. The sample size calculation assumes 3 latent profiles, but this assumption is not supported by previous research or pilot results. Giving a reference will assure readers of its validity, since the assumed number of profiles directly affects the sample size needed for LPA.

Revised.

Thanks the reviewer for this important comment. The assumption regarding the number of potential profiles in the initial submission was not sufficiently grounded. In response, we have removed the unsupported statement and revised.

To enhance the accuracy and validation efficacy of the potential category model, a sample size of at least 200 was deemed necessary [25]. (lines 169-170)

[25] Chen X, Wang Z, Wang S, et al. Latent profiles of ambivalence over emotional expression in young breast cancer patients: A cross-sectional study. Int J Nurs Sci. 2025;12(4):379-385. Published 2025 Jun 16. doi:10.1016/j.ijnss.2025.06.005.

3. It's unclear what is meant by the statement "the mean scores of 11 items of the ECS were used as observed variables." Although it sounds like you averaged the items before performing the analysis, the individual item scores are typically used as the input in latent profile analysis. It appears from Figure 1 that you used the 11-item scores for each individual in the analysis, and then you computed the average scores for each profile to describe them. If this was not what you thought, kindly reword it.

Revised.

We have corrected the incorrect statement in the manuscript.

Each item score on the ECS serves as explicit indicators to establish a LPA model using Mplus 8.3. (lines 230-231)

4. The ECS cut-off of 15 is determined by the ROC analysis, and this shall be validated in a different sample, which would improve its clinical applicability across different populations, even though the reported sensitivity and specificity are high.

Revised.

The original expression of the third limitation has been revised to improve its clarity and precision.

Third, although this study reported high sensitivity and specificity, the ECS cut-off value of 15 was determined through ROC analysis. Future validation in different samples are needed to enhance the clinical applicability of this threshold. (lines 419-421)
