# Supplementary material for: Quality of informal care among informal caregivers of people with dementia: A latent profile and ROC analysis
Source: PLoS One. 2026 Apr 8;21(4):e0346557. doi: 10.1371/journal.pone.0346557 (PMC13061180; doi:10.1371/journal.pone.0346557)
Supplement: Attachment — Submitted filename: reviewer point 7.28.docx [file pone.0346557.s005.docx]

Thank you for the opportunity to review this manuscript. However, the manuscript would benefit from clearer framing of the research gap, improved methodological clarity, and deeper interpretation of results. My comments below aim to support the authors in enhancing the rigor and clarity of the study.

1. *“However, few studies have examined heterogeneity within groups of caregivers of PwD or the characteristics of quality of care, resulting in a lack of empirical basis for early identification and precise support for high-risk caregivers. Notably, with the rapidly growing population of PwD in China, informal caregivers are experiencing an increasingly heavy burden of care.”*

While this statement attempts to establish a research gap, it is vague and lacks specificity and supporting evidence. I recommend reframing the gap more precisely, ideally by identifying exactly what has been understudied and how the current study addresses that gap.

2. The statistical analysis section (2.3) demonstrates an appropriate selection of methods, particularly in applying LPA and utilizing relevant model fit criteria. However, the writing suffers from unclear variable definitions, grammatical issues, and imprecise explanations of key statistical concepts. I suggest restructuring the paragraph for clarity, clearly defining all abbreviations, and correcting misleading interpretations (e.g., regarding entropy). These revisions would enhance the section’s clarity and improve its support for the study analytical rigor and reproducibility.

3. The discussion section largely restates that there are three QoC groups, but it does not provide a deep interpretation of what distinguishes these profiles, how meaningful the differences are, or how the findings relate to the theoretical framework.

4. The discussion does not explain why specific predictors (e.g., ADL, pre-illness relationship) influence care quality profiles, nor does it contextualize the findings by comparing them to prior research. A more critical engagement with existing literature would strengthen the discussion and improve the paper contribution to the field.
