# Supplementary material for: Quality of informal care among informal caregivers of people with dementia: A latent profile and ROC analysis
Source: PLoS One. 2026 Apr 8;21(4):e0346557. doi: 10.1371/journal.pone.0346557 (PMC13061180; doi:10.1371/journal.pone.0346557)
Supplement: Attachment — Submitted filename: Response to Reviewers.docx [file pone.0346557.s009.docx]

Response to Reviewers

Dear Editor and Reviewers,

Thank you for your detailed and constructive comments on my manuscript. According to the editor and reviewers’ comments, we have made extensive corrections to our previous manuscript. Two versions of the revised manuscript are submitted: one with no markings and another with the changes highlighted in yellow for clarity. All authors have reviewed and approved the response letter and the revised manuscript. Below is the point-by-point responses to the editor and reviewers, the red font is the revised original text in the manuscript:

**Response to Editor:**

1. **I agree with one of the reviewers, that more details are necessary on how authors calculated the sample size. Kindly provide all assumptions for sample size estimation for future research.**

Revised

Thanks very much for your review of our manuscript. In response, we have supplemented and refined the description of the sample size estimation accordingly.

For multinomial logistic regression, the sample size should be at least 5-10 times the number of independent variables [24]. With 20 variables included in this study and accounting for a potential 20% rate of invalid questionnaires, a minimum of 120 participants was initially required. To enhance the accuracy and validation efficacy of the potential category model, a sample size of at least 200 was deemed necessary [25]. Initially, 248 participants were recruited. However, 35 were excluded due to incomplete responses or other reasons, resulting in 213 valid participants included in the final analysis (85.89% response rate). (lines 165-173)

[24] Xu, Y., Chen, Z., Tang, X. et al. Latent profile analysis of nutrition knowledge, attitudes, and practices and their influencing factors in maintenance hemodialysis patients. Sci Rep 15, 17246 (2025). <https://doi.org/10.1038/s41598-025-02142-4>.

[25] Chen X, Wang Z, Wang S, et al. Latent profiles of ambivalence over emotional expression in young breast cancer patients: A cross-sectional study. Int J Nurs Sci. 2025;12(4):379-385. Published 2025 Jun 16. doi:10.1016/j.ijnss.2025.06.005.

**2. The manuscript need careful proofreading. Kindly revise the manuscript carefully for sentence formatting, internal consistency, and punctuation.**

Revised. We have carefully checked and revised the manuscript to ensure that the sentence format, internal consistency and punctuation are correct.

**Response to Reviewer 1:**

**1. “However, few studies have examined heterogeneity within groups of caregivers of PwD or the characteristics of quality of care, resulting in a lack of empirical basis for early identification and precise support for high-risk caregivers. Notably, with the rapidly growing population of PwD in China, informal caregivers are experiencing an increasingly heavy burden of care.”**

**While this statement attempts to establish a research gap, it is vague and lacks specificity and supporting evidence. I recommend reframing the gap more precisely, ideally by identifying exactly what has been understudied and how the current study addresses that gap.**

Indeedly, while this statement attempts to establish a research gap, it is vague and lacks specificity and supporting evidence. Thank you for your valuable comments. We have revised clearly specify the under-investigated areas within current research and to explicitly elaborate on how our study is designed to address these specific gaps.

Many researches mainly focus on the total score of ECS scale to assess overall QoC and its influencing factors [16-20]. However, these studies adopt a variable-oriented approach, which fails to capture individual differences and obscures heterogeneity among different subgroups. It is essential to identify distinct patterns of the QoC within the group and to implement targeted interventions based on their different characteristics. Although existing research has identified distinct caregiver profiles associated with different types of low QoC in the elderly [21], few studies have examined characteristic within groups of caregivers of PwD, resulting in a lack of empirical basis for precise support for high-risk caregivers of PwD supplied low QoC. (lines 95-104)

[21] Yan E, Lai DWL, Sun R, et al. Typology of family caregivers of older persons: A latent profile analysis using elder mistreatment risk and protective factors. J Elder Abuse Negl. 2023;35(1):34-64. doi:10.1080/08946566.2023.2197269

**2. The statistical analysis section (2.3) demonstrates an appropriate selection of methods, particularly in applying LPA and utilizing relevant model fit criteria. However, the writing suffers from unclear variable definitions, grammatical issues, and imprecise explanations of key statistical concepts. I suggest restructuring the paragraph for clarity, clearly defining all abbreviations, and correcting misleading interpretations (e.g., regarding entropy). These revisions would enhance the section’s clarity and improve its support for the study analytical rigor and reproducibility.**

Revised.

Thank you for your careful checks. We have made a comprehensive revision to the presentation of the statistical analysis section to ensure clarity and accuracy.

Each item score on the ECS serves as explicit indicators to establish a LPA model using Mplus 8.3. The most suitable model was selected based on fit indices [35], including the Akaike Information Criterion (AIC), Bayesian Information Criterion (BIC), and adjusted Bayesian Information Criterion (aBIC), Lo-Mendell-Rubin likelihood ratio test (LMR), Bootstrapped likelihood ratio test (BLRT), and Entropy. Lower values of AIC, BIC, and aBIC indicated a superior model fit. Differences between latent profile models were compared using the LMR and BLRT. A significant *P*-value suggests that the model with *k* classes fits the data better than the model with *k*-1 classes. Entropy was used to assess classification accuracy, with values closer to 1 indicating more precise classification; an entropy value ≥0.8 indicates a classification accuracy exceeding 90%. Cohen's d was calculated to further validate the accuracy of the classification (0.2-0.5: small; 0.5-0.8: medium; >0.8: large) [36,37]. (lines 230-242)

**3. The discussion section largely restates that there are three QoC groups, but it does not provide a deep interpretation of what distinguishes these profiles, how meaningful the differences are, or how the findings relate to the theoretical framework.**

Revised.

Thank you very much for this insightful and critical comment. We have thoroughly revised the Discussion section to address these points as follows, analyzing the differences and significance of the three groups of characteristics, as well as linking the results of this study to the theoretical framework.

We used LPA to categorize the caregivers of PwD into three distinct profiles, i.e., high QoC (24.41%), moderate QoC (44.60%), and low QoC (30.99%). Statistically significant differences were found between the high and moderate QoC profile in terms of pre-illness relationship quality and PwD’ ADL. Logistic regression analysis revealed that the high QoC profile was linked to great quality of pre-illness relationship and superior PwD’ ADL. Similarly, significant differences in economic status, perceived overload, and social support were observed between the low and moderate QoC profile. The low QoC profile was associated with low economic affordability, high perceived overload, and inadequate social support. These results highlight that tailored strategies based on the distinct characteristics of each caregiver subgroup. Priority should be given to caregivers facing financial strain, high perceived overload, and insufficient social support. (lines 328-339)

The Extended Stress Process Model hints that background variables, primary stressors, quality of life, and caregiver resources collectively influence the quality of informal care. Our study verified that background variables, primary stressors, and caregiver resources impacted the quality of informal care, except caregivers’ quality of life. Specifically, affordability of living expenses, pre-illness relationship quality, the ADL of PwD, perceived overload, and social support decided three QoC profiles. These findings provide an innovative perspective on the importance of economic status, relationship quality, caregiving stress, social support in mitigating the deterioration of QoC among caregivers of PwD. (lines 340-348)

**4. The discussion does not explain why specific predictors (e.g., ADL, pre-illness relationship) influence care quality profiles, nor does it contextualize the findings by comparing them to prior research. A more critical engagement with existing literature would strengthen the discussion and improve the paper contribution to the field.**

Revised.

Thanks for your valuable feedback. We have revised to provide a more thorough explanation of how specific predictors (such as Activities of Daily Living and pre-illness relationship quality) influence care quality profiles, and have contextualized our findings through systematic comparisons with previous studies.

Align with previous studies [15,42], a positive pre-illness relationship serves as a protective factor for QoC. As Steadman et al. demonstrated [43], caregivers reporting higher premorbid relationship satisfaction showed significantly less stress and less reactivity to memory and behavioral problems, and better problem solving skills and more effective communication. Conversely, caregivers with poor pre-illness relationships may perceive caregiving responsibilities as an additional burden, leading to negative attitudes and behaviors [41,43], ultimately compromising the quality of informal care. The finding emphasizes the critical role of caregiving context during care, suggesting that interventions aimed at improving QoC should consider pre-illness relationship quality. (lines 352-362)

The decline in ADL among PwD significantly increases caregiver overload [48]. Research has shown that cognitive impairment or psychiatric symptoms are prevalent in most patients at the time of diagnosis [3], substantially compromising their daily functioning and necessitating caregiver assistance. However, many caregivers of PwD struggle to provide consistent and attentive care due to insufficient care capacity and a lack of care resources [49], ultimately resulting in diminished QoC. Furthermore, disability is often accompanied by aphasia and comprehension deficits, making it difficult for patients to express their needs accurately. These challenges may undermine caregivers’ patience and lead to inadequate care. (lines 369-378)

[43] Steadman PL, Tremont G, Davis JD. Premorbid relationship satisfaction and caregiver burden in dementia caregivers. J Geriatr Psychiatry Neurol. 2007;20(2):115-119. doi:10.1177/0891988706298624

[48] Kim, B., Noh, G.O. & Kim, K. Behavioural and psychological symptoms of dementia in patients with Alzheimer’s disease and family caregiver burden: a path analysis. BMC Geriatr 21, 160 (2021). <https://doi.org/10.1186/s12877-021-02109-w>

[49] Report on the family survival status of Alzheimer's disease patients in China. Alzheimer’s Disease Chinese, 2020-1. 2019. URL: [http://weixin.moreedge.cn/bps_2019/index.php](http://weixin.moreedge.cn/bps_2019/index.php)

**Response to Reviewer 2:**

1. **It is unclear which approach ultimately led to the final target of >210 participants, even though the sample size calculation section specifies requirements for both LPA (≥150 participants) and multinomial logistic regression (>200 participants). Could you please clarify if the final target was selected based on the higher of the two estimates or if there was another way that you arrived at the number 213?**

In accordance with the Editor's suggestion, the revisions have been incorporated. Please see Response to Editor Answer No.1 (lines 165-173 in the revised manuscript).

2. **The sample size calculation assumes 3 latent profiles, but this assumption is not supported by previous research or pilot results. Giving a reference will assure readers of its validity, since the assumed number of profiles directly affects the sample size needed for LPA.**

Revised.

Thanks the reviewer for this important comment. The assumption regarding the number of potential profiles in the initial submission was not sufficiently grounded. In response, we have removed the unsupported statement and revised.

To enhance the accuracy and validation efficacy of the potential category model, a sample size of at least 200 was deemed necessary [25]. (lines 169-170)

[25] Chen X, Wang Z, Wang S, et al. Latent profiles of ambivalence over emotional expression in young breast cancer patients: A cross-sectional study. Int J Nurs Sci. 2025;12(4):379-385. Published 2025 Jun 16. doi:10.1016/j.ijnss.2025.06.005.

3. **It's unclear what is meant by the statement "the mean scores of 11 items of the ECS were used as observed variables." Although it sounds like you averaged the items before performing the analysis, the individual item scores are typically used as the input in latent profile analysis. It appears from Figure 1 that you used the 11-item scores for each individual in the analysis, and then you computed the average scores for each profile to describe them. If this was not what you thought, kindly reword it.**

Revised.

We have corrected the incorrect statement in the manuscript.

Each item score on the ECS serves as explicit indicators to establish a LPA model using Mplus 8.3. (lines 230-231)

**4. The ECS cut-off of 15 is determined by the ROC analysis, and this shall be validated in a different sample, which would improve its clinical applicability across different populations, even though the reported sensitivity and specificity are high.**

Revised.

The original expression of the third limitation has been revised to improve its clarity and precision.

Third, although this study reported high sensitivity and specificity, the ECS cut-off value of 15 was determined through ROC analysis. Future validation in different samples are needed to enhance the clinical applicability of this threshold. (lines 419-421)
