# Supplementary material for: Quality of informal care among informal caregivers of people with dementia: A latent profile and ROC analysis
Source: PLoS One. 2026 Apr 8;21(4):e0346557. doi: 10.1371/journal.pone.0346557 (PMC13061180; doi:10.1371/journal.pone.0346557)
Supplement: Attachment — Submitted filename: Response_to_Reviewers_auresp_2.docx [file pone.0346557.s010.docx]

Response to Reviewers

Dear Editor and Reviewers,

Thank you for your detailed and constructive comments on my manuscript. According to the editor and reviewers’ comments, we have made extensive corrections to our previous manuscript. Two versions of the revised manuscript are submitted: one with no markings and another with the changes highlighted in yellow for clarity. All authors have reviewed and approved the response letter and the revised manuscript. Below is the point-by-point responses to the editor and reviewers, the red font is the revised original text in the manuscript:

**Response to Editor:**

1. **I went through the rebuttals from the authors and the reviewers' comments on the second version of the manuscript. The manuscript is composed of scores of grammatical errors, awkward sentence structure, and confusing statements. Tow of the reviewers did painstaking yet remarkable jobs to identify the shortcomings and provided valuable comments to improve the manuscript. Keeping the importance of the topic and the level of efforts from the authors in mind I would like to give another chance to the manuscript. Me too have some suggestions to improve the manuscript. I am requesting authors to revise and resubmit the manuscript for another round by addressing all the comments form the reviewers. The manuscript is probably drafted and written by someone whose first language is not English. So I will eagerly request authors copy-editing the manuscript by someone who is proficient in academic English before submission.**

Thank you for the decision and feedback. We will have the manuscript professionally copy-edited and fully address all reviewer comments in the revision.

2. **Please provide marginal effect plots or at least a table of mean values along with 95%-CI for independent variables for each of the three groups in the final model. This will provide information to interpret the magnitude and nature of the relationship between predictors and latent profile membership. Instead of just seeing if a variable is a significant predictor, these plots will show how much the probability of profile membership shifts as the independent variable changes.**

Revised.

Thanks very much for your review of our manuscript. Revised as suggested. We have added the OR and 95% CI for independent variables in Table 6. (line 313)

3. **Reviewer from the last round of review asked to validate ECS cut-off of 15 in a different sample to improve its clinical applicability. I suggest dividing the sample into two sub-samples, say 50-50% or 60-40%, using a random number generator. Then use one sub-sample to ROC analysis get a cut-off, apply the cut-off to other sub-sample, and calculate the sensitivity and specificity.**

Revised.

Thank you for this important methodological suggestion. We have followed the recommended procedure: randomly splitting the sample (50-50%), deriving the cut-off in one subsample, and validating it in the other. The analysis robustly confirmed the ECS cut-off of 15 in both independent subsamples, supporting its reliability. The detailed results have been added to the manuscript.

To validate the clinical applicability of the ECS cut-off, the sample was randomly split into two independent subsamples (approximately 50%/50%). In the derivation subsample, ROC analysis identified an optimal ECS cut-off score of 15, yielding a high sensitivity of 0.993, specificity of 0.955, and an AUC of 0.998 (95% CI [0.993, 0.999], P < 0.001; Fig. 2; Table 7). The mean difference between the two groups was statistically significant with a large effect size (Table 8), further supporting this cut-off point. In the validation subsample, the identical cut-off of 15 was confirmed, achieving a Youden’s index of 0.928, sensitivity of 0.985, specificity of 0.943, and an AUC of 0.990 (95% CI [0.975, 0.999], P < 0.001). The consistent performance across both subsamples supports the robustness and generalizability of the ECS cut-off score of 15 for identifying low QoC. (line 316-326)

4. **Line 55-56: Please mention the geographic reference for the expected number of 152 million PwD in 2050. Will the number of 152 million reach worldwide, in Asia, or only in China?**

Revised.

Thank you for your valuable comments. We have revised clearly specify the data "152 million in 2050" refers to the global level.

Globally, the number of people with dementia (PwD) is projected to reach 152 million by 2050 [2]. (line 55-56)

5. **Line 69: Please revise as "…that may negatively impact care recipients’ (CRs) well-being, …"**

Revised. (line 70)

6. **Line 70: What does it mean by adequacy of caregiving behaviors? The meaning is unclear to me. Kindly reconstruct the sentence.**

Revised.

Thank you for pointing out this issue. In the revised manuscript, we explicitly define "adequacy of caregiving behaviors" as "the adequacy of caregiving behaviors in meeting the CRs’ basic needs". (line 71)

7. **Line 72-73: Rephrase from "…CRs’ daily basic and instrumental daily needs…" to "…CRs’ basic and instrumental daily needs…"**

Revised. (line 74-75)

8. **Line 104: Change the word ‘supplied’. ‘Provided’ would be a better choice of words here.**

Revised. Thank you for your suggestion. I have revised ‘supplied’ to ‘provided’ and further refined the sentence for clarity and academic tone. The revised sentence now reads:

This gap limits the empirical basis for providing targeted support to high-risk caregivers who provide low QoC for PwD. (line 104-105)

9. **Line 126: Please clarify whether traditional total score analysis assumes "the same score can represent different qualities" or LPA assumes "the same…". If the answer is the latter, please rephrase and bring the last clause in the middle of the sentence.**

Thank you for this correct observation. We agree that the phrase was ambiguously phrased and that the limitation belongs to the traditional score approach. As the specific sentence has been revised following thet suggestion of Reviewer 3 (to state objectives without specifying methods). Please see Response to Reviewer 3 Answer No.10 (line 127-132 in the revised manuscript).

10. **Line 138: Statement #3 is not a hypothesis. To me it is the application of the learnings from hypothesis testing from the first two hypotheses.**

Revised.

Thank you for your precise feedback. We agree that the original statement described an application. Following Reviewer 6’s suggestion, we have revised hypothesis: (3) On this basis, and through ROC analysis, it may be possible to establish an optimal cutoff value with high sensitivity and specificity that allows for the identification of different levels of care quality and, specifically, the identification of low-quality care. (line119-122)

11. **Line 98-102: Do authors mean ‘subgroups’ when they use the word group here? I guess authors wanted to make the justification for LPA here, but the communication is not clear and suddenly the introduction of the concept of group confuses the readers.**

Thank you for this clarification. We have revised the paragraph to address the ambiguity. The text now clearly distinguishes the limitations of the traditional variable-centered approach (focusing on total scores) from the need for a person-centered approach (to identify distinct profiles).

A shift to a person-centered approach is therefore needed to identify distinct caregiver profiles for personalized support. Although such person-centered methods have identified meaningful profiles in elderly care [21], their application specifically to dementia caregivers remains scarce. (line101-104)

12. **Line 160-162: The sentence could be rephrased as "Participants with reading difficulties completed the questionnaires with the help of trained research staff."**

Revised. (line 165-166)

13. **Line 167-170: Thank you for addressing this point raised in the previous round of review. The justifications for the sample size are given for analytical sample. However, readers would like to know the representativeness of the sample along with statistical assumptions like expected value of response distribution, level of confidence, margin of error, power, etc.**

Revised.

Thank you for the valuable comments.We have conducted an a priori power analysis using G*Power and revised the manuscript accordingly, clarifying both the calculation basis and the methodological considerations for determining the sample size.

An a priori sample size calculation was performed using G*Power 3.1.9.7. With a two-tailed, a medium effect size (f^2^=0.30), a power of 0.95, a statistical level α=0.05 [25], and 20 predictors, the minimum required sample size was 120. To ensure robust model fit, stable parameter estimation, and adequate power for subgroup comparisons in LPA, we targeted a minimum of 200 participants [26], consistent with methodological recommendations for person-centered approaches. (line171-176)

14. **Figure 1: Please add legends for all three lines. Technically this should not be a line diagram because the variables in the x-axis are completely independent of each other not showing any trend. It would be better if authors leave the dots and remove the lines connecting the data points. Also, I would recommend adding ‘whiskers’, denoting 95%-CIs of item scores, to each dot. This way authors can show how or if the distributions of scores are overlapped or not. But adding whiskers is not mandatory, if that makes the figure more cluttery.**

Revised.

We add legends for all three lines. We leave the dots and remove the lines connecting the data points and adding ‘whiskers’. (line 299)

15. **Table 5: Remove the row titled ROC if anything else is not provided.**

Thank you for your comment. We have checked Table 5 (now table 6) carefully and found no row titled "ROC" in the current version. The table includes only the variables listed in the submitted manuscript. Could you please clarify which "ROC" you are referring to? We are happy to make the necessary changes once we understand your request.

16. **Line 321. Revise "…life, and caregiver resources predicted…" into "…life, and caregiver resources; and predicted…"**

Thank you for your suggestion. I agree with the proposed revision. However, in response to Reviewer 6’s comment on content redundancy, the sentence cited at Line 321 has been removed during revision. The relevant section no longer appears in the manuscript.

17. **Line 337-338: Authors would need to add a consequence or a finding to this sentence, for example, "...highlight that tailored strategies need to be developed based on the distinct characteristics of each caregiver subgroup."**

Revised. Thank you for your suggestion on Lines 337-338. I agree that a clearer implication is needed. In response to Reviewer 6’s comments, I have rewritten this section.

18. **Line 343: Kindly reconstruct the sentence. I guess, authors want to say here that the caregivers' quality of life was the sole factor among all independent variable examined that did not show a statistically significant effect on the quality of care provided. One version could be "The study confirmed that background variables, primary stressors, and caregiver resources influenced the quality of informal care, but did not find a similar impact from caregivers’ quality of life."**

Revised.

Overall, our findings are largely consistent with Extended Stress Process Model [16], confirming that background variables, primary stressors, and caregiver resources significantly predict the quality of informal care,but did not find a similar impact from caregivers’ quality of life. (line 357-360)

19. **Line 348. These findings provide an innovative perspective on the importance of economic status, relationship quality, caregiving stress, and social support in mitigating the deterioration of QoC among caregivers of PwD. Meaning of the sentence is unclear. Kindly rephrase. Do authors mean low QoC by using the word ‘deterioration’? What is ‘innovative’ perspective on importance of such variables, and how this innovation could ‘mitigate’ the deterioration of QoC (or low QoC)?**

Thank you for your comment on Line 348. I agree the original sentence was unclear. In response to Reviewer 6’s comment that the Discussion should not present new results, I have rewritten this section.The relevant section no longer appears in the manuscript.

20. **Table-5 Kindly properly format the table of multinomial regression. The names of the variables are presented as it is entered into the software. Please rewrite the names of the variables in a meaningful way.**

Thank you for your suggestion. I have revised Table 5 (now Table 6) and rewritten all variable names to ensure they are presented clearly and meaningfully. (line 313)

**Response to Reviewer 3:**

**Authors are commended for their superb study focusing on quality of informal care of a vulnerable patient population. Findings not only highlight the profile of the informal caregiver but also, perhaps unintentionally, provides a sense of the predicament of the care recipient.**

**General comments:**

**The reviewer suggested revision of sentence structures. However, authors are encouraged to thoroughly engage with the manuscript to ensure that all grammatical issues are addressed. Alternatively, authors might consider consulting with a language and/or copy editor.**

Revised. We have carefully checked and revised revision of sentence structures, thoroughly engage with the manuscript to ensure that all grammatical issues are addressed.

**一．Abstract**

1. **Line 26-27: the sentence "Additionally, the absence of clearly thresholds for identifying PwD with low quality of informal care poses a challenge for research and clinical practice" should either read "Additionally, the absence of clear thresholds for identifying PwD with low quality of informal care poses a challenge for research and clinical practice" or alternative, "Additionally, the absence of clearly demarcated [or specified] thresholds for identifying PwD with low quality of informal care poses a challenge for research and clinical practice"**

Revised.

Additionally, the absence of clear thresholds to identify PwD receiving low-quality informal care poses a challenge for research and clinical practice. (line 25-27)

2. **Line 27: it is assumed that authors are referring to PwD needs in "… thresholds for identifying PwD with low quality of informal care [needs]?" Or are they referring to identifying PwD receiving "low quality of informal care" or being delivered to?**

Revised. Thank you for the feedback. We have revised the sentence to eliminate the ambiguity.

Additionally, the absence of clear thresholds to identify PwD receiving low-quality informal care poses a challenge for research and clinical practice. (line 25-27)

3. **Line 28: the word "of" seems misplaced in the sentence structure "… aimed to identify potential profiles of among informal caregivers of PwD…". Furthermore, it is not clear what "potential profiles" authors are referring to? Similarly, it is not clear what authors are referring to with regards to "profile" / "profiles" in the Methods section [i.e line 36]. Furthermore, in the Results section authors refer to quality of care profiles [line 38-39]? It is only after reading the rest of the manuscript that it became clear what "profiles" refer to. This should be remedied.**

Revised.

Thank you for this essential critique regarding the unclear introduction of a core concept. We have remedied this by providing a clear and full definition at the first occurrence of the term “profiles” in the Introduction (Line 28), specifying that they refer to “profiles of quality of care (QoC)”. Subsequently, in the Methods and Results sections, we consistently use this defined term (“QoC profiles”) to ensure clarity and avoid ambiguity.

Thus, this study aimed to identify the profiles of quality of care (QoC) among informal caregivers of PwD, explore influencing factors of different profile, and determine the optimal cut-off value of the Exemplary Care Scale (ECS). (line 27-29)

4. **Line 29: do authors have one profile in mind stating "… explore influencing factors of different profile, …" or should "profile" be revised to read "profiles"?**

Revised. The term of "profile" was revised to "profiles".

5. **Line 39-41: it is not clear whether authors are referring to informal carer’s or PwD’s "worse affordability of living expenses, insufficient social support and higher perceived overloaded"?**

We thank the reviewer for highlighting this ambiguity. The sentence has been revised to explicitly specify the subject.

Among informal caregivers, lower monthly income, insufficient social support, and higher perceived overload were associated with low QoC profile, whereas, better quality of pre-illness relationship with PwD and greater activities of daily living (ADL) of PwD were associated with high QoC. (line 38-41)

6. **Line 45: it is not "obvious" which "classification characteristics" authors are referring to.**

We thank the reviewer for this feedback. The vague phrasing has been replaced with a clear, objective statement of our specific finding.

This study identified three distinct QoC profiles among caregivers of PwD, underscoring the heterogeneity of informal care quality. (line 44-45)

7. **In general, the abstract does not seem to have been written with the same robustness as the body of the manuscript.**

We thank the reviewer for this overall assessment. We have thoroughly revised the abstract to enhance its clarity, precision, and robustness, ensuring it now matches the standard of the manuscript body.

**二、Introduction**

8. **Line 99: it might be helpful to clarify which group authors are referring to stating "within the group" for the reader. It is assumed authors are referring to QoC among informal caregivers?**

Thank you for prompting this clarification. We have revised the sentence to replace the ambiguous phrase “within the group” with the specific focus of our analysis: A shift to a person-centered approach is therefore needed to identify distinct caregiver profiles for personalized support. Although such person-centered methods have identified meaningful profiles in elderly care [21], their application specifically to dementia caregivers remains scarce. (line 101-104)

9. **Line 104-115: it is not clear why authors consider reference to LPA, "an individual-centered statistical analysis", as background versus information related to Materials and Methods? In the event that authors deem reference to LPA necessary in the Introduction section they are encouraged to rephrase LPA-related sentences to meet typical background-related standards. Ultimately, authors need to separate research methodology from practical application / implementation of their findings and move applicable methodology sentences / detail to these sections of the manuscript.**

Thank you for the reviewer's guidance. We have restructured the content accordingly, in the Introduction section, we have removed the specific methodological description of LPA, retaining only the discussion of the theoretical framework and research objectives. In the Materials and Methods section, we have clearly elaborated the analytical procedures of LPA, thereby preserving the integrity of the study.

This study was guided by McClendon's Extended Stress Process Model [16]. The model takes "QoC" as the core outcome variable, shifting the research perspective from "caregiver-centered" to the "caregiver-patient dyadic integration." Including multidimensional influences provides a systematic theoretical framework for understanding QoC in dementia. The results of this study could provide important empirical evidence for the subsequent development of precise intervention strategies for different subgroups of caregivers. (line 133-139)

10. **Line 124-130: ultimately reference to statistical analyses applied [(1) … "using LPA …" (2) "… through logistic regression analysis, …" and (3) "… using ROC analysis …"] reflect authors’ choice of statistical analysis with regard to their study and has no bearing on the objectives of the study. Would authors agree that the same objectives could have been addressed using different statistical approaches?**

Revised. We highly appreciate your constructive comments and fully agree that statistical analysis methods are research tools (not part of study objectives), and the same objectives could be achieved through different statistical approaches. Accordingly, we have revised the statement of objectives. The specific analytical techniques (LPA, logistic regression, ROC analysis) have been removed from the objectives themselves.

Furthermore, following Reviewer 6’s suggestions, we have expanded the number of objectives from three to four and have refined the wording of objective (3) accordingly.

To respond to these hypotheses, we set four objectives: (1) to identify potential subgroups of the quality of care among informal caregivers of PwD,; (2) to clarify the factors associated with these subgroups; (3) to identify categorization thresholds for the ECS that can be applied in clinical and community practice; and (4) to propose rapid screening criteria that enable health and social organizations to quickly identify high-risk individuals. (line 127-132)

11. **Line 138-139: the reviewer is not convinced that (3) qualifies as a "hypothesis" [line 135]? The sentence rather seems to address "2.3 Statistical analysis"?**

Thank you for your precise feedback. We agree that the original statement described an application. Following Reviewer 6’s suggestion, we have revised hypothesis: (3) On this basis, and through ROC analysis, it may be possible to establish an optimal cutoff value with high sensitivity and specificity that allows for the identification of different levels of care quality and, specifically, the identification of low-quality care. (line 119-122)

**三、Materials and Methods**

12. **Line 145: the sentence structure needs to be addressed. It could possibly read "… caregivers attending memory clinics, rehabilitation clinics, and neurological clinics …" or "… caregivers attending either a memory clinic, rehabilitation clinic, and neurological clinic …".**

Revised.

Using convenience sampling, we recruited PwD and their informal caregivers attending memory clinic, rehabilitation clinic, and neurological clinic in the hospital and communities. (line 144-146)

13. **Line 149: is there a reason why authors reference the ICD-10 [1993] versus the ICD-11 [2019]?**

We appreciate the reviewer's attention to this methodological detail. The ICD-10 was referenced because our cross-sectional study included participants whose dementia diagnoses were established in clinical settings prior to data collection. Given the inherent delay between the release of a new classification system (ICD-11 in 2019) and its widespread adoption in routine practice, ICD-10 remained the most relevant and accurate representation of the diagnostic basis for our study at the time of their diagnosis.

14. **Line 154: what does "clear consciousness" entail? How was "normal intelligence" defined? What criteria did authors apply [and/or how did they gauge] to determine inclusion of informal caregivers based on "clear consciousness, normal intelligence, good communication skills"?**

Revised.

Thank you for pointing out the lack of clarity in the inclusion criteria. Following your suggestion, we have replaced the subjective descriptors such as "clear consciousness" and "normal intelligence" with observable and verifiable behavioral criteria. We have also added that all participants were assessed by researchers to ensure their full comprehension of the study.

(4) provide informed consent; and (5) were able to read, comprehend the Chinese questionnaire, and communicate effectively in Mandarin (the official language of China). Caregivers were excluded if they had experienced other major stressful life events (e.g., bereavement or divorce) within the past three months. All participants were assessed by researchers to ensure they understood the study. (line 154-159)

15. **Line 157-174: authors include among these sentences detail regarding a) data collection b) sample size calculation c) ethics approval. It is suggested that authors provide applicable sub-headings to distinguish between various Materials and Method aspects. Streamlining of sub-headings and sentence structures will enhance their paper.**

Revised. Thank you for your suggestion. We have added corresponding subheadings: 2.2 Data collection (line 160), 2.3 Sample size calculation (line 170), and 2.4 Ethical approval (line 180) in the Materials and Methods section to clearly distinguish the different components and improve structural clarity.

16. **Line 157: it is not clear whether "paper questionnaires" and "independent questionnaires" refer to the ESC caregivers completed or were different questionnaires completed? It might be worth mentioning how many questionnaires [name / label these] participants were expected to complete [possibly in table format] especially given the extended number of questionnaires caregivers were expected to complete [as referred to in 2.2.2]. Such clarity will also assist to grasp the extensive carers profile authors obtained? Did both PwD and the caregivers complete questionnaires independently. Who provided basic information of PwD [line 187]?**

Thank you for the questions.

(1) We have clarified in the manuscript that only caregivers completed the questionnaires, providing all information (including that of the PwD) as proxy respondents.

Data were collected using paper-based questionnaires administered during face-to-face interviews with caregivers. Caregivers served as the sole respondents, providing information on both their own status and that of the PwD. (line 161-163)

(2) The revised text specifies that caregivers completed one integrated questionnaire, comprising sociodemographic/clinical sections and five standardized instruments.

All information was obtained from informal caregivers, covering two primary domains. The first domain comprised sociodemographic and clinical details of PwD, including age, gender, education level (1= ≤6 years; 2= 7–9 years; 3= ≥10 years), type of dementia, years since diagnosis, number of children, ADL, and residence. The second domain encompassed caregivers’ own characteristics: age, gender, education level, relationship with PwD (1= children; 2= partner; 3= other), cohabitation status (1= yes; 2= no), length of caregiving, monthly income (CNY) (1= ≤2000; 2= 2000–5000; 3= ≥5000), and physical health. Physical health was assessed with the single-item question, “How do you rate your general health?”, rated on a 5-point scale from very poor to very good. For analysis, responses were dichotomized as impaired (1= very poor, poor, fair) and good (2= good, very good). Additionally, caregivers completed five instruments assessing quality of care, perceived overload, quality of the pre-illness relationship with PwD, depressive symptoms, and social support. (line 184-196)

17. **Line 160-162: please address the sentence structure as it is not clear what authors intend to convey.**

In accordance with the Editor's suggestion, the revisions have been incorporated. Please see Response to Editor Answer No.12. (line 165-166 in the revised manuscript)

18. **Line 162: does "The questionnaires were distributed and collected immediately" imply that "Participants were expected to complete questionnaires immediately upon distribution."**

Revised. Thank you for pointing out the potential ambiguity in this phrasing. We have revised the sentence to provide a clearer description of the procedure.

The questionnaires were completed and collected on-site. (line 166)

19. **Line 167 and 170: while authors reference studies which applied LPA [and indicate sample size calculation [including references] for their respective studies] the reviewer is not convinced that these studies could / should be referenced to corroborate authors’ statements regarding their sample size calculations. Authors are encouraged to preferably source, and reference, (pure) statistics-related publications addressing calculations of sample size (applicable to authors’ study).**

Thank you for your suggestion. We have removed the LPA study references and now justify sample size using G*Power [25] and one statistics-related publication [26].

An a priori sample size calculation was performed using G*Power 3.1.9.7. With a two-tailed, a medium effect size (f2=0.30), a power of 0.95, a statistical level α=0.05 [25], and 20 predictors, the minimum required sample size was 120. To ensure robust model fit, stable parameter estimation, and adequate power for subgroup comparisons in LPA, we targeted a minimum of 200 participants [26], consistent with methodological recommendations for person-centered approaches. (line 171-176)

[25]Cohen J, Cohen P, West SG. Applied Multiple Regression/Correlation Analysis for the Behavioral Sciences, third ed. New Jersey: Routledge; 2003

[26]Nylund, K. L., Asparouhov, T., & Muthén, B. O. (2007). Deciding on the Number of Classes in Latent Class Analysis and Growth Mixture Modeling: A Monte Carlo Simulation Study. Structural Equation Modeling: A Multidisciplinary Journal, 14(4), 535–569. <https://doi.org/10.1080/10705510701575396>

20. **Line 180: does Lau, et al. have a reference?**

Revised. Reference [18] is indeed the study by Lau et al. in which the Chinese version of the ECS was translated and validated.

The QoC was measured using the Exemplary Care Scale (ECS) , which was translated into Chinese by Lau, et al [18]. (line 205-206)

21. **Line 180: the sentence structure should read "ESC was completed …."**

Revised. (line 206)

22. **Line 191: the sentence structure should read "Relationship Rewards Scale (RRS) was used to …". Furthermore, a) the reviewer was unable to source a "Relationship Rewards Scale" and b) use of reference 15 should perhaps be reconsidered as a reference providing the reader to access to RRS.**

Revised. Thank you for pointing out the issues regarding verb tense and the accessibility of the scale. We have corrected the sentence to the past tense (line 212). Regarding the source of the scale, your comment is very pertinent. The specific items of the scale are indeed fully listed on page 219, line 36-45, of reference 15.

**四、Results**

23. **Line 259: a) "Caregivers was predominantly were female …" should read "Caregivers were predominantly female …" b) please refrain from starting a sentence with a number / percentage. Line 259 could possibly read "Caregivers were predominantly female (64.8%) and 75.6% of the caregivers lived with PwD."**

Revised. Caregivers were predominantly female (64.8%) and 75.6% lived with PwD. (line 267-268)

24. **Table 1: a) it is suggested that authors consider providing a separate table with results based on the different questionnaires caregivers completed. It is debatable whether affordability of living expenses; physical health, depression, quality of pre-illness relationship, perceived overload and social support could / should be considered as "Characteristics" of caregivers. In 2.2.2. authors refer to these as "independent variables" [as reported in line 296-300] b) consider providing a footnote indicating what "Other" relationships with PwD were.**

We thank the reviewer. The manuscript has been revised as suggested: (1) Table 1 has been split, the variables (quality of pre-illness relationship, perceived overload and social support) moved to a new Table 2 (line 273); (2) a footnote clarifying the “Other” relationship category has been added. (line 272)

25. **Line 264: revise "… caregivers of PwD were shown …" to read "… caregivers of PwD are shown …"**

Revised. (line 276)

26. **Table 4: "LPAa" is not explained as a footnote to Table 4.**

Revised. Thank you for your thorough review. You are absolutely correct that the superscript "LPAa" in Table 4 lacks a corresponding footnote explanation. Upon verification, "LPAa" carries no specific meaning, and retaining it might cause confusion. Therefore, we have removed "LPAa" to ensure the table is concise and clear.

27. **Figure 1: the only legend regarding colours used corresponds to blue "Low QoC".**

Revised. (line 299)

28. **Table 5: the heading of the table indicates the test performed rather than what is reported in Table 5 as a result of the test performed.**

Revised. Thank you for your comment. We have revised the title of Table 5 (now Table 6) to provide a direct description of the results.

Table 6 Results of multinomial logistic regressions predicting profile membership (R3STEP). (line 313)

29. **Table 7: what is the intention of "ROC" in the table [given the table heading]?**

Revised. Thank you for your comment. The original Table 7 is now Table 8. To more clearly reflect the relationship between the table content and the ROC analysis results, we have revised the title to: Table 8 Negative and positive groups classified by the optimal ROC cut-off. (line 332)

**五、Discussion**

30. **Line 319: "Based on Extended Stress Model, …" should read "Based on the Extended Stress Model, …"**

Thank you for your comment on Line 319. In response to Reviewer 6’s comment, I have rewritten this section.The relevant section no longer appears in the manuscript.

31. **Line 321: "… caregiver resources predicted …" should read "… caregiver resources predict ..."**

Thank you for your comment on Line 321. In response to Reviewer 6’s comment, I have rewritten this section.The relevant section no longer appears in the manuscript.

32. **Line 328-336: seems these lines repeat what authors already stated in the Result section and could be better utilized to elicit discussion.**

Revised.

Thank you for your comment. In line with Reviewer 6’s suggestion, we have revised this section accordingly. Please refer to our response to Reviewer 6, Answer No. 46, for details. (line 331-366 in the revised manuscript)

33. **Line 337-338: this sentence is incomplete.**

Thank you for your comment on Line 337-338. In response to Reviewer 6’s comment, I have rewritten this section.The relevant section no longer appears in the manuscript.

34. **Line 342: provide reference corroborating the statement.**

We thank the reviewer for this comment. As suggested, reference [16] has now been provided to support the statement on line .(line 358)

[16] McClendon, M. J., & Smyth, K. A. (2013). Quality of informal care for persons with dementia: dimensions and correlates. Aging & mental health, 17(8), 1003–1015. https://doi.org/10.1080/13607863.2013.805400.

35. **Line 343: it is not clear what authors imply with "… except caregivers’ quality of life"? It seems authors emphasize that the caregiver’s quality of care is impacted but not the person’s quality of life perse?**

In accordance with the Editor's suggestion, the revisions have been incorporated. Please see Response to Editor Answer No.18 (line 357-360 in the revised manuscript).

36. **Line 345: consider revising the word "decided". It seems authors are stating that the three QoC profiles identified during the study pivoted on / revolved around / was determined by the list of aspects mentioned in the sentence?**

Thank you for your comment on Line 345. I agree the original sentence was unclear. In response to Reviewer 6’s comment that the Discussion should not present new results, I have rewritten this section.The relevant section no longer appears in the manuscript.

37. **Line 381: it is assumed that "… consisting other studies …" should read "… consistent with other studies …"? Irrespective, the sentence does not make sense.**

Thank you for your comment. We agree the original sentence was unclear. Upon review, we found that its content was redundant with points already made elsewhere in the Discussion. To improve clarity and conciseness, we have deleted this sentence.

38. **Line 409-411: this sentence seem more fitting as a conclusion instead of a discussion point.**

Thank you for your comment. We agree the sentence was more fitting for a conclusion. To maintain the focus of the Discussion on interpretation rather than summary, we have deleted this sentence.

**Response to Reviewer 4:**

1. **Thank you for your paper some comments to consider People with dementia rather than patients should be used.**

Thank you for this important suggestion. We will adopt person-first language by replacing "patients with dementia" with "people with dementia (PwD)" throughout the manuscript, using the abbreviation "PwD" after its first occurrence.

2. **Background-Whilst this is well written it seems too long and would benefit from some editing.**

Thank you for your comment. We have now condensed it to improve focus and readability.

3. **Line 71 pleasant activities ?**

Thank you for your feedback regarding the precision of the terminology. We have revised the originally vague term "pleasant activities" to the more specific and academic expression "enjoyable social engagement". (line 72-73 )

4. **Capital letter mid sentence line 101**

Thank you for pointing this out. The original sentence at Line 101 has been rewritten in response to the Editor’s comment (see Editorial Comment 11), and the capitalization issue no longer exists. (line 101-104 in the revised manuscript).

5. **Lines 360-362 some grammatical issues**

Revised.

We thank the reviewer for pointing out the grammatical issues in the sentence. We have revised the text to enhance its clarity and conciseness.

This finding emphasizes that pre-illness relationship quality, while not modifiable, is a critical factor. It suggests prioritizing interventions for caregivers with poorer pre-existing relationships, as they may require more intensive support to achieve exemplary care. (line 388-391 )

6. **Generally just check grammar throughout**

Thank you for this important feedback. We will conduct a thorough grammar check and language polish throughout the entire manuscript to improve its clarity and readability.

**Response to Reviewer 5:**

**First, I would like to congratulate the authors on their excellent work and on giving me the opportunity to review it. The topic addressed, the quality of care, is relevant, and the authors' approach provides information that may be useful to the scientific community. The effort made by the authors to convey their results is evident.**

**As a result of my review, I have the following suggestions regarding your manuscript. My suggestions are divided into general suggestions, which affect the manuscript, and specific suggestions, which are described by line number and section of the manuscript to facilitate their location.**

**I hope to have the opportunity to review your manuscript again with the suggested corrections.**

Thank you for recognizing the value of this research and for your valuable feedback. We will revise the manuscript comprehensively in accordance with all your suggestions and look forward to your further evaluation.

**Response to Reviewer 6:**

**The comments are attached to the document. They are clear and direct and the author can be able to action the issues raised.**

Thank you for your review and for providing clear and actionable feedback in the attached document. We have received the comments and will address each point thoroughly in our revision. We appreciate the guidance and look forward to submitting an improved manuscript.

**First, I would like to congratulate the authors on their excellent work and on giving me the opportunity to review it. The topic addressed, the quality of care, is relevant, and the authors' approach provides information that may be useful to the scientific community. The effort made by the authors to convey their results is evident.**

**As a result of my review, I have the following suggestions regarding your manuscript. My suggestions are divided into general suggestions, which affect the manuscript, and specific suggestions, which are described by line number and section of the manuscript to facilitate their location.**

**I hope to have the opportunity to review your manuscript again with the suggested corrections.**

**General suggestions regarding the manuscript:**

**The authors include many phrases in a single paragraph, making the length of the paragraph difficult to read and to maintain the idea of what was being read. To make the manuscript easier to read, it would be advisable to divide the long paragraphs using new paragraphs.**

**Within the current paragraphs, the sentences often stand alone and are not adequately interconnected, making it difficult to establish a causal link between one sentence and the next. The authors should try to use words at the beginning of sentences that connect the previous sentence to the current one.**

**The same situation occurs between paragraphs. Authors should try to interconnect paragraphs with bridging words such as "on the other hand," "likewise," "along these lines," and "however" to make them easier to read.**

**Finally, the language used is sometimes very colloquial, suggesting that some words be replaced with more technical or neutral ones. Likewise, statements are sometimes made that are not supported by the data provided or the bibliography, and it is recommended that these points be reviewed.**

Thank you for the encouraging review and valuable suggestions. We have thoroughly revised the manuscript to address all general points regarding paragraph structure, logical flow, language formality, and support for claims.

**Specific suggestions regarding the manuscript:**

**一、Introduction:**

1. **Line 53, first paragraph of the Introduction section: the phrase "that results in" should perhaps be modified to use a term such as "is associated with."**

Revised. (line 54)

2. **Line 54: authors should clarify whether the data "152 million in 2050" refers to the global level or to China.**

In accordance with the Editor's suggestion, the revisions have been incorporated. Please see Response to Editor Answer No.4 (line 55 in the revised manuscript).

3. **Line 56: the phrase "In China, the number of PwD accounts for about a quarter of the total number, ranking first in the world [3,4]" could be improved. An alternative phrase would be: "Currently, in terms of global prevalence, one in four PwD reside in China, which ranks first in the world in terms of number of cases."**

Revised.

Currently, one in four PwD reside in China, which ranks first worldwide in terms of number of cases. (line 56-57)

4. **Line 59: The sentence "Informal caregivers are thought to be more familiar with their relative with dementia than formal caregivers, and often provide care that is more in line with the relative's preferences and values" could probably be improved by the authors, using, for example, a sentence such as "This care is based both on the cultural tradition of caring for our elders and on a greater knowledge of their preferences and values than formal caregivers may have or provide."**

Revised. (line 59-61)

5. **Line 64: Facilitate the connection between separate sentences, using, for example, words such as "In this sense, some studies..." .**

Revised. (line 64)

6. **Line 65: The authors could improve the wording of the phrase "Indirectly shortening survival," for example, by describing this situation in terms such as "situations that, directly or indirectly, end up affecting their life expectancy."**

Revised. Thank you for your specific suggestions for rewording.

In this sense, some studies have shown that low QoC adversely affects the health of PwD, increasing the risk of recurrent hospitalizations and institutionalization, outcomes that can ultimately affect their life expectancy [8,9]. (line 64-66)

7. **Line 73: The authors could improve the wording of the phrase "These dimensions ignored CRs' feelings and wishes, and failing to reflect the meaning of quality home care." Among the various options for making this improvement, the following example is provided: "These dimensions are not sensitive to the feelings and wishes of CRs and, therefore, do not reflect all the aspects that quality care for PwD living in the community should consider."**

Revised.

We sincerely appreciate the reviewer not only for identifying the issue but also for providing a clear and specific example for improvement. Accordingly, we have revised the sentence based on your suggestion.

However, these dimensions are not sensitive to the feelings and wishes of CRs and, therefore, fail to reflect all the aspects that quality care for PwD living in the community should consider. (line 75-77)

8. **Line 80: The phrases "This approach conveys to CRs the affirming message that they are ‘loved, respected, and worthy of special attention.’ The message helps maintain the dignity and integrity of CRs, reflecting the concept of ‘person-centered’ care" are redundant with respect to statements in previous phrases and could be simplified, for example, by expressing in the text: "This approach conveys to care recipients the message that they are loved, respected, and worthy of special attention, helping to maintain their dignity and integrity, and holistically reflecting the concept of person-centered care."**

Revised. We have revised the sentence based on your suggestion.

This approach conveys to care recipients the message that they are loved, respected, and worthy of special attention, helping to maintain their dignity and integrity, and holistically reflecting the concept of person-centered care. (line 82-85)

9. **Line 82: The authors are advised to divide the paragraph at this point, as its current length makes it difficult to read and understand. The new paragraph should connect to the last sentence of the previous paragraph, using words such as " In this line, and based on the conceptual framework of exemplary care."**

Revised.

We have revised the manuscript in line with your suggestion: the original paragraph (line 82) has been split at line 86 and now opens with your recommended transitional phrase to ensure a logical flow.

10. **Line 95: The authors should improve the connection between phrases and interconnect the messages that these phrases convey, using phrases such as, for example, "Although much research focuses on the total ECS scale score to assess overall quality of life and the factors that influence it [16-20], this five-variable approach fails to capture individual differences and does not allow for the observation of heterogeneity among different subgroups.**

Revised.

We sincerely appreciate the reviewer not only for identifying the issue but also for providing a clear and specific example for improvement. Accordingly, we have revised the sentence based on your suggestion.

Although much research focuses on the total ECS score to assess overall quality of care and the factors that influence it [16-20], this variable-centered approach fails to capture individual differences and does not allow for the observation of heterogeneity across subgroups. (line 98-101)

11. **Line 104: Authors are suggested to insert a paragraph break at this point, as the current length hinders reading and overall comprehension. The new paragraph should connect to the last sentence of the previous paragraph, using words such as "Along the same lines, the latent profile analysis..." .**

We have split the paragraph at line 104 to improve readability as suggested. Regarding the transitional wording, because the specific analytical method (LPA) is now detailed in the Statistics section per another Reviewer 3's comment.

12. **Line 113: We recommend modifying the verb forms so as not to take the result of the study for granted. For example, using the following words: "The results of this study could provide important empirical evidence for the subsequent development of precise intervention strategies for different subgroups of caregivers."**

Revised.

The results of this study could provide important empirical evidence for the subsequent development of precise intervention strategies for different subgroups of caregivers. (line 137-139)

13. **Line 116: The authors should improve the connection between paragraphs. Using words such as "On the other hand, although the heterogeneity analysis..."**

Revised. (line 106)

14. **Line 118: This phrase might better express what is intended if it is stated in the future tense: "To address this practical challenge, we propose to investigate in greater depth through this study the optimal cutoff value of ECS using ROC analysis, using the results of the LPA classification as a criterion."**

**The authors could improve the wording and include part of the following paragraph. For example: "This approach would allow clinicians and community professionals to quickly identify high-risk groups and establish action strategies, using only the total score as a reference. The proposed simplification of the scoring process represents a methodological innovation that preserves the advantage of the LPA in identifying heterogeneity and improves its clinical applicability, while also providing empirical evidence for understanding the causes of this heterogeneity."**

Revised.

We sincerely appreciate the reviewer not only for identifying the issue but also for providing a clear and specific example for improvement. Accordingly, we have revised the sentence based on your suggestion.

To address this practical challenge, we propose to investigate in greater depth through this study the optimal cut-off value of ECS using Receiver operating characteristic (ROC) analysis. This approach would allow clinicians and community professionals to quickly identify high-risk groups and establish action strategies, using only the total score as a reference. The proposed simplification of the scoring process represents a methodological innovation that preserves the advantage of the LPA in identifying heterogeneity and improves its clinical applicability, while also providing empirical evidence for understanding the causes of this heterogeneity. (line 107-115)

15. **Line 124: The hypotheses should come before the objectives and be placed on line 124. The current wording of the paragraph addressing the hypotheses could be improved. An attempt should also be made to interconnect the different paragraphs to make them easier to read. The following wording is proposed as an example to the authors, which they can modify according to their criteria:**

**"In this context, our hypotheses are: (1) the quality of informal care could be categorized into different latent profiles according to the ECS; (2) the quality of informal care provided by caregivers varies depending on individual characteristics, stressors, and social support. (3) On this basis, and through ROC analysis, it may be possible to establish an optimal cutoff value with high sensitivity and specificity that allows for the identification of different levels of care quality and, specifically, the identification of low-quality care."**

**The authors should also move and adapt some subsequent phrases included in the objectives paragraph and place them in a new paragraph after the hypothesis paragraph:**

**"These results would have two practical implications. First, they would provide a scientific basis for developing precise interventions tailored to different subgroups. Second, they could significantly improve the assessment of quality of care and, with it, efficiency."**

**Thus, from line 124 onwards, the development of the hypotheses and objectives should follow a chronological order, for example:**

**"In this context, our hypotheses are: (1) the quality of informal care could be categorized into different latent profiles according to the ECS; (2) the quality of informal care provided by caregivers varies depending on individual characteristics, stressors, and social support. (3) On this basis, and through ROC analysis, it may be possible to establish an optimal cutoff value with high sensitivity and specificity that allows for the identification of different levels of care quality and, specifically, the identification of low-quality care."**

**"These results would have two practical implications. First, they would provide a scientific basis for developing targeted interventions tailored to different subgroups. Second, they could significantly improve the assessment of care quality and, thereby, efficiency."**

**"To respond to these hypotheses, four objectives were set out in this study (perhaps the authors should set out four objectives instead of three, with objective 4 being: "and (4) to propose rapid screening criteria that enable health and social organizations to quickly identify high-risk individuals." I also suggest modifying the wording of objective 3 to include the areas of application where this objective would be relevant: "(3) identify categorization thresholds that allow for application in clinical and community practice through ROC analysis."**

**Interconnect paragraphs. For example: "To respond to these hypotheses..." .**

We sincerely appreciate the reviewer's exceptionally comprehensive and constructive feedback. Your suggestions on restructuring the sections concerning the research hypotheses, practical implications, and study objectives have greatly enhanced the logical flow of the concluding part of our introduction. We have thoroughly revised and restructured the manuscript from line 124 onwards in strict accordance with all your recommendations.

In this context, our hypotheses are: (1) the quality of informal care could be categorized into different latent profiles according to the ECS; (2) the quality of informal care provided by caregivers varies depending on individual characteristics, stressors, quality of life, and social support. (3) On this basis, and through ROC analysis, it may be possible to establish an optimal cutoff value with high sensitivity and specificity that allows for the identification of different levels of care quality and, specifically, the identification of low-quality care.

These results would have two practical implications. First, they would provide a scientific basis for developing targeted interventions tailored to different subgroups. Second, they could significantly improve the assessment of care quality and, thereby, efficiency.

To respond to these hypotheses, we set four objectives: (1) to identify potential subgroups of the quality of care among informal caregivers of PwD,; (2) to clarify the factors associated with these subgroups; (3) to identify categorization thresholds for the ECS that can be applied in clinical and community practice; and (4) to propose rapid screening criteria that enable health and social organizations to quickly identify high-risk individuals. (line 116-132)

**2. Materials and methods**

**2.1 Design and participants**

16. **Line 143: The authors should expand on the description of the type of study conducted.**

Thank you for your suggestion. To provide a more complete description of the study type, we have revised the text: This cross-sectional study was conducted between July 15, 2023, and July 14, 2024, in Wuhan, Hubei Province, China. (line 143-144)

17. **Line 146: The authors should specify the city, province, and country where the study was conducted.**

Thank you for your correction. We have explicitly added the specific location where the study was conducted.

This cross-sectional study was conducted between July 15, 2023, and July 14, 2024, in Wuhan, Hubei Province, China. (line 143-144)

18. **Line 149: To make the manuscript easier to read, it would be advisable to divide the long paragraphs with full stops. We suggest dividing this paragraph into this line.**

Thank you for the suggestion to improve readability. We have divided the indicated long paragraph into shorter ones as recommended. (line 150)

19. **Line 157: To make the manuscript easier to read, it would be advisable to divide the long paragraphs with full stops. We suggest dividing this paragraph into this line.**

Thank you for the suggestion to improve readability. We have divided the indicated long paragraph into shorter ones as recommended. (line 160)

20. **Line 165: To make the manuscript easier to read, it would be advisable to divide the long paragraphs with full stops. We suggest dividing this paragraph into this line.**

Thank you for the suggestion to improve readability. We have divided the indicated long paragraph into shorter ones as recommended. (line 170)

21. **Line 165: Regarding the new independent paragraph created, "For multinomial logistic regression, the sample size"... it is suggested that the authors improve the wording and interconnect paragraphs. Perhaps the authors should rewrite this paragraph to increase the clarity of what is being presented and maintain a logical sequence. Reposition the bibliography if necessary. As an example of wording, which can be modified by the authors at their discretion: "To meet the objectives of the study, it was taken into account that, within a potential categories model, a sample size of no less than 200 people was necessary [25]. Initially, 248 participants were recruited. However, 35 were excluded due to incomplete responses or other reasons, resulting in 213 valid participants included in the final analysis (response rate of 85.89%). On the other hand, for multinomial logistic regression, the sample size should be at least 5-10 times the number of independent variables [24]. With 20 variables included in this study and considering a potential rate of 20% invalid questionnaires, a minimum of 120 participants was initially required.**

Thank you for the constructive suggestions on paragraph structure and wording. We have fully rewritten the section to improve logical flow, following the sequence you exemplified. This revision, the specifics of which are also outlined in our response to Editor Answer No.12, enhances the overall clarity. (line 171-176 in the revised manuscript)

**2.2 Variables and measures**

**2.2.1 Dependent variable**

22. **Line 180: The wording of this sentence could be improved, for example: "The ECS is completed by informal caregivers and consists of 11 items that fall into two dimensions: Provide (items 1-5) and Respect (items 6-11)."**

Thank you for your valuable suggestions regarding sentence structure and clarity. We fully adopted your proposed optimization approach. Meanwhile, to address Reviewer 3’s correction regarding verb tense, we have split the original sentence into two parts, each using the appropriate tense. The revised text now reads: The ECS was completed by the informal caregivers. It consists of 11 items that fall into two dimensions: Provide (items 1-5) and Respect (items 6-11). (line 206-208)

**2.2.2 Independent variable**

23. **Authors should describe the criteria used for the variables listed in Table 1 that may be analyzed in this study, including the levels, scale, range, or method used for classification. Specifically, the levels of education, the criteria used to define cohabitation with the person with dementia, and the criteria used to establish whether caregivers of the person with dementia have the capacity to pay for living expenses.**

Thank you for your valuable comments regarding the clarification of variables in our study. We fully agree that a precise description of measurement and classification criteria is essential. Accordingly, we have revised the "2.5 Measures" section to provide detailed operational definitions for the levels of education, the criteria used to define cohabitation with the person with dementia, and the criteria used to establish whether caregivers of the person with dementia have the capacity to pay for living expenses.

All information was obtained from informal caregivers, covering two primary domains. The first domain comprised sociodemographic and clinical details of PwD, including age, gender, education level (1= ≤6 years; 2= 7–9 years; 3= ≥10 years), type of dementia, years since diagnosis, number of children, ADL, and residence. The second domain encompassed caregivers’ own characteristics: age, gender, education level, relationship with PwD (1= children; 2= partner; 3= other), cohabitation status (1= yes; 2= no), length of caregiving, monthly income (CNY) (1= ≤2000; 2= 2000–5000; 3= ≥5000), and physical health. (line 184-191)

24. **On the other hand, within the variable of cohabitation with the person with dementia, the authors establish as an inclusion criterion "care for more than 4 hours each day," without requiring cohabitation in the same home. This may generate a bias, as it includes people who live with the person with dementia 24 hours a day and those who only do so for onesixth of the day, a bias that should be considered both in the results and in the limitations of study. The authors should justify how a person who does not live with the patient can be their caregiver.**

We sincerely thank the reviewer for raising this critical and insightful point. You correctly identified that using ">4 hours of care per day" as a core inclusion criterion without requiring cohabitation may merge caregiver groups with vastly different contexts and intensities of care—a potential bias that warrants serious consideration.

In response to the fundamental question of how a non-cohabiting individual can serve as a primary caregiver, we have cited the authoritative review by Happ et al. in the Journal of Gerontological Nursing [1]. This article systematically clarifies that the contemporary definition of informal caregivers for persons living with dementia centers on the provision of unpaid assistance, rather than physical cohabitation, and that care activities can indeed be delivered without shared residence. This provides a solid conceptual foundation for our inclusion criteria.

Regarding the specific threshold of ">4 hours per day," we acknowledge that it follows operational conventions in the research field. We have referenced empirical studies that apply similar time criteria to define "primary caregivers," demonstrating that this is a common methodological practice to ensure that the sample comprises individuals with a substantive caregiving role.

(2) providing at least four hours per day on caregiving for no less than three months, thereby fulfilling the role of the primary caregiver [24]. (line 151-153)

Following your suggestion, we have added the following paragraph to the "Discussion- Limitations" section:

Fifth, our sample included both co-residing and non-co-residing caregivers, which may entail distinct caregiving contexts and challenges. Stratifying by residential status in future research would enable a more nuanced understanding of the unique experiences and support needs of each subgroup. (line 443-447)

[1] Happ MB, Moss KO, Dabbs AD, Narby E, Song M-K. Defining Informal Caregiving and Caregivers for Persons Living With Dementia. Journal of Gerontological Nursing. 2024;50(12):41-45. doi:10.3928/00989134-20241118-05
25. **Perhaps a new variable should be established:**

**Caregiver's relationship with the dementia patient: The type of relationship between the caregiver and the dementia patient was evaluated, establishing the following groups of caregivers: 1: children; 2: partner; and 3: other types of caregivers. Regarding the categories currently included in the manuscript:**

Revised.

relationship with PwD (1= children; 2= partner; 3= other) (line 189)

26. **Quality of pre-illness relationship:**

**Line 193: The first word is lowercase. It should read "Caregivers." The authors should define the intermediate values (2, 3).**

Revised.

We thank the reviewer for the careful correction. We have accordingly revised the sentence based on your two specific points.

Caregivers assessed the frequency (1=never, 2=sometimes, 3=often, 4=always) at which they perceived pre-illness interpersonal relationships with CRs as rewarding, with a higher score indicating a greater quality of pre-illness relationship. (line 213-216)

27. **Activities of daily living (ADL):**

**Line 198: I have a question about the last sentence in this section: "The scale was completed by informal caregivers based on the condition of PwD." The authors imply that the primary caregivers were the ones responsible for completing the Barthel index. This point should be clarified, explaining whether the interviewers asked family members about the situation of the person with dementia performing basic activities of daily living and whether the interviewers filled out the questionnaire. Or the questionnaire was selfcompleted by the caregivers, i.e., family members were given a booklet with the Barthel Index questions, and the caregivers completed the questionnaire independently, according to the situation of their family member with dementia.**

**On another note, I believe it is necessary to open a debate on this point. The Barthel Index was initially designed to assess functional capacity and has numerous classifications. The authors reference that they use the classification proposed by Shah in 1989, which is an adaptation of the Barthel Index for a specific setting, but the classification they propose is not one of the most widely used. Furthermore, since then, numerous studies have been conducted on the usefulness of the Barthel Index, proposing various cut-off points for levels of functional dependence. Multiple studies have also been conducted associating the different levels of functional dependence of the Barthel Index with mortality. Others associate each item of the Barthel Index with mortality. All of this has been done with the aim of analyzing the association between the ability to perform basic activities of daily living and life expectancy. As a result of all these studies, there is a certain consensus that below a Barthel index of 40, there is a significant increase in the risk of mortality, the risk of hospitalization, the risk of complications during hospitalization, and the risk of complications upon discharge.**

**Although no global consensus has been published on the levels used in population studies, it seems logical to suggest that the most up-to-date data with the strongest evidence should be used to facilitate comparisons between studies and the performance of meta-analyses. Therefore, although the classic 5-level classification remains in force (total dependence (Barthel <20); severe dependence (Barthel 20-35); moderate dependence (Barthel 40-60), mild dependence (Barthel 65-95), and independence (Barthel 100 (from 90 according to other authors)), it is advisable to collect data on the risk of mortality and complications associated with functional dependence and use a 4 level classification: severe dependence (Barthel 0-35); moderate dependence (Barthel 40-60), mild dependence (Barthel 65-95); and independence (Barthel 100). Therefore, I suggest that the authors consider reclassifying their patients according to this scale and performing the analyses based on it. This modification can be justified in the manuscript by establishing that these levels are those that have been shown to be associated with the risk of mortality and the risk of complications. On the other hand, by de facto dichotomizing the ADL variable (the number of patients with moderate dependence in their sample is small), differences may be observed that were not detected with the previous classification.**

Thank you for the suggestions.

We have clarified that the Barthel Index was interviewer-administered based on caregiver proxy report. We have adopted the recommended 4-level classification and re-analyzed the data accordingly.

Based on the BI score, patients' ADL is classified into four levels: independence (100), mild dependence (65–95), moderate dependence (40–60), and severe dependence (0–35) [28]. Data were collected via face-to-face interviews conducted by trained researchers with family caregivers. (line 200-203)

28. **Perceived Overload:**

**Line 203: The wording could be improved. For example: "To assess the perceived overload of caregivers, the overload scale, consisting of 4 items, was used." In addition, the authors should define in this section each value that an item can take and not just values 1 and 4.**

Revised.

We thank the reviewer for the careful correction. We have accordingly revised the sentence.

Caregiver perceived overload was assessed using the 4-item Overload Scale. Items were rated on a 4-point scale (1=not at all, 2=somewhat, 3=quite a bit, 4=completely), with total scores ranging from 4 to 16, with higher scores indicating a higher perceived overload [30]. (line 219-222)

**Physical health:**

29. **Line 209: Fair health is not the same as poor health. If both items are included to create a binary variable, the categories could be defined, for example, as "impaired" (1 = very poor, poor, fair) and "good" (2 = good, very good).**

Revised. (line 193)

**2.3 Statistics analysis**

30. **Line 229: The authors should improve the wording of the first phrase.**

Revised.

We thank you for the suggestion. We have refined the phrasing of the sentence.

We conducted four statistical analyses. First, descriptive statistics were used to summarize participant characteristics. (line 239-240)

**3. Results**

**General considerations regarding the Results section:**

31. **The authors have a large amount of data, which they include in Table 1, but do not present in the text of the manuscript. I do not know if this is because they are reserving this data for another study on this cohort. If this is not the case, perhaps its presentation in this manuscript would provide relevant information on the results, which could be very useful for other researchers. Information that, when properly related in the text, could not only complement that provided by reading the table, but also allow the interrelationships between the variables in the table to be established. Among other possible aspects that could be addressed are:**

Thank you for this suggestion. We have added a paragraph in the Results section. A full analysis of all variable interrelationships is provided in Appendix 1 for comprehensive reference.

After univariate analyses identified significant variables (Appendix), multinomial logistic regression using the R3STEP procedure in Mplus was conducted to examine their effects on profile membership, with the moderate QoC profile as the reference. Results are presented in Table 6. (line 304-307)

32. **There is no mention that most people with dementia (54.9%) were male. Globally, dementia is more prevalent in women. This is a distinguishing feature of your study. Perhaps it should be mentioned in this section and in the Discussion section.**

Thank you for highlighting this notable characteristic of our sample. We have added a statement in the Results section to explicitly mention: Notably, 54.9% of the PwD were male. (line 265)

We agree this contrasts with global epidemiological trends. As the primary focus of this paper is on the quality of care profiles and their correlates rather than on demographic epidemiology, we have chosen to highlight this distinctive sample feature in the Results for transparency, but without further elaboration in the Discussion to maintain focus and brevity.

33. **There is also no mention of the fact that while people with dementia typically had a low level of education (52.6%), caregivers typically had a high school education (44.1%). This data shows that the social ladder that allows one generation to surpass the previous one is active.**

We appreciate the reviewer’s note on the intergenerational educational difference. This is indeed an interesting observation.

34. **The couple lives with the person with dementia, but among the children and other nonchildren, it might be useful to know what percentage of each group lived with the person with dementia.**

Thank you for this suggestion. The detailed cohabitation rates by caregiver relationship type (spouse, child, other) are indeed available in Appendix 2 (Table 1). Since the main focus of this study is on care quality outcomes, we did not elaborate on these descriptive differences in the main text to keep the narrative concise. We agree that these patterns are informative for understanding the sample composition and have now highlighted them in the appendix accordingly.

35. **It would also be useful to know, among the caregivers who did not live with the person with dementia, whether they had been caring for this person for less than one year, one to five years, or more than five years. Similarly, it would be useful to know whether the time since the diagnosis of dementia was less than one year, one to five years, or more than five years. Perhaps these part-time caregivers have been caring for the person with dementia for less than a year or are caring for people with a recent diagnosis of dementia, data that may influence the overall analysis of the results.**

Thank you for this valuable suggestion. The distributions of caregiving duration and time since diagnosis by cohabitation status are already presented in Appendix 2 (Tables 2 and 3). As shown, caregiving duration was significantly associated with living arrangements, whereas time since diagnosis was not. Given that the primary focus of this study is on care quality outcomes, we chose to keep the main text focused and did not elaborate on these descriptive differences. The appendix provides full details for interested readers.

36. **The average age of caregivers is high. This is probably due to the age of the partners of people with dementia. This aspect should be clarified in this section by the authors, specifying the average age for each group proposed in the definition of variables and in the text of this section.**

Thank you for raising this point. The average ages for each caregiver group (spouse, child, other) are now detailed in Appendix 2 table 4.

Caregivers’ mean age differed by their relationship with PwD: spouses 60.66±15.15 years, adult children 57.50±13.85 years, and others 60.93±13.69 years (Appendix). (line 265-267)

37. **It would provide information for the interpretation of the results if the authors analyzed whether there is an association between the level of education of caregivers of people with dementia and the level of difficulty in paying for the living expenses of the person with dementia. If it is observed that a lower level of education is associated with greater difficulty in meeting these expenses, a new line of action could be proposed by social services for this most vulnerable group.**

Thank you for this valuable suggestion. We have analyzed the association between caregiver education level and financial strain. Results show that lower education is significantly associated with greater financial difficulty (see Appendix 2, Table 5). We have now discussed the implications for targeted social service interventions in the Discussion section.

Low education and rural residence were strong predictors of low income [45], thereby indirectly increasing the risk of poor QoC. Notably, lower income was also significantly associated with higher depressive symptoms [46], suggesting that financial strain undermines psychological well-being and further compounds care quality risks. These findings therefore identify caregivers with low income, low education, rural residence, and elevated depressive symptoms as a multiple disadvantaged subgroup, underscoring the need for integrated economic, mental health, and educational support from social services. (line 373-380)

38. **It would also be interesting to know whether women had greater difficulty in meeting the living expenses of the person with dementia than men. And whether this was associated with their level of education, physical health, or the presence of depression. If confirmed, it would also suggest a course of action by social services for this most vulnerable group.**

Thank you for this insightful suggestion. We have analyzed whether female caregivers experienced greater financial strain, and whether this was associated with education, physical health, or depression.Results are presented in Appendix 2, Tables 5, 6, 7, and 8. No significant association was found between gender and financial strain , nor between self--rated physical health and financial strain. However, lower education level, depressive symptoms were significantly associated with greater financial difficulty. We have now discussed the implications for targeted social service interventions in the Discussion section. (line 373-380)

39. **On the other hand, the environment, rural or urban, may have implied greater or lesser difficulty in meeting the costs of care.**

Thank you for this relevant point. We will include residence (urban/rural) in our analysis of factors associated with financial strain in caregiving. Results are presented in Appendix 2, Table 9. PwD’s residence in urban/town areas was significantly associated with lower monthly income. We have now discussed the implications for targeted social service interventions in the Discussion section. (line 373-380)

40. **Finally, all these results, in addition to being described in the text, could be included in their numerical values in a new table. This could encompass other analyses that, in line with the proposed approach, could be incorporated by the authors.**

Thank you for the suggestion. We will create a new table consolidating all key results with their numerical values for clarity.

Thank you for the suggestion. We agree that presenting the numerical values in a consolidated format would improve clarity. Accordingly, we have created a new table (Appendix 2) that summarizes all new results with their corresponding numerical values.

41. **Line 276: Table 2: The table legend should include a description of the acronyms so that the table can be read independently, without referring to the text of the manuscript.**

Thank you for this helpful suggestion to improve the table’s clarity. We have updated Table 2 (now Table 3) by adding a note that provides the full descriptions of all acronyms (AIC, BIC, aBIC, LMR, BLRT), ensuring the table can be understood independently of the main text. (line 288)

42. **Line 277: Table 3: The description of each profile should be included to avoid having to refer to the text of the manuscript.**

Thank you for this suggestion to improve table clarity. We have revised Table 3 (now Tabla 4) by adding an explanatory note. The note clarifies that the table assesses classification accuracy.

Note: Diagonal values (bolded) indicate correct classification probabilities. (line 289)

43. **Line 278: Table 4: The legend of the table should include a description of the acronyms so that the table can be read independently, without referring to the text of the manuscript.**

Revised. The original Table 4 is now numbered as Table 5. (line 290)

**3.4 ROC analysis results**

44. **Line 305: The first sentence of this paragraph should be placed within the Material and Methods section, subsection 2.2.1 Dependent variable.**

**When making this change, it would be advisable to restructure this paragraph. Perhaps with a sentence such as: "Using the standard described in the section on the dependent variable..." .**

Following the editor's suggestion, we have revised this paragraph to present the split-sample validation analysis. Please see Response to Editor Answer No.3 (line 316-326 in the revised manuscript).

45. **Line 312: Figure 2: To enable other authors to evaluate the results shown in Figure 2, it would be advisable to include the data table that SPSS, or the program used to produce it, provides when running the process. This table could be included as an appendix to the manuscript.**

We appreciate your insightful suggestion. The data supporting Figure 2 have been submitted as an online supplement and are now accessible to reviewers.

**4. Discussion**

**General comment:**

46. **Sometimes the sentences within a single paragraph are disconnected from each other, giving the impression of loose sentences. The authors should review this aspect and try to interconnect the end of one sentence with the beginning of the next. This can be applied to the entire manuscript. In this section, the connection between one paragraph and the next should also be improved. On the other hand, there is also a tendency to repeat results or to redescribing the methods used, rather than creating a Discussion about them. Likewise, in the first three paragraphs there are redundant sentences, repetitive with other words, or the same. The authors should synthesize these paragraphs. In addition, the beginning of the second paragraph of the Discussion section is like the first. The wording of this paragraph should be changed. On the other hand, percentages are added, which might be better moved to the Results section, stating here, for example, that most caregivers of people with dementia have a moderate quality of life, and a high percentage have a poor quality of life. Finally, the first and second paragraphs present a discussion of the results that is partially intermingled. Both paragraphs should be rewritten, attempting to go step by step in the discussion of the results they have.**

We thank the reviewer for the detailed guidance. We have rewritten the first two paragraphs of the Discussion to: (1) change the redundant opening, (2) remove specific percentages in favor of qualitative statements, and (3) clearly separate the general findings from the in-depth profile comparison, ensuring a logical, step-by-step discussion.

This study identified three distinct profiles of informal care quality among caregivers of PwD, confirming significant heterogeneity in the quality of informal care. The low QoC profile scored the lowest on items related to emotional aspects (e.g., Hobby, Gathering), reflecting a pattern of survival-oriented, passive care that addressed only basic life needs. Social engagement, emotional support, and respectful treatment were largely absent, indicating minimal attention to the PwD’s dignity, preferences, or psychosocial well-being [18]. The low QoC profile was driven by a convergence of caregiver adversities: economic strain, high overload, and inadequate support, highlighting structural determinants of poor outcomes. This profile may be particularly common among caregivers of severely dependent PwD, those with prolonged care duration, or those struggling with limited financial resources to sustain care. The moderate QoC profile, which represented the largest caregivers group, demonstrated adequate performance in daily life care tasks but exhibited insufficient emotional support. While basic life needs were reasonably well met, caregivers showed limited responsiveness to the PwD’s psychological needs, individual interests, or social participation. This task‑centered approach left considerable room for improvement in overall care quality. In contrast, the high QoC profile exemplified dignity-oriented care. Caregivers not only delivered high quality life support but also actively consider the PwD’s emotional needs, autonomy, and social engagement. This profile fully embodied the core principles of comprehensive, high-quality care provision.

These distinct profiles were underpinned by different configurations of background variables, stressors, and resources. Overall, our findings are largely consistent with Extended Stress Process Model [16], confirming that background variables, primary stressors, and caregiver resources significantly predict the quality of informal care,but did not find a similar impact from caregivers’ quality of life. Specifically, contrary to both the model and prior research [7,16,41], caregiver depression, a core dimension of quality of life, did not show a significant association with QoC. This unexpected null finding may reflect mental health stigma in the Chinese cultural context, where caregivers often underreport depressive feelings due to fear of social disapproval or internalized expectations to be strong [42]. Such underreporting likely underestimates the true prevalence of depression and attenuates its statistical association with caregiving outcomes. Future research should therefore employ culturally adapted methodologies, such as destigmatizing interview protocols and implicit psychological measures, to reexamine this relationship and develop a culturally sensitive QoC assessment model. (line 335-370)

47. **This new wording could also discuss the possible associations between each profile and having to care for a person with dementia who was severely dependent for basic activities of daily living, or having to care for a person with dementia for more than five years, or what it means for each profile to have low economic resources to ensure the maintenance of the dependent person. In addition to the relationship of each profile with the independent variables included in this study.**

Thank you for this suggestion. We have added a brief discuss that contextualizes our profile findings within the specific caregiving situations you mentioned:severe ADL dependence, and low economic resources based on the observed associations in Table 6.

The low QoC profile was driven by a convergence of caregiver adversities: economic strain, high overload, and inadequate support, highlighting structural determinants of poor outcomes. This profile may be particularly common among caregivers of severely dependent PwD, those with prolonged care duration, or those struggling with limited financial resources to sustain care. (line 341-346)

48. **Finally, if the analyses suggested in the review of the Results section are carried out, associations may be observed between being a woman, having a lower level of education, having a lower economic status, having greater difficulty in coping with the costs of caring for the person with dementia, and having a poorer quality of life, as well as possibly having a poorer perception of health status and a higher probability of having depressive symptoms. If these hypotheses are confirmed, the authors should perhaps consider conducting an analysis by sex, assessing whether the cutoff point of 15 is valid for male and female caregivers, or whether there are differences in the cutoff point depending on sex.**

Thank you for this insightful suggestion. Following your proposed analytical approach, we examined associations between sex, education, economic status, and depressive symptoms. Our analyses confirmed that lower education and rural residence were associated with lower income, which in turn was significantly associated with higher depressive symptoms. These findings have now been incorporated into the Discussion section (lines 373-380).

We also conducted sex-specific ROC analyses to assess whether the ECS cutoff point differs by sex. However, no valid cutoff score for care quality could be established due to poor classification performance (AUC = 0.527). These results are provided in Appendix 2, Figure 2 for reference, but were not included in the main manuscript given the lack of empirical support for sex-specific thresholds.

**Specific comments on the submitted text:**

49. **Line 319: Regarding the sentence "Based on Extended Stress Model, this study examined how background variables, primary stressors, caregivers' quality of life, and caregiver resources predicted different profiles of quality of informal care," perhaps the authors, rather than stating what the study did, should interpret what the study found, for example, replacing the word "examined" with "allowed us to observe," if the authors believe that this expression is more appropriate for what they are trying to express. In addition, link the second sentence of this paragraph to the first, using, for example, "On the other hand, based on the Extended Stress Model..."**

Thank you for your comment on Line 319. In response to the comment of Reviewer 6, I have rewritten this section.The relevant section no longer appears in the manuscript.

50. **Line 325: Regarding the sentence "The cut-off point of the ECS was determined at 15 using ROC analysis, providing an initial reference standard for future screening and intervention studies," this last sentence of the first paragraph of the Discussion section needs to be expanded upon. The authors should try to explain what this new cut-off point implies for the existing situation, both clinically and in terms of social services planning.**

Thank you for this important suggestion to elaborate on the practical implications of our findings.

This cut‑off value enables healthcare professionals to rapidly identify caregivers at high risk of providing inadequate care, prompting timely evaluation and follow‑up. For social services planning, this benchmark supports the efficient allocation of resources by prioritizing interventions for the most vulnerable caregiver-PwD dyads. ( line 424-427)

51. **Line 340: The first two sentences of this third paragraph of the manuscript are redundant. It is recommended that the authors summarize them into a single sentence to make the manuscript easier to read.**

Thank you for this suggestion to improve conciseness. We have merged the two redundant sentences into one, as recommended.

Overall, our findings are largely consistent with Extended Stress Process Model [16], confirming that background variables, primary stressors, and caregiver resources significantly predict the quality of informal care,but did not find a similar impact from caregivers’ quality of life. (line 357-360)

52. **Line 363: The wording of the fifth paragraph of the Discussion section could be improved. ADLs are defined by the ability to perform those activities. Therefore, the wording should be along the lines of: "At the same time, when patients with dementia had a greater ability to perform basic activities of daily living, this situation correlated positively with the quality of care, while caregiver burden showed a negative correlation, results that have also been observed in a large number of studies [7,40,41,42,44]." . Caregivers experience high levels of stress and overload in the long-term care of older adults with dementia [45], while at the same time having to cope with family and occupational responsibilities. Full-time, long-term care impairs caregivers' mental health and their ability to provide care, further diminishing their quality of life [46,47]. Added to this is the progressive deterioration of the functional capacity of the person with dementia to perform basic activities of daily living, which also contributes significantly to increasing caregiver overload [48]. Or similar wording, depending on the authors' perspectives.**

Thank you for providing such clear and constructive wording suggestions for this paragraph. We have revised the manuscript accordingly.

At the same time, when PwD have a greater ability to perform basic activities of daily living, this situation correlated positively with the quality of care, while caregiver burden showed a negative correlation, results that have also been observed in a large number of studies [7,43,44,47,49]. Caregivers of PwD experience high levels of stress and overload in long-term care [50], compounded by competing family and occupational responsibilities. Full-time, long-term care impairs caregivers’ mental health and care capacity, further diminishing quality of care [51,52]. ( line 392-398)

53. **Line 370: To make the manuscript easier to read, it would be advisable to divide long paragraphs using full stops. We suggest dividing this paragraph into this point**

Thank you for your suggestion. We have divided the indicated long paragraph into shorter ones as recommended. ( line 399)

54. **Line 370: When dividing the paragraph at this point, the authors should improve the connection between paragraphs by using bridging phrases, such as: "In addition to the factors mentioned, other factors also play a role in this evolutionary process. Research has shown that cognitive impairment..." .**

Thank you for your suggestion.

In addition to the caregiver-related factors, patient-level characteristics also play a critical role in shaping QoC. ( line 399)

55. **Line 375: The authors should improve the connection between sentences. At this point, for example, among other options, they could use: "This situation is also contributed to by the fact that progressive disability is often accompanied by aphasia and comprehension deficits..." .**

Thank you for the precise suggestion to improve the sentence connection. We have revised the text at line 375 using the provided phrasing: This challenge is compounded by the fact that progressive disability is often accompanied by aphasia and comprehension deficits [3], making it difficult for patients to express their needs accurately. These challenges can undermine caregivers’ patience and lead to inadequate care [54]. ( line 405-408)

56. **Line 377: The wording of this paragraph could be improved. The authors should try to present their discussion by linking the various interpretations of the results obtained and their relationship to other observations made by other authors. In relation to this paragraph, an alternative wording might be: "These challenges can undermine caregivers’ patience and lead to inadequate care, so that as the ability of people with dementia to perform ADLs declines, the quality of care may also potentially decline. Therefore, assessing the ability of people with dementia to perform ADLs is an indispensable tool for providing individualized care and for proposing measures to increase these abilities, all with the goal of improving their quality of life and that of their caregivers". NOTE: Look for bibliography to support these aspects. This wording can be modified at the author’s discretion.**

Thank you for the detailed alternative wording and guidance. We have revised as suggested. We have also added supporting references at the indicated points to substantiate the discussion.

These challenges can undermine caregivers’ patience and lead to inadequate care [54]. Consequently, as PwD’s ADL decline, QoC may also decrease. Therefore, assessing the ability of PwD to perform ADLs is an indispensable tool for providing individualized care and for proposing measures to increase these abilities, all with the goal of improving the quality of care for PwD. (line 407-412)

**5. Conclusion**

57. **Line 434: The phrase "better pre-morbid relationships and superior ADL of PwD were predictors of high QoC" could be improved. An alternative wording would be: "better premorbid relationships and greater ability to perform activities of daily living (ADL) among people with dementia were predictors of a high quality of life."**

Thank you for this suggestion to improve the precision of the wording. We have revised the sentence accordingly. To maintain consistency with the focus of our study, we retained the term "quality of care" in this context, as it is the central outcome variable in our analysis regarding predictors of caregiving profiles. The revised sentence now reads: "…better pre-illness relationship quality and greater ability to perform ADL of PwD were key predictors of high QoC." (line 454-455)

58. **Line 434: Perhaps the appropriate word is "heterogeneous" rather than "homogeneous."**

Revised. (line 458)

59. **Line 441: It may be necessary to include new conclusions if the analyses suggested in the previous sections are carried out.**

We have revised the Conclusion section to incorporate and reflect the new analyses performed throughout the manuscript as suggested by the reviewers.
