# Supplementary material for: Quality of informal care among informal caregivers of people with dementia: A latent profile and ROC analysis
Source: PLoS One. 2026 Apr 8;21(4):e0346557. doi: 10.1371/journal.pone.0346557 (PMC13061180; doi:10.1371/journal.pone.0346557)
Supplement: Attachment — Submitted filename: Response to Editor.doc [file pone.0346557.s011.doc]

Response to Editor

Dear Editor,

Thank you for your detailed and constructive comments on my manuscript. According to the editor and reviewers’ comments, we have made extensive corrections to our previous manuscript. Two versions of the revised manuscript are submitted: one with no markings and another with the changes highlighted in yellow for clarity. Below are our point-by-point responses to the editor, the red font is the revised original text in the manuscript:

Journal Requirements:
**1. Please review your reference list to ensure that it is complete and correct. If you have cited papers that have been retracted, please include the rationale for doing so in the manuscript text, or remove these references and replace them with relevant current references. Any changes to the reference list should be mentioned in the rebuttal letter that accompanies your revised manuscript. If you need to cite a retracted article, indicate the article’s retracted status in the References list and also include a citation and full reference for the retraction notice.**

Thank you for this important reminder. We have carefully reviewed the entire reference list to ensure its completeness and accuracy. All in-text citations have been verified against the reference list to confirm that no citations are missing and that all listed references are cited in the text. We have consulted PubMed to confirm that none of the cited papers have been retracted. All necessary corrections have been made in the revised manuscript, and we believe the reference list now meets the journal's standards.

**Additional Editor Comments:**
**1. Thank you very much for submitting the revised manuscript titled “Quality of informal care among informal caregivers of people with dementia : a latent profile and ROC analysis” after incorporating the suggestions from the editor and the reviewers. After carefully going through the revised manuscript and the responses to reviewers’ comments, I still have some concerns, addressing those, I believe, would enhance the quality of the manuscript up to the standard of publication for the journal.**

Thank you very much for the opportunity to further revise our manuscript. We have carefully addressed all the remaining concerns raised by you and the reviewers. The point-by-point responses are provided below, and all revisions are clearly marked in the manuscript. We believe the revised manuscript is now suitable for publication and look forward to your final decision.

**2. I would like to request the authors to strictly follow the instructions for authors while preparing tables and figures, and how the in-text citation to those tables and figures have been made. Many of the tables misses the key elements in titles, footnotes, and legends. We would not be able to accept a manuscript that which has not been prepared strictly following the instructions to authors. You may consider of taking help from the experts for preparing the manuscript.**

Thank you very much for your valuable feedback. We sincerely apologize for the formatting issues in our tables and figures. We have now carefully reviewed the journal’s “Instructions for Authors” and revised all tables and figures accordingly. Specifically, we have: Added complete titles to all tables and figures. Included necessary footnotes and legends to explain abbreviations and symbols. Corrected in-text citations to ensure they follow the journal’s style. We also sought assistance from a colleague experienced in manuscript preparation to verify compliance. We believe the revised manuscript now fully meets the journal’s formatting standards.

**3. Some tables in the appendices seem redundant. The table numbers in the manuscript and the appendices are overlapping, titles and notes are incomplete, and without any reference to the text of the manuscript. Some column headings are given in complete lower case. Requesting authors to be careful while preparing the manuscript even if those are supporting materials. Appendices are not meant for dumping of information and analyses, but to provide readers the context without confusing them.**

Thank you for your detailed review of the appendices. We have carefully revised all supplementary materials according to your suggestions:

First, to avoid numbering overlap with the main text, the remaining appendices have been renumbered as S1 Table, S2 Table, and S1 Fig, with corresponding citations now added in the main manuscript.

Second, complete titles and detailed notes have been added to each table and figure, and all column headings have been corrected to proper case.

Third, regarding Appendix 2, we acknowledge that this appendix, which was created to address specific comments from Reviewer 6, did not provide meaningful information relevant to the main findings. Since these analyses are not cited in the main text, we have decided to remove Appendix 2 entirely to streamline the supplementary materials and avoid redundancy.

**4. Method: Sample size calculation. Please remove the response rate from the paragraph. I believe all 248 pair of caregivers and PwD responded to the study, but the current analyses included data from 213 out of 248 pairs. This is not the response rate as we experience in survey where not all persons are approached may not respond.**
Thank you for this important clarification. We agree that “response rate” is not the appropriate term in this context. We have revised the sentence to accurately reflect that the 213 dyads represent the final analytic sample. The revised text now reads:

Initially, 248 caregiver-PwD dyads were recruited. After excluding 35 dyads due to incomplete responses or other reasons, 213 valid dyads were included in the final analysis. (line 176-178)

**5.Fig 2. Please mention the name of the score / scale, of which the cut-off of 15 was estimated, in the title of ROC curve.**
Thank you for your suggestion. We have revised the title of Fig. 2 to clearly indicate the name of the scale used to estimate the cut-off value of 15. The revised title now reads: "Fig. 2. ROC curve of the Exemplary Care Scale for classifying low and high quality of care." (line 324)

6. **Appendix-2 title of the ROC curve. Font not in English. No title of the figure. The ROC curve looks completely different what is provided in the manuscript (Fig 2). What is the full form of AUC.**

Thank you for reviewing Appendix 2. This ROC curve was created in response to Reviewer 6's suggestion (Comment #48) to explore whether the ECS cutoff differs by sex. We conducted sex-specific ROC analyses using QoC as the classification variable. However, classification performance was poor (The area under the curve, AUC = 0.527), and no valid sex-specific cutoff could be established. Since these analyses did not provide meaningful information relevant to our main findings, and to avoid redundancy as per your guidance, we have decided to remove Appendix 2 entirely from the revised manuscript.

**7. Table 8. Please include the name of the variable in the title of the table (like you did for Table 5) of which the mean and SD have been calculated.**
Thank you for your guidance. The change has been marked in the revised manuscript. Table 8. Negative and positive groups classified by the optimal cut-off score of the Exemplary Care Scale. (line 325)

**8. Specifically, contrary to both the model and prior research [7,16,41], caregiver depression, a core dimension of quality of life, did not show a significant association with QoC. Further, while explaining such non-significant association between depression and QoC author questioned the validity of the scale of depression in Chinese population, and inferred that the depression may be under-reported and that underreporting could be due to fear of social disapproval or strong internalized expectations among Chinese culture. At this point there are two situations. Either, the depression scale is not valid for the Chinese population, and we should not use the variable at all, or may be the non-significant result is the significance of the association. May be the caregivers are providing good quality of care to the PwD, as it is the cultural practice to care about them, in spite of themselves feeling depressed in providing care due to different socioeconomic constraints.**

Thank you for this insightful observation. We acknowledge that there was a lack of clarity regarding the role of depression, which created confusion. We agree with the reviewer that simply dismissing the depression scale as invalid would be inconsistent with the data. We have softened this claim. Instead, we now argue that cultural factors may lead to either under-reporting or a decoupling of depressive feelings from caregiving behaviors. We have now substantially revised the Discussion section to address this:

This nonsignificant association can be attributed to Chinese cultural norms, particularly filial piety and family responsibility. This cultural value has been linked to reduced caregiver burden and enhanced QoC [42], as home-based dementia care in China is rooted in familism and filial norms [43]. Within this context, family caregivers may find fulfillment in their caregiving role [44], reinforced by community recognition, which enables them to sustain caregiving standards despite experiencing depressive symptoms. This mechanism explains our core finding: although depressive symptoms differed significantly across the three latent profile groups (S1 Table), they did not predict QoC. In other words, depression effectively captured caregiver heterogeneity, yet its impact on caregiving behavior was buffered by cultural responsibility. Thus, the observed nonsignificant association reflects the unique cultural context of family caregiving in China, rather than any inadequacy in scale validity. Future research should develop culturally sensitive models for assessing QoC. (line 355-368)
